# Supplementary material for: Subclone-specific microenvironmental impact and drug response in refractory multiple myeloma revealed by single‐cell transcriptomics
Source: Nat Commun. 2021 Nov 29;12:6960. doi: 10.1038/s41467-021-26951-z (PMC8630108; doi:10.1038/s41467-021-26951-z)
Supplement: Supplementary file 1 — Supplementary information. [file 41467_2021_26951_MOESM1_ESM.pdf]

Supplementary Information

Subclone-specific microenvironmental impact and drug response in refractory multiple myeloma revealed by single cell transcriptomics

Stephan M. Tirier, Jan-Philipp Mallm, Simon Steiger, Alexandra M. Poos, Mohamed H. S. Awwad, Nicola Giesen, Nicola Casiraghi, Hana Susak, Katharina Bauer, Anja Baumann, Lukas John, Anja Seckinger, Dirk Hose, Carsten Müller-Tidow, Hartmut Goldschmidt, Oliver Stegle, Michael Hundemer, Niels Weinhold, Marc S. Raab, Karsten Rippe

Inventory of Supplementary Information

Supplementary Figures

Supplementary Fig. 1. Quality control of single cell transcriptome data
Supplementary Fig. 2. Copy number alteration (CNA) analysis from scRNA-seq data
Supplementary Fig. 3. Non-malignant plasma cells, cytogenetics and +1q signature
Supplementary Fig. 4. Cell types in the bone marrow environment
Supplementary Fig. 5. Predicted cellular interactions
Supplementary Fig. 6. Cell type abundance changes upon treatment and pDC reprogramming
Supplementary Fig. 7. T-cell subtype characterization
Supplementary Fig. 8. Analysis of CD16$^+$ monocytes
Supplementary Fig. 9. FACS validation of scRNA-seq data for NK and TAM3 cells
Supplementary Fig. 10. Characterization of inflammatory cDC2 population

Supplementary Tables

Supplementary Table 1. Overview of RRMM patients
Supplementary Table 2. Cell numbers of samples studied
Supplementary Table 3. Cell types and marker genes
Supplementary Table 4. Gene expression signatures
Supplementary Table 5. Data analysis software
Supplementary Table 6. Antibodies for fluorescence activated cell sorting
Supplementary Table 7. Inventory of supplementary data sets

Supplementary Data Sets

Additional data sets on samples and the analysis results derived from the different sequencing readouts are provided as separate files in Microsoft Excel format. An inventory for these data sets is given in Supplementary Table 7.

Supplementary Figures

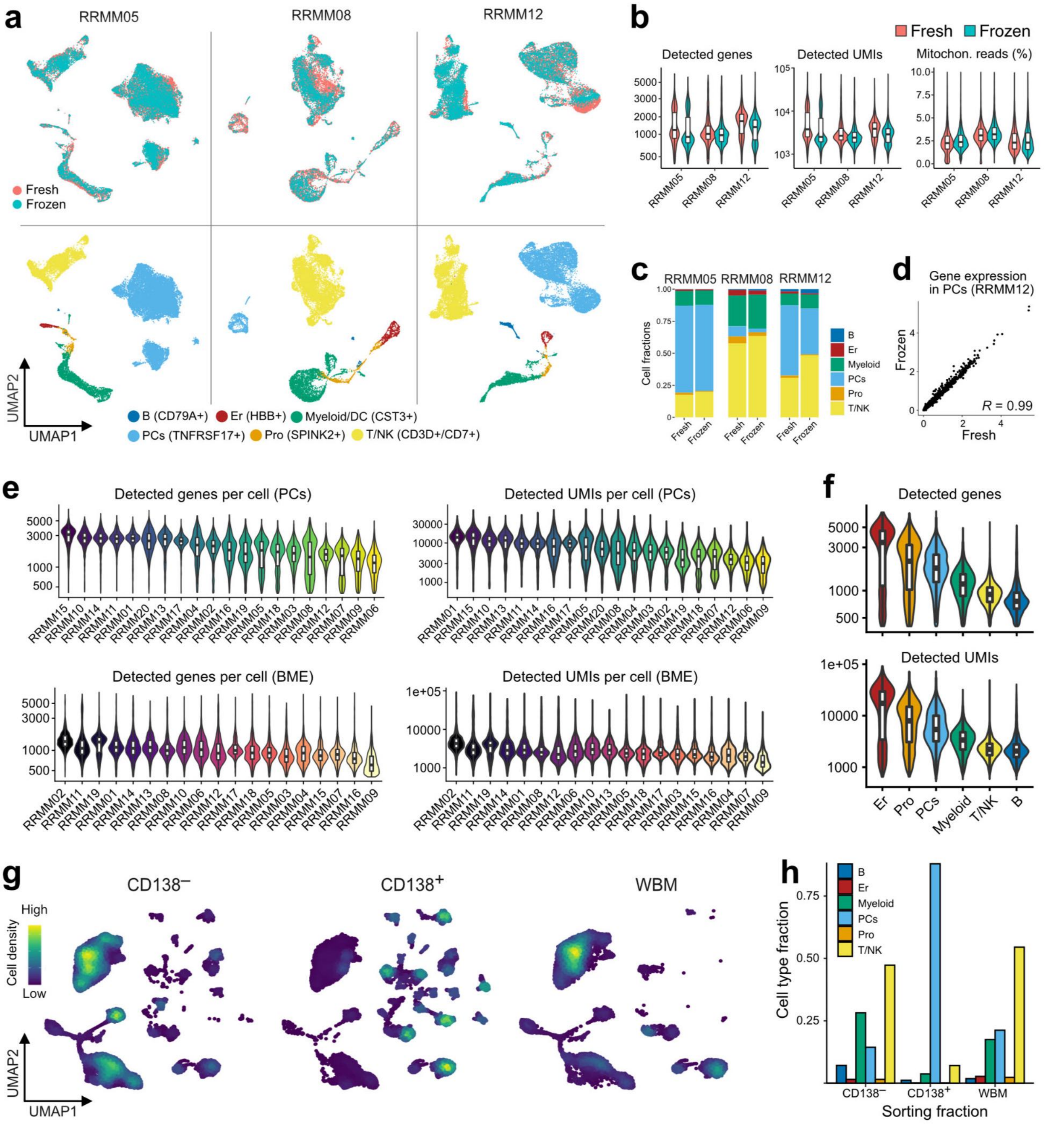

Supplementary Fig. 1. Quality control of single cell transcriptome data

For cell numbers see Supplementary Table 2 and Supplementary Data Set 1. (**a**) UMAP embedding of three patients without batch effect correction. Top, colored by fresh vs. frozen processing; bottom, colored by major cell type. (**b**) Violin plots of scRNA-seq quality metrics for processing of fresh vs. frozen cells. (**c**) Stacked bar plot of relative abundances of major cell types (fresh vs. frozen). (**d**) Scatter plot of averaged genes expression of plasma cells (PCs) in RRMM12 (fresh vs. frozen). A Pearson's correlation coefficient of $R = 0.99$ was obtained. (**e**) Violin plot of quality metrics per patient for PCs (top) and for BME cells (bottom). (**f**) Violin plot of detected genes and UMIs per major cell type. (**g**) UMAP embedding as shown in Fig. 1C as point-density plot split into the three different sorting fractions. (**h**) Bar plot of major cell type fractions per sorting fraction. Box plot: center line, mean; box limits, first and third quartile; whiskers: minimum/maximum values.

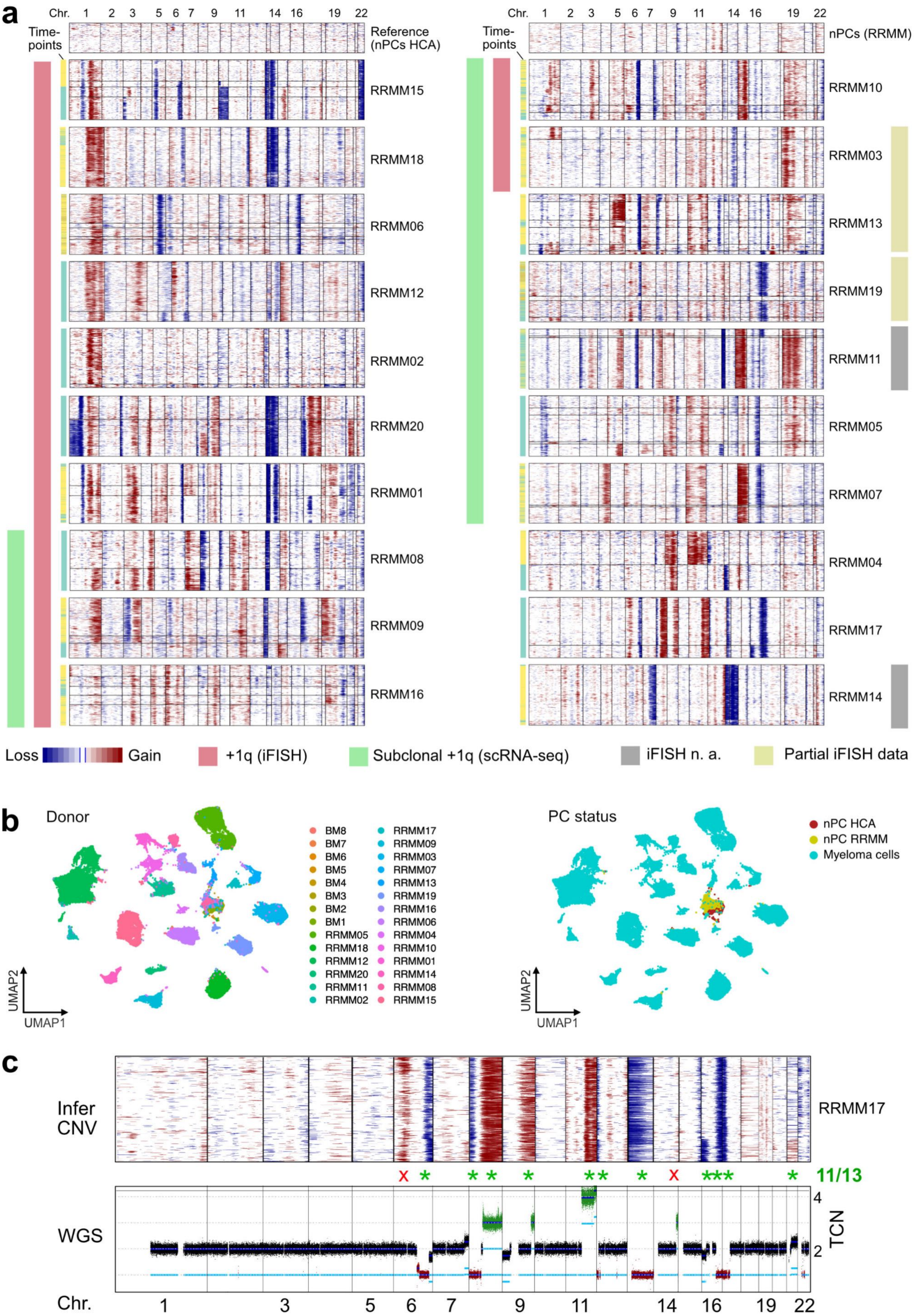
a
Chr. 1 2 3 5 6 7 9 11 14 16 19 22
Time-points
Reference (nPCs HCA)
RRMM15
RRMM18
RRMM06
RRMM12
RRMM02
RRMM20
RRMM01
RRMM08
RRMM09
RRMM16
nPCs (RRMM)
RRMM10
RRMM03
RRMM13
RRMM19
RRMM11
RRMM05
RRMM07
RRMM04
RRMM17
RRMM14
Loss
Gain
+1q (iFISH)
Subclonal +1q (scRNA-seq)
iFISH n. a.
Partial iFISH data
b
Donor
BM8
BM7
BM6
BM5
BM4
BM3
BM2
BM1
RRMM05
RRMM18
RRMM12
RRMM20
RRMM11
RRMM02
RRMM17
RRMM09
RRMM03
RRMM07
RRMM13
RRMM19
RRMM16
RRMM06
RRMM04
RRMM10
RRMM01
RRMM14
RRMM08
RRMM15
UMAP2
UMAP1
PC status
nPC HCA
nPC RRMM
Myeloma cells
c
Infer CNV
RRMM17
11/13
WGS
TCN
4
2
Chr. 1 3 5 6 7 9 11 14 16 19 22

Supplementary Fig. 2. Copy number alteration (CNA) analysis from scRNA-seq data

(**a**) Heatmap of CNA signal per patient normalized against non-malignant plasma cells (nPCs) derived from the HCA bone marrow reference data set using the InferCNV package. Horizontal lines divide subclones. Top left, nPC reference from HCA data set; top right, nPCs from RRMM samples. (**b**) UMAP of PCs derived from RRMM patients and healthy donors (HCA) combined. Left, colored by donor; right, colored by plasma cell status. (**c**) CNAs of exemplary patient sample RRMM17. Top, heatmap of RRMM17 CNA signal normalized against nPCs derived from the HCA bone marrow reference data set with the horizontal lines divide subclones; bottom, coverage plot showing total copy number derived from whole genome sequencing data of the same sample; * indicates agreement between both modalities in detecting major CNAs; TCN, total copy number.

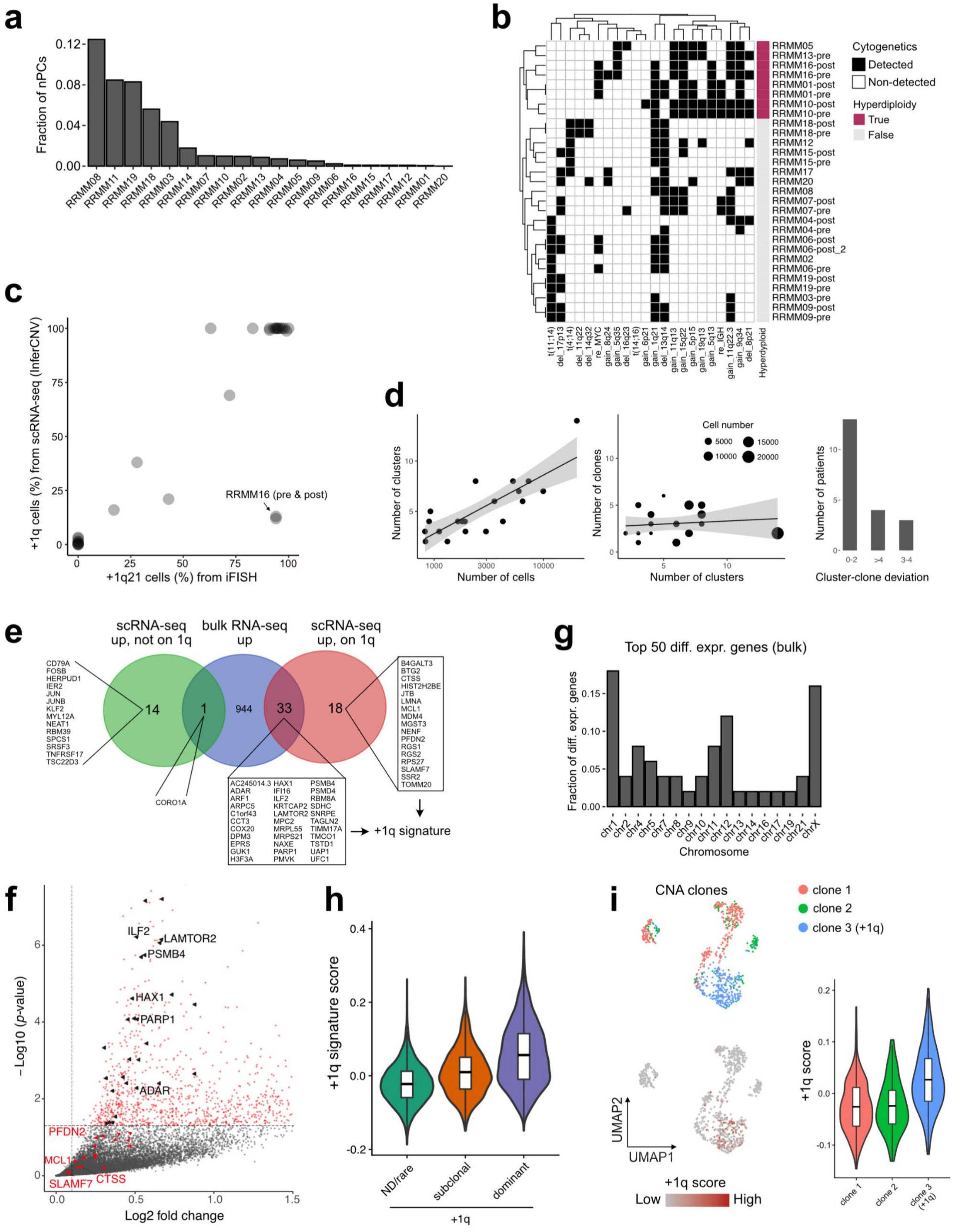

Supplementary Fig. 3. Non-malignant plasma cells, cytogenetics and +1q signature

(**a**) Bar plot of non-malignant plasma cell (nPC) abundances per patient. (**b**) Hierarchical clustering of binary cytogenetic information per sample. (**c**) Scatterplot showing fraction of +1q cells as detected by scRNA-seq vs iFISH. The Pearson's correlation coefficient was R = 0.86 (p = $1.6 \cdot 10^{-9}$). (**d**) Left and middle: Scatterplots showing relationships between number of cells, clusters and CNA clones

per patient. Regression line and 95% confidence interval are indicated. The Pearson's correlation coefficient was $R = 0.81$ ($p = 1.6 \cdot 10^{-5}$, clusters vs. cells) and $R = 0.13$ ($p = 0.58$, clones vs. clusters). Right: Bar plot showing deviation of cluster and clone numbers per patient subdivided in three groups (n = 0-2, 3-4, >4). (**e**) Venn-diagram depicting the overlap between genes upregulated in +1q clones using scRNA-seq and genes identified in +1q patients with an independent bulk RNA-seq data set. The latter was from newly diagnosed multiple myeloma patients classified into 1q21 detected vs. not-detected based on cytogenetics. (**f**) Volcano plot of differentially upregulated genes computed with DESeq2 [1] in +1q patients from bulk RNA-seq data [2]. The red dots indicate genes above the thresholds indicated by the dashed lines of $p < 0.05$ (Bonferroni-adjusted) and a log2 fold change (logFC) > 0.1. (**g**) Bar plot showing fraction of the top 50 differentially upregulated genes by chromosomal location in +1q patients compared to patients without gain of 1q. (**h**) Violin plot of +1q signature scores for the three different +1q groups and cell numbers of 12,635 (ND/rare), 1,869 (subclonal) and 2,505 (dominant). Data were taken from Ledergor et al.[3] with Bonferroni-adjusted p-values $< 2 \cdot 10^{-16}$ from a Kruskal-Vallis-test. (**i**) Analysis of donor AL04 [3]. Clone 1 (red, n = 318), clone 2 (green, n = 126) and clone 3 (blue, n = 292, +1q) were defined based on the CNA analysis and plotted in the UMAP on the top. Clone 3 with +1q can also identified based on its +1q transcription signature score. This is shown in the UMAP plot (bottom) and the Violin plot (right) that shows the +1q signature scores for the different CNA clones with Bonferroni-adjusted p-values $< 2 \cdot 10^{-16}$ from a Kruskal-Vallis-test. Box plot: center line, median; box limits, first and third quartile; whiskers, minimum/maximum values.

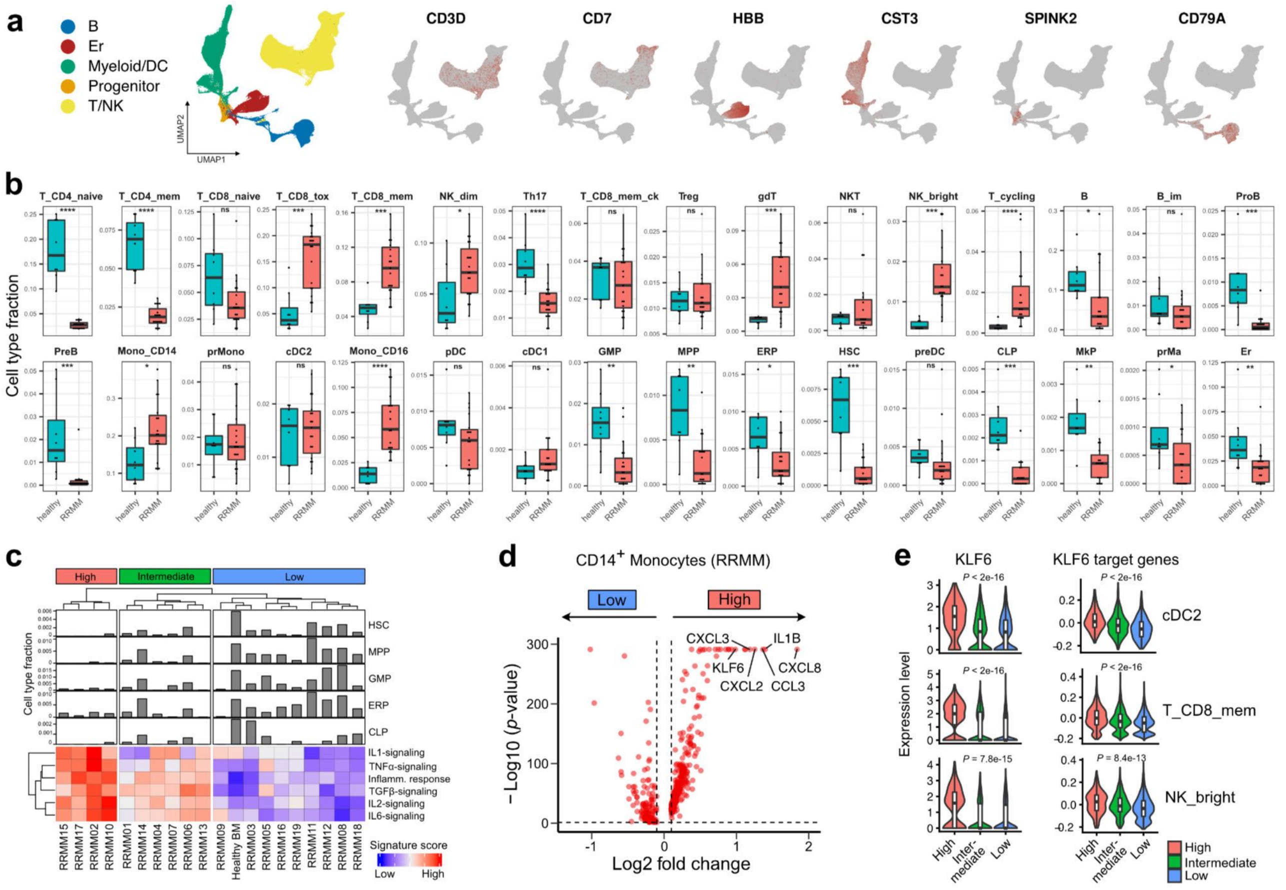

Supplementary Fig. 4. Cell types in the bone marrow environment

(**a**) UMAP embedding of $CD138^{-}$ cells of the bone marrow environment (BME) colored by major cell type and by expression of indicated marker genes. (**b**) Box plot for the comparison of cell type fractions between RRMM patients (n = 19) and healthy individuals (n = 8). The Bonferroni adjusted p-values from a two-sided Wilcoxon rank-sum test are indicated with ns, $p > 0.05$; *, $p < 0.05$; **, $p < 0.01$; ***, $p < 0.001$; ****, $p < 0.0001$. Box plot: center line, median; box limits, first and third quartile; whiskers, minimum/maximum values. (**c**) Heatmap of average immune signaling pathway signature scores of BME cell types from individual patients and healthy donor reference. The heatmap is split according to the dendrogram (k = 3) into three groups with high, intermediate and low scores for the indicated signaling pathways. The bar plots display the relative abundance of the different progenitor cell types in the BME. (**d**) Volcano plot of differential expression analysis between $CD14^{+}$ monocytes derived from "low" and "high" patient groups shown in panel *c*. Thresholds for differential expression were p-value < 0.05 from a Bonferroni-adjusted Wilcoxon rank-sum test and logFC > 0.1 or < -0.1, respectively. (**e**) KLF6 expression levels (left) and KLF6 target gene module scores (right) are shown as Violin plots of the immune signaling groups defined in panel *c* in cDC2 (high, n = 241; intermediate, n = 1,010; low, n = 590), $CD8^{+}$ memory effector T-cell (high, n = 1,267; intermediate, n = 7,133; low, n = 4,684) and NK^{bright} cell (high, n = 237; intermediate, n = 1,274; low, n = 504) populations. Bonferroni-adjusted p-values are from a Kruskal-Vallis-test. Box plot: center line, median; box limits, first and third quartile; whiskers, minimum/maximum values.

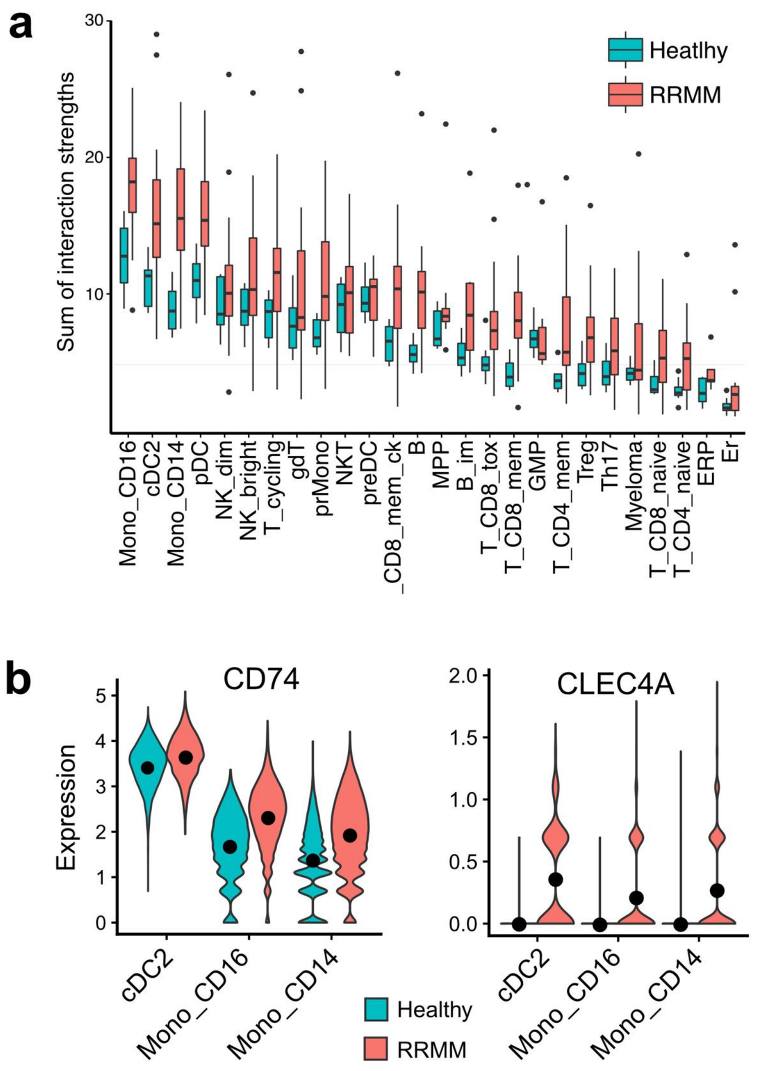

Supplementary Fig. 5. Predicted cellular interactions

(**a**) Box plot of interaction strength in RRMM (n = 19) and healthy (n = 19) individuals. The sum of interaction strengths as mean expression of ligand and receptor of nPCs (HCA) or myeloma tumor cells (RRMM) and the indicated immune cell types per sample was plotted. Box plot: center line, median; box limits, first and third quartile; whiskers, minimum/maximum values. (**b**) Violin plots of CD74 and CLEC4A gene expression levels in myeloid cell types in RRMM vs healthy individuals. Center-points indicate mean values.

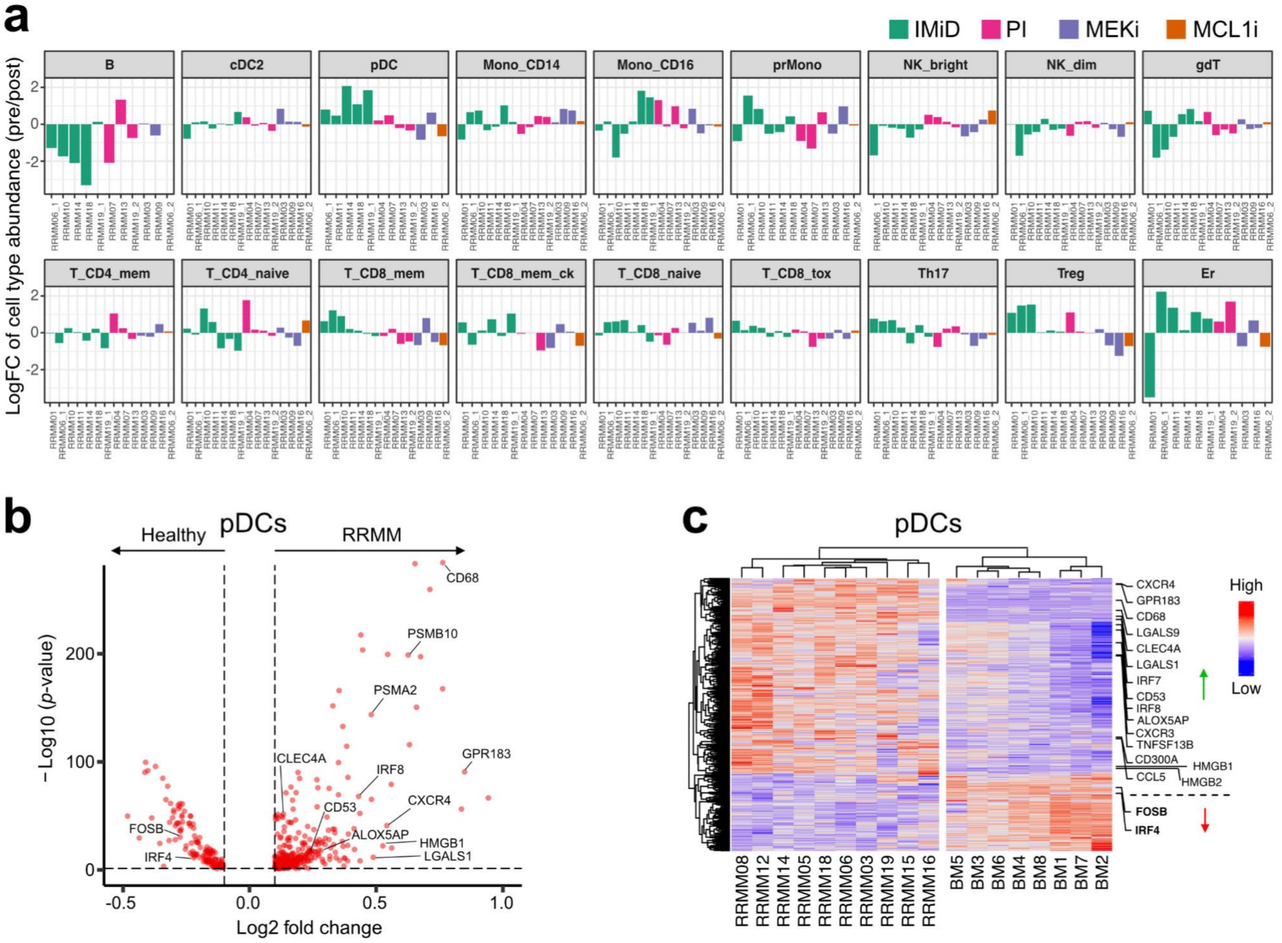

Supplementary Fig. 6. Cell type abundance changes upon treatment and pDC reprogramming

(**a**) Bar plots of relative log fold changes in cell type abundances during treatment for the indicated patient samples. Only the most abundant cell types are shown. (**b**) Volcano plot of differentially expressed genes (RRMM vs. healthy) in pDCs. Thresholds for differential expression were *p*-value < 0.05 from a Bonferroni-adjusted Wilcoxon rank-sum test and logFC > 0.1 or < -0.1, respectively. (**c**) Heatmap showing clustered average genes expression levels of differentially expressed genes between pDCs in RRMM vs. healthy per patient/donor. Selected genes are listed on the left side of the heatmap. Only patient samples with >25 profiled cells are shown.

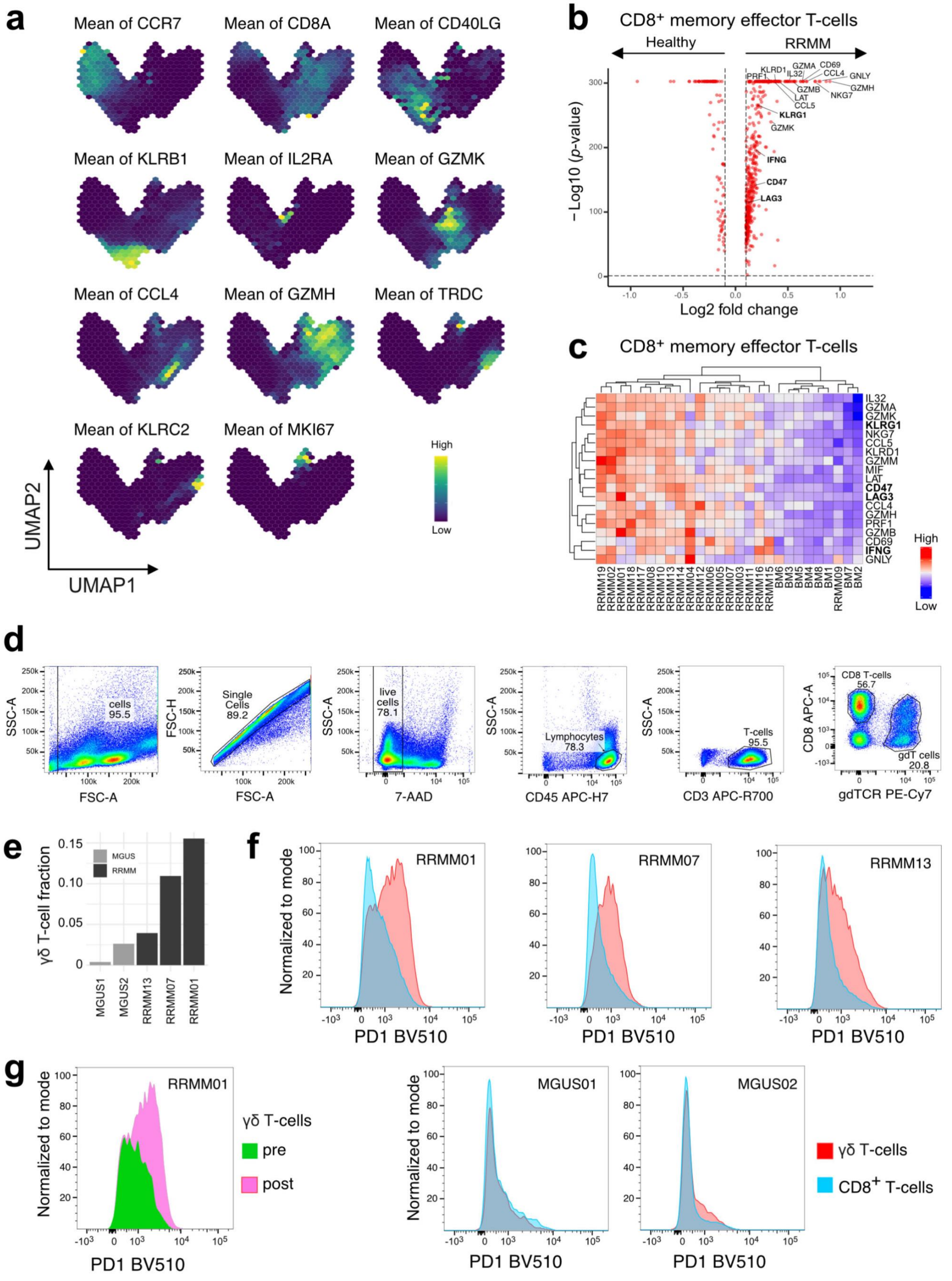

Supplementary Fig. 7. T-cell subtype characterization

(**a**) UMAP plot of expression levels of T-cell subtype marker genes. Gene expression of cells in similar locations are binned in hexagons and averaged. (**b**) Volcano plot of differentially expressed

genes (RRMM vs. healthy) in $CD8^+$ memory effector T-cells. Thresholds for differential expression were *p*-value < 0.05 from a Bonferroni-adjusted Wilcoxon rank-sum test and logFC > 0.1 or < -0.1, respectively. (**c**) Clustered heatmap of selected differentially expressed genes in $CD8^+$ memory effector T-cells in RRMM patient vs. healthy donor samples. (**d**) FACS gating strategy for the analysis of γδ T-cells and $CD8^+$ T-cells. (**e**) Bar plot showing fractions of γδ T-cells in RRMM samples. For comparison samples from MGUS (monoclonal gammopathy of undetermined significance) patients were also included in the analysis. (**f**) FACS histograms for PD1 protein expression levels in γδ T-cells vs. $CD8^+$ T-cells in MGUS and RRMM samples. (**g**) FACS histograms for protein expression levels of PD1 in γδ T-cells pre/post treatment of patient RRMM01.

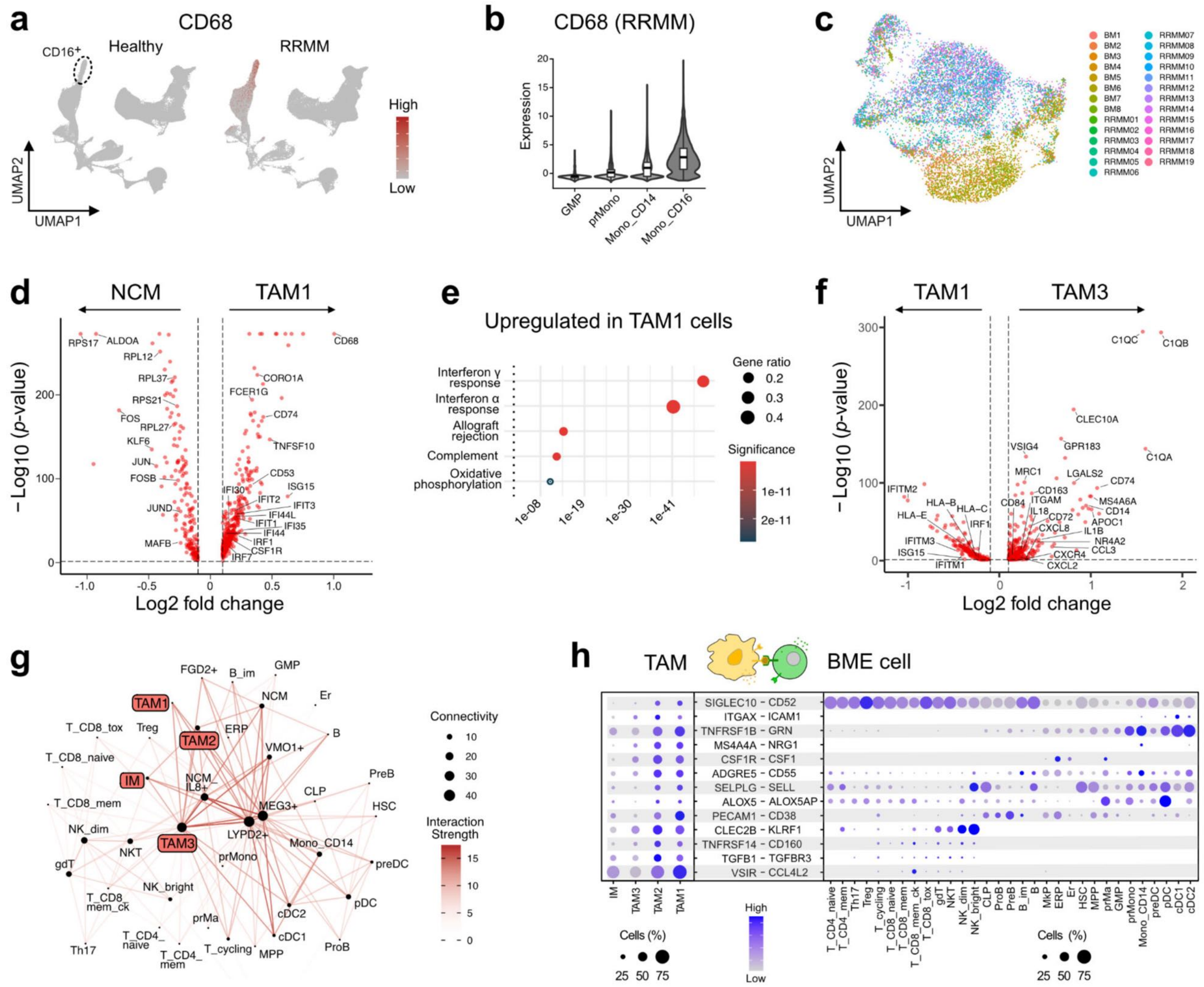

Supplementary Fig. 8. Analysis of CD16⁺ monocytes

(**a**) UMAP embedding of CD68 gene expression levels split in healthy donors and RRMM. (**b**) Violin plot of CD68 expression levels in the myelomonocytic lineage. Box plot: center line, median; box limits, first and third quartile; whiskers, minimum/maximum values. (**c**) UMAP embedding of subclustered CD16^{+} monocytes colored by patient/donor. (**d**) Volcano plot of differentially expressed between non-classical monocytes (NCM) and TAM1 subtypes. Thresholds for differential expression were *p*-value < 0.05 from a Bonferroni-adjusted Wilcoxon rank-sum test and logFC > 0.1 or < -0.1, respectively. (**e**) Gene set enrichment dot plot using hallmark gene sets of differentially upregulated genes in TAM1 cells vs. NCM. (**f**) Volcano plot of differentially expressed genes between TAM1 and TAM3 subtypes. Thresholds for differential expression were *p*-value < 0.05 from a Bonferroni-adjusted Wilcoxon rank-sum test and logFC > 0.1 or < -0.1, respectively. (**g**) Network plot of predicted cellular interactions between immune cell subsets in healthy donors. TAM/IM populations were assigned to the TAM1-3 populations in RRMM (Fig. 6) based on their transcriptome profile. These cell types were present at a much lower abundance in healthy donors as compared to the RRMM patients. In the network, every cell type is connected to its 4 top interacting cell types based on the sum of interaction strengths. The node size corresponds to the number of connected cell types and the coloring corresponds to the interaction strength. (**h**) Gene expression dot plot of ligand/receptors. RRMM-enriched CD16^{+} monocyte/macrophage subtypes (left) and associated interaction partners in the RRMM BME (right) are depicted. Only interactions are shown that were increased between TAM1/2 and immune cell types when compared to IM/TAM3 subtypes.

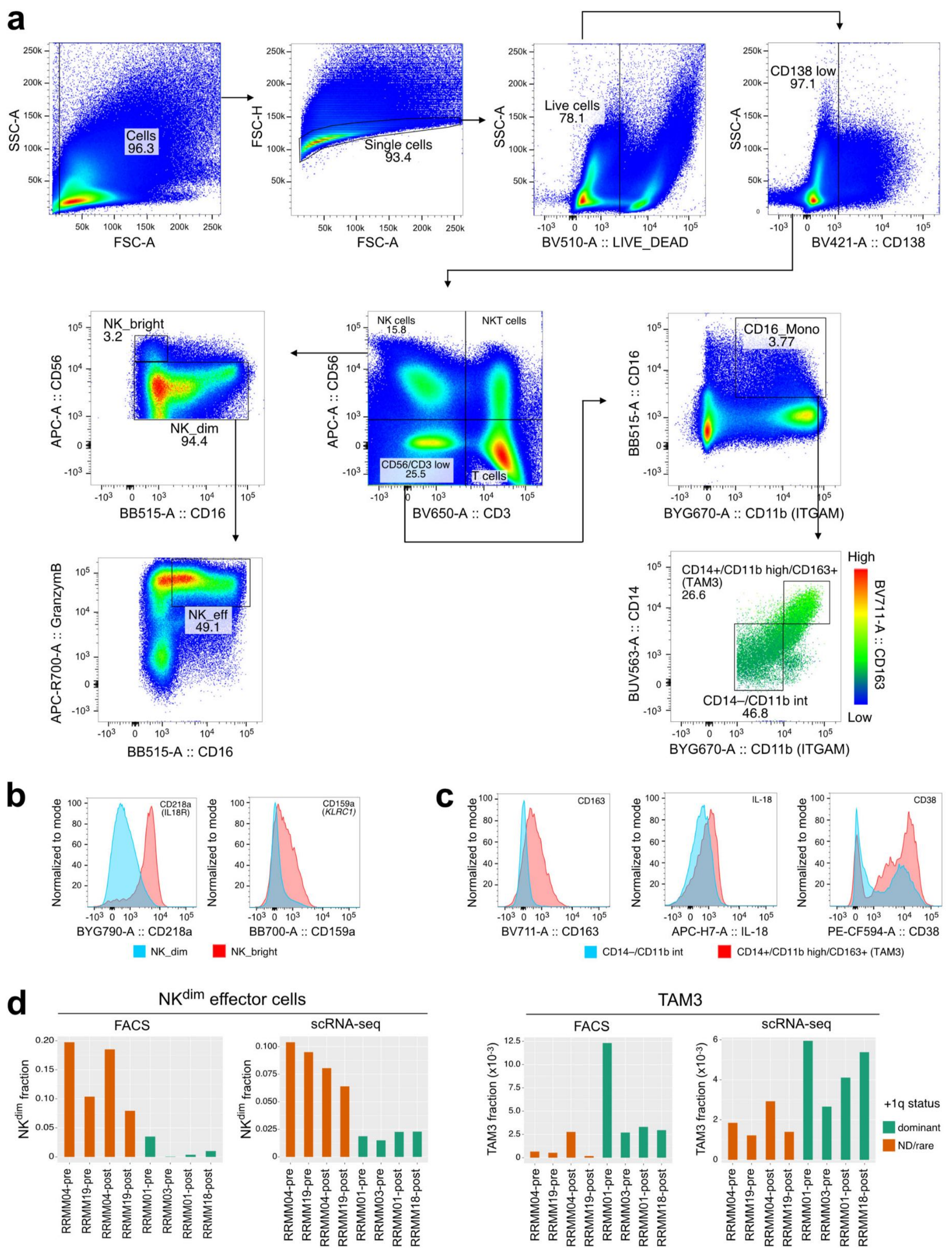

Supplementary Fig. 9. FACS validation of scRNA-seq data for NK and TAM3 cells

(**a**) FACS gating strategy for NK and TAM3 cells (eight samples combined). (**b**) FACS histograms for protein expression levels of CD218a (*IL18R1/IL18RAP*) and CD159 (*KLRC1*) for NK cells. (**c**) FACS histograms for protein expression levels of CD163, IL-18 and CD38 for CD16^{+} monocyte/TAM populations. (**d**) Bar plot showing TAM3 (CD14^{+}/CD11b^{Hi}/CD163^{+}) and NK^{dim} effector cell fractions in individual samples derived from FACS analysis as depicted in panel *a*. CD138^{-} cells represent the parental gate for calculating cell fractions, similar to the scRNA-seq data analysis.

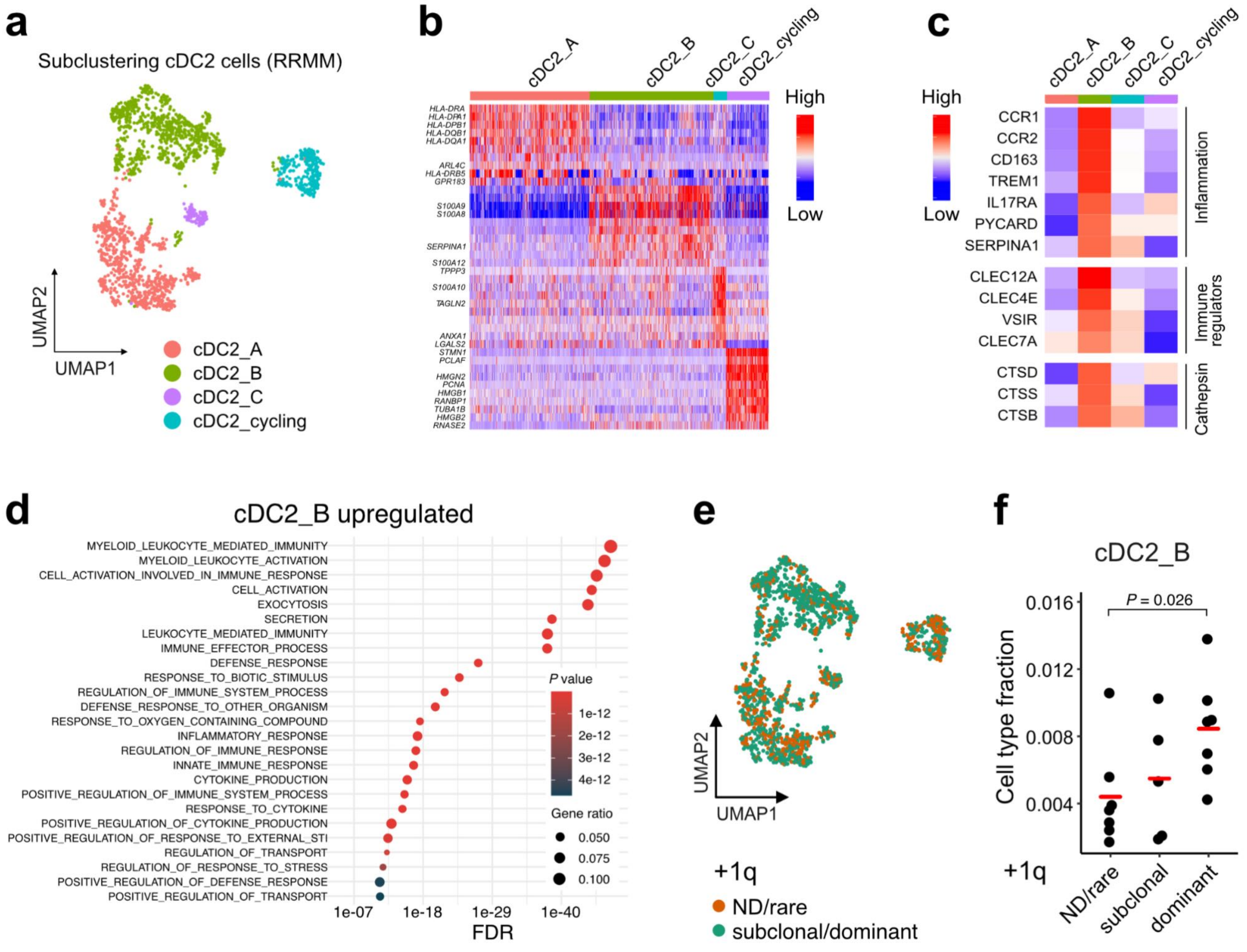

Supplementary Fig. 10. Characterization of inflammatory cDC2 population

(**a**) UMAP embedding of subclustered cDC2 cells colored by subtype. (**b**) Heatmap of top 10 genes that were differentially expressed in a given cDC2 subtype (*p*-value < 0.05 from a Bonferroni-adjusted two-sided Wilcoxon rank-sum test and logFC > 0.1 or < -0.1, respectively). (**c**) Heatmap showing average gene expression levels of selected genes that were upregulated in the inflammatory cDC2_B subtype (*p*-value < 0.05 from a Bonferroni-adjusted two-sided Wilcoxon rank-sum test and logFC > 0.1). (**d**) Gene set enrichment dot plot using GO gene sets of differentially upregulated genes in inflammatory cDC2_B subtype. (**e**) UMAP embedding of subclustered cDC2 cells colored by +1q status classified as ND/rare, subclonal or dominant. (**f**) Beeswarm plot for the comparison of inflammatory cDC2_B subtype fractions between the +1q groups ND/rare (*n* = 7 individuals), subclonal (*n* = 5 individuals) and dominant (*n* = 7 individuals). The Bonferroni-adjusted *p*-values from a two-sided Wilcoxon test are shown, and the red center line indicates the mean value.

Supplementary Tables

Supplementary Table 1. Overview of RRMM patients

Sample ID	Previous therapies	Group[a]	Drug (t1)[b]	Res-ponse[c]	Drug (t2)[b]	Res-ponse[c]	+1q State[d]
RRMM01	2	IMiD	Pom/Dex, anti-CD38	SD	–	–	high
RRMM02	3	NA	–	NA	–	–	high
RRMM03	2	MEKi	MEKi, BRAFi	PR	–	–	int
RRMM04	6	PI	Carfil/Dex	MR	–	–	low
RRMM05	1	NA	–	NA	–	–	low
RRMM06	3	IMiD	Len/Dex, anti-CD38	SD	MCL1i	PR	high
RRMM07	1	PI	Carfil/Dex, Cyclo-phosphamid	SD	–	–	low
RRMM08	6	NA	–	NA	–	–	int
RRMM09	3	MEKi	MEKi, BRAFi	CR	–	–	int
RRMM10	2	IMiD	Pom/Dex, HDACi	MR	–	–	high
RRMM11	4	IMiD	Pom/Dex, HDACi	SD	–	–	low
RRMM12	6	NA	–	NA	–	–	high
RRMM13	4	PI	Carfil/Dex	PR	–	–	int
RRMM14	3	IMiD	Pom/Dex	SD	–	–	low
RRMM15	3	IMiD	Pom/Dex, anti-SLAMF7	VGPR	–	–	high
RRMM16	3	MEKi	MEKi, BCL2i	CR	–	–	int
RRMM17	2	IMiD	Pom/Dex	PR	–	–	low
RRMM18	2	IMiD	Pom/Dex, Cyclo-phosphamid	SD	–	–	high
RRMM19	2	IMiD	Pom/Dex	PR	Carfil/Dex	VGPR	low
RRMM20	4	NA	–	NA	–	–	high

The RRMM patients studied here comprised 9 female and 11 male individuals with an average age of 61 years ranging from 43-68 years and a median of three prior lines treatment for 18/20 cases. Two patients were primary refractory to initial therapy (RRMM05 and RRMM07). For 15/20 patients paired samples before treatment and at relapse were acquired during either IMiD- (9), Carfilzomib- (3) or MEK inhibitor- (3) based therapies. A third time-point was analyzed after MCL1i- (RRMM06) and Carfilzomib- (RRMM19) based therapy, respectively. Treatment response was assessed by measuring monoclonal immunoglobulins and/or light chains in blood and urine in a central clinical lab.

[a] Treatment group. IMiD, immunmodulatory drugs; PI, proteasome inhibitor; MEKi, MEK inhibitor; MCL1i, MCL1 inhibitor; NA, not assessed.

[b] Pom, pomalidomide; Dex, dexamethasone, anti-CD38; Carfil, carfilzomib; BRAFi
[c] Best response. NA, not assessed; CR, complete response; VGPR, very good partial response; PR, partial response; MR, minimal response; SD, stable disease.
[d] Classification based on the InferCNV analysis into +1q low (<10%), intermediate (>10% and <80%) and high (>80%) according to the grouping shown in **Fig. 2J**.

Supplementary Table 2. Cell numbers of samples studied

Sample ID	Population	Cell number
RRMM01	PCs	1810
RRMM01	BME	4829
RRMM02	PCs	833
RRMM02	BME	2000
RRMM03	PCs	6227
RRMM03	BME	6927
RRMM04	PCs	839
RRMM04	BME	9395
RRMM05	PCs	10007
RRMM05	BME	11554
RRMM06	PCs	5277
RRMM06	BME	10925
RRMM07	PCs	1664
RRMM07	BME	6181
RRMM08	PCs	1027
RRMM08	BME	15116
RRMM09	PCs	2407
RRMM09	BME	3853
RRMM10	PCs	1932
RRMM10	BME	5575
RRMM11	PCs	3885
RRMM11	BME	3100
RRMM12	PCs	20193
RRMM12	BME	9644
RRMM13	PCs	932
RRMM13	BME	8663
RRMM14	PCs	1971
RRMM14	BME	4455
RRMM15	PCs	7309
RRMM15	BME	3049
RRMM16	PCs	2982
RRMM16	BME	5191
RRMM17	PCs	1112
RRMM17	BME	3462
RRMM18	PCs	6830
RRMM18	BME	11715
RRMM19	PCs	4644
RRMM19	BME	3569
RRMM20	PCs	1320
RRMM20	BME	not analyzed

Sample ID	Population	Cell number
BM1	PCs	118
BM1	BME	36129
BM2	PCs	425
BM2	BME	34224
BM3	PCs	275
BM3	BME	34101
BM4	PCs	312
BM4	BME	32780
BM5	PCs	185
BM5	BME	30917
BM6	PCs	288
BM6	BME	40684
BM7	PCs	547
BM7	BME	33176
BM8	PCs	181
BM8	BME	36819

Supplementary Table 3. Cell types and marker genes

Cell type	Abbreviation	Main marker gene(s) and features	Ref.
$CD4^+$ naive T-cell	T_CD4_naive	$CD3D^+$, $CD40LG^+$, $CCR7^+$, $LEF1^+$	[4-6]
$CD4^+$ memory T-cell	T_CD4_mem	$CD3D^+$, $CD40LG^+$, $IL7R^+$	[4-6]
Th17 T helper cell	Th17	$CD3D^+$, $CD40LG^+$, $KLRB1^+$	[4-6]
Regulatory T-cell	Treg	$CD3D^+$, $CD40LG^+$, $IL2RA^+$, $FOXP3^+$	[4-6]
Cycling T-cell	T_cycling	$CD3D^+$, $MKI67^+$, mostly S or G2/M phase	-
$CD8^+$ naïve T-cell	T_CD8_naive	$CD3D^+$, $CD40LG^+$, $CCR7^+$, $LEF1^+$	[4-6]
$CD8^+$ memory effector T-cell	T_CD8_mem	$CD3D^+$, $CD8A^+$, $GZMK^+$	[4-6]
$CD8^+$ high cytokine memory effector T-cell	T_CD8_ck	$CD3D^+$, $CD8A^+$, $CCL3^+$/$CCL4^+$, expressing high levels of cytokines IFNG, CCL3, or CCL4	[4-6]
$CD8^+$ cytotoxic T-cell	T_CD8_tox	$CD3D^+$, $CD8A^+$, $GZMH^+$	[4-6]
γδ T-cell	gdT	$CD3D^+$, $CD3E^+$, $TRDC^+$, $TRGC1^+$	[7]
NK T-cell	NKT	$CD3D^+$, CD56/$NCAM1^{dim}$, $FCGR3A^{dim}$, $KLRC2^+$	[8]
NK^{bright} cell	NK_bright	$FCER1G^+$, CD56/$NCAM1^+$, $FCGR3A^{dim}$,	[9]
NK^{dim} cell	NK_dim	$FCER1G^+$, CD56/$NCAM1^{dim}$, $CD16^+$/$FCGR3A^+$	[9]
NK^{dim} activated cell	NK_act	$CD69^+$ subgroup of NK^{dim}	This study
NK^{dim} effector cell	NK_eff	NK^{dim}; $GZMB^{high}$, $PRF1^{high}$, $CD16^+$/$FCGR3A^{high}$	This study
Common lymphoid progenitor	CLP	$IGLL1^+$, ADA^+	[10]
Pro B-cell	ProB	$IGLL1^+$, $VPREB1^+$	[10,11]
Pre B-cell	PreB	$IGLL1^+$, $CD24^+$,	[10,11]
Immature B-cell	B_im	$NEIL1^+$, $MS4A1^+$	[10,11]
Mature B-cell	B	$MS4A1^+$	[10,11]
Plasma cell	PC	$TNFRSF17^+$	[10,11]
Megakaryocyte progenitor	MkP	$PF4^+$	[10]
Erythroid progenitor cell	ERP	$SLC40A1^+$	[10]
Erythroblast	Er	$AHSP^+$	[10]
Hematopoietic stem cell	HSC	AVP^+	[10]
Multi-potent progenitor	MPP	$SPINK2^+$	[10]
Mast-cell precursor	prMa	$TPSB2^+$	[10]
Granulocyte-monocyte progenitor	GMP	$ELANE^+$	[10,12]
Monocyte precursor	prMono	$RETN^+$	[10,12]
$CD14^+$ monocyte	Mono_CD14	$CD14^+$	[10,12]
$CD16^+$ monocyte	Mono_CD16	$CD16^+$/$FCGR3A^+$	[10,12]
Intermediate monocyte	IM	$CD14^+$, $FCN1^+$, $VCAN^+$, $S100A8^+$, $VEGFA^+$	This study
Tumor-associated macrophage 1	TAM1	$CD68^+$, IFN-$response^+$, MHC I^{Hi}	This study
Tumor-associated macrophage 2	TAM2	$CD68^+$, $C1Q^+$, IFN-$response^+$, MHC I^{Hi}	This study
Tumor-associated macrophage 3	TAM3	$CD14^+$, $CD68^+$, $C1Q^+$, $MRC1^+$, $FOLR2^+$, $APOE^+$, MHC II^{HI}	This study
Dendritic cell precursor	preDC	$IGLL1^+$	[13,14]
Plasmacytoid dendritic cell	pDC	$CLEC4C^+$	[13,14]
Conventional dendritic cell 1	cDC1	$CLEC9A^+$	[13,14]
Conventional dendritic cell 2	cDC2	$CLEC10A^+$	[13,14]

Supplementary Table 4. Gene expression signatures

Signature	Genes	Ref.
T-cell dysfunction/ exhaustion	PDCD1, CTLA4, TIGIT, LAG3, HAVCR2, CD244, VSIR	[5,8,15]
Effectorness	PRF1, GZMA, GNLY, GZMH, GZMK, NKG7	[4-6]
IFNG-response	HALLMARK IFNG-response	[16]
Ribosome	KEGG-Ribosome	[17]
TNFα-signaling	HALLMARK_TNFA_SIGNALING_VIA_NFKB	[16]
IL1-signaling	PID_IL1_PATHWAY	[17]
IL2-signaling	HALLMARK_IL2_STAT5_SIGNALING	[16]
IL6-signaling	HALLMARK_IL6_JAK_STAT3_SIGNALING	[16]
Inflammatory response	HALLMARK_INFLAMMATORY_RESPONSE	[16]
TGFβ-signaling	HALLMARK_TGF_BETA-SIGNALING	[16]
KLF6 target genes	ASAH1, ATF3, CDH1, CDKN1A, CERS2, CGB5, DAPK2, IGF1R, KRT12, LAMA1, LTC4S, NOS2, PMAIP1, PSG3, PSG5, TFPI2, TXNIP)	[18]
1q gain (+1q) [a]	PMVK, ILF2, CCT3, TIMM17A, LAMTOR2, SNRPE, TMCO1, PSMB4, PSMD4, COX20, HAX1, KRTCAP2, EPRS, PARP1, SDHC, AC245014.3, NAXE, UFC1, ARPC5, RBM8A, H3F3A, IFI16, TAGLN2, MRPL55, ARF1, MRPS21, DPM3, ADAR, C1orf43, RGS1, UAP1, MPC2, GUK1, TSTD1, NENF, MGST3, SSR2, PFDN2, JTB, HIST2H2BE, HNRNPU, BTG2, MDM4, B4GALT3, TOMM20, CTSS, LMNA, RPS27, RGS2, SLAMF7, MCL1	This study

[a] The +1q gene expression signature comprised 51 genes and included known drivers of multiple myeloma pathogenesis that have already been linked to +1q such as *ILF2* [19], *ADAR* [20] and again *MCL1* [21]. Furthermore, we detected several genes that so far have not been associated with +1q multiple myeloma: *SLAMF7,* a primary drug target in multiple myeloma [22]; *PARP1* (encoding for poly[ADP-ribose] polymerase), an enzyme involved in DNA repair and promising drug target in BRCA-defective tumors [23]; the regulator of G-protein signaling *RGS1* whose expression is associated with poor prognosis in multiple myeloma [24]; and *CTSS* (Cathepsin S), a cysteine protease involved in the recruitment of immunosuppressive myeloid cells [25,26]

Supplementary Table 5. Data analysis software

Software	Ref.	Link
ACEseq	27	https://aceseq.readthedocs.io/
Bowtie	28	bowtie-bio.sourceforge.net/bowtie2/index.shtml
Bioconductor R[a]	29	www.bioconductor.org
CellPhoneDB	30	https://www.cellphonedb.org
ComplexHeatmap	31	https://www.bioconductor.org/packages/release/bioc/html/ComplexHeatmap.html
Custom scripts and pipelines for this study	32	https://github.com/RippeLab/RRMM
Cytoscape	33	cytoscape.org
DAVID	34	david.ncifcrf.gov
dendextend	35	https://github.com/talgalili/dendextend
DESeq2	1	doi.org/10.18129/B9.bioc.DESeq2
EnhancedVolcano	36	github.com/kevinblighe/EnhancedVolcano
ggpointdensity		github.com/LKremer/ggpointdensity
Harmony	37	https://github.com/immunogenomics/harmony
hypeR	38	github.com/montilab/hypeR
inferCNV	39	github.com/broadinstitute/inferCNV
Nextflow[b]	40	https://github.com/nextflow-io/nextflow
nf-core (bulk RNA-seq)	41	https://nf-co.re, https://github.com/nf-core/rnaseq
OTP WGS pipeline	42	https://otp.dkfz.de/otp/
presto		https://github.com/immunogenomics/presto
schex		github.com/SaskiaFreytag/schex
Scrublet	43	https://github.com/AllonKleinLab/scrublet
SCTransform	44	https://github.com/ChristophH/sctransform
Seurat	45	https://satijalab.org/seurat/
SingleR	46	https://github.com/LTLA/SingleR
STAR	47	github.com/alexdobin/STAR
UMAP	48	https://github.com/lmcinnes/umap/archive/0.2.4.tar.gz

[a] Visualization of data was conducted with the R packages ggplot2 and igraph.

[b] Initial quality control including removal of low-quality libraries, doublet detection and celltype prediction with SingleR, single cell copy number calling (InferCNV) and prediction of cellular interactions (CellphoneDB) were implemented in Nextflow for automated processing of data sets.

Supplementary Table 6. Antibodies for fluorescence activated cell sorting

Antibody target	Fluoro-chrome	Clone	Dilution Factor	Order number	Company
CD56	APC	NCAM16.2	1:40	341025	Becton Dickinson
CD16	FITC	3G8	1:10	560996	Becton Dickinson
CD38	PE-CF594	HIT2	1:40	562288	Becton Dickinson
CD3	BV650	SK7	1:40	563999	Becton Dickinson
CD3	APC-R700	UCHT1	1:75	565119	Becton Dickinson
LAG-3	PE	T47-530	1:30	565616	Becton Dickinson
CD138	BV421	MI15	1:40	565943	Becton Dickinson
PD-1	BV480	EH12.1	1:30	566112	Becton Dickinson)
GranzymeB	R718	GB11	1:40	566964	Becton Dickinson
CD45	BUV805	HI30	1:40	612891	Becton Dickinson
CD45	APC-H7	2D1	1:50	641417	Becton Dickinson
TCRγ/δ	PE-Cy7	11F2	1:30	655410	Becton Dickinson
CD14	BUV563	M5E2	1:40	741360	Becton Dickinson
TIGIT	BV421	741182	1:30	747844	Becton Dickinson
CD8	APC	RPA-T8	1:30	555369	Becton Dickinson
CD159a	BB700	131411	1:40	747926	Becton Dickinson
KLRG-1	BV605	2F1/KLRG1	1:30	138419	Biolegend
CD11b	PE-Cy5	ICRF44	1:40	301308	Biolegend
CD218a	PE-Cy7	H44	1:40	313812	Biolegend
CD206	PE	15-2	1:40	321105	Biolegend
CD163	BV711	GHI/61	1:40	333630	Biolegend
IL18	Alexa Fluor 750	74801	1:20	IC646S-100µg	R&D Systems

Supplementary Table 7. Inventory of supplementary data sets

**Data set**	**File name**	**Figure/ Table ref.**	**Description**
Supplementary Data Set 1	Sup_Data_01.xlsx	Fig. 1, Supplementary Fig. 1, Supplementary Table 1	Sample meta data with sample IDs and additional sample information.
Supplementary Data Set 2	Sup_Data_02.xlsx	Fig. 2, Supplementary Fig. 2, Supplementary Table 4	Annotation of upregulated genes of the +1q signature

These data sets are provided as separate files in Microsoft Excel format.

References

1. Love, M.I., Huber, W. & Anders, S. Moderated estimation of fold change and dispersion for RNA-seq data with DESeq2. Genome Biol 15, 550 (2014).
2. Seckinger, A. et al. Target Expression, Generation, Preclinical Activity, and Pharmacokinetics of the BCMA-T Cell Bispecific Antibody EM801 for Multiple Myeloma Treatment. Cancer Cell 31, 396-410 (2017).
3. Ledergor, G. et al. Single cell dissection of plasma cell heterogeneity in symptomatic and asymptomatic myeloma. Nat Med 24, 1867-1876 (2018).
4. Park, J.E. et al. A cell atlas of human thymic development defines T cell repertoire formation. Science 367(2020).
5. Szabo, P.A. et al. Single-cell transcriptomics of human T cells reveals tissue and activation signatures in health and disease. Nat Commun 10, 4706 (2019).
6. van der Leun, A.M., Thommen, D.S. & Schumacher, T.N. CD8(+) T cell states in human cancer: insights from single-cell analysis. Nat Rev Cancer 20, 218-232 (2020).
7. Pizzolato, G. et al. Single-cell RNA sequencing unveils the shared and the distinct cytotoxic hallmarks of human TCRVdelta1 and TCRVdelta2 gammadelta T lymphocytes. Proc Natl Acad Sci U S A 116, 11906-11915 (2019).
8. Johnston, R.J. et al. VISTA is an acidic pH-selective ligand for PSGL-1. Nature 574, 565-570 (2019).
9. Stabile, H., Fionda, C., Gismondi, A. & Santoni, A. Role of Distinct Natural Killer Cell Subsets in Anticancer Response. Front Immunol 8, 293 (2017).
10. Hay, S.B., Ferchen, K., Chetal, K., Grimes, H.L. & Salomonis, N. The Human Cell Atlas bone marrow single-cell interactive web portal. Exp Hematol 68, 51-61 (2018).
11. Bendall, S.C. et al. Single-cell trajectory detection uncovers progression and regulatory coordination in human B cell development. Cell 157, 714-25 (2014).
12. van Galen, P. et al. Single-Cell RNA-Seq Reveals AML Hierarchies Relevant to Disease Progression and Immunity. Cell 176, 1265-1281 e24 (2019).
13. Collin, M. & Bigley, V. Human dendritic cell subsets: an update. Immunology 154, 3-20 (2018).
14. Merad, M., Sathe, P., Helft, J., Miller, J. & Mortha, A. The dendritic cell lineage: ontogeny and function of dendritic cells and their subsets in the steady state and the inflamed setting. Annu Rev Immunol 31, 563-604 (2013).
15. Li, H. et al. Dysfunctional CD8 T Cells Form a Proliferative, Dynamically Regulated Compartment within Human Melanoma. Cell 176, 775-789 e18 (2019).
16. Liberzon, A. et al. The Molecular Signatures Database (MSigDB) hallmark gene set collection. Cell Syst 1, 417-425 (2015).
17. Subramanian, A. et al. Gene set enrichment analysis: a knowledge-based approach for interpreting genome-wide expression profiles. Proc Natl Acad Sci U S A 102, 15545-50 (2005).
18. Han, H. et al. TRRUST v2: an expanded reference database of human and mouse transcriptional regulatory interactions. Nucleic Acids Res 46, D380-D386 (2018).
19. Samo, A.A. et al. MCL1 gene co-expression module stratifies multiple myeloma and predicts response to proteasome inhibitor-based therapy. Genes Chromosomes Cancer 57, 420-429 (2018).
20. Lazzari, E. et al. Alu-dependent RNA editing of GLI1 promotes malignant regeneration in multiple myeloma. Nat Commun 8, 1922 (2017).
21. Slomp, A. et al. Multiple myeloma with 1q21 amplification is highly sensitive to MCL-1 targeting. Blood Adv 3, 4202-4214 (2019).
22. van de Donk, N.W. et al. Clinical efficacy and management of monoclonal antibodies targeting CD38 and SLAMF7 in multiple myeloma. Blood 127, 681-95 (2016).
23. Rouleau, M., Patel, A., Hendzel, M.J., Kaufmann, S.H. & Poirier, G.G. PARP inhibition: PARP1 and beyond. Nat Rev Cancer 10, 293-301 (2010).

24. Roh, J. et al. RGS1 expression is associated with poor prognosis in multiple myeloma. J Clin Pathol 70, 202-207 (2017).
25. Yang, M. et al. Cathepsin S-mediated autophagic flux in tumor-associated macrophages accelerate tumor development by promoting M2 polarization. Mol Cancer 13, 43 (2014).
26. Wilkinson, R.D. et al. CCL2 is transcriptionally controlled by the lysosomal protease cathepsin S in a CD74-dependent manner. Oncotarget 6, 29725-39 (2015).
27. Giessler, K.M. et al. Genetic subclone architecture of tumor clone-initiating cells in colorectal cancer. J Exp Med 214, 2073-2088 (2017).
28. Langmead, B., Trapnell, C., Pop, M. & Salzberg, S.L. Ultrafast and memory-efficient alignment of short DNA sequences to the human genome. Genome Biol 10, R25 (2009).
29. Gentleman, R.C. et al. Bioconductor: open software development for computational biology and bioinformatics. Genome Biol 5, R80 (2004).
30. Efremova, M., Vento-Tormo, M., Teichmann, S.A. & Vento-Tormo, R. CellPhoneDB: inferring cell-cell communication from combined expression of multi-subunit ligand-receptor complexes. Nat Protoc 15, 1484-1506 (2020).
31. Gu, Z., Eils, R. & Schlesner, M. Complex heatmaps reveal patterns and correlations in multidimensional genomic data. Bioinformatics 32, 2847-9 (2016).
32. Steiger, S., Tirier, S.M. & Rippe, K. Nextflow data processing pipelines and R code for single cell RNA sequencing analysis of relapsed and refractory multiple myeloma. Zenodo, https://doi.org/10.5281/zenodo.5532552 (2021).
33. Shannon, P. et al. Cytoscape: a software environment for integrated models of biomolecular interaction networks. Genome Res 13, 2498-504 (2003).
34. Huang da, W., Sherman, B.T. & Lempicki, R.A. Systematic and integrative analysis of large gene lists using DAVID bioinformatics resources. Nat Protoc 4, 44-57 (2009).
35. Galili, T. dendextend: an R package for visualizing, adjusting and comparing trees of hierarchical clustering. Bioinformatics 31, 3718-20 (2015).
36. Blighe, K., Rana, S. & Lewis, M. EnhancedVolcano: Publication-ready volcano plots with enhanced colouring and labeling. Github, https://github.com/kevinblighe/EnhancedVolcano (2018).
37. Korsunsky, I. et al. Fast, sensitive and accurate integration of single-cell data with Harmony. Nat Methods 16, 1289-1296 (2019).
38. Federico, A. & Monti, S. hypeR: an R package for geneset enrichment workflows. Bioinformatics 36, 1307-1308 (2020).
39. Tickle, T., Tirosh, I., Georgescu, C., Brown, M. & Haas, B. inferCNV of the Trinity CTAT Project. Github, https://github.com/broadinstitute/inferCNV. (2019).
40. Di Tommaso, P. et al. Nextflow enables reproducible computational workflows. Nat Biotechnol 35, 316-319 (2017).
41. Ewels, P.A. et al. The nf-core framework for community-curated bioinformatics pipelines. Nat Biotechnol 38, 276-278 (2020).
42. Reisinger, E. et al. OTP: An automatized system for managing and processing NGS data. J Biotechnol 261, 53-62 (2017).
43. Wolock, S.L., Lopez, R. & Klein, A.M. Scrublet: Computational Identification of Cell Doublets in Single-Cell Transcriptomic Data. Cell Syst 8, 281-291 e9 (2019).
44. Hafemeister, C. & Satija, R. Normalization and variance stabilization of single-cell RNA-seq data using regularized negative binomial regression. Genome Biol 20, 296 (2019).
45. Stuart, T. et al. Comprehensive Integration of Single-Cell Data. Cell 177, 1888-1902 e21 (2019).
46. Aran, D. et al. Reference-based analysis of lung single-cell sequencing reveals a transitional profibrotic macrophage. Nat Immunol 20, 163-172 (2019).
47. Dobin, A. et al. STAR: ultrafast universal RNA-seq aligner. Bioinformatics 29, 15-21 (2013).
48. Becht, E. et al. Dimensionality reduction for visualizing single-cell data using UMAP. Nat Biotechnol 37, 38-44 (2019).
