# Supplementary material for: Subclone-specific microenvironmental impact and drug response in refractory multiple myeloma revealed by single‐cell transcriptomics
Source: Nat Commun. 2021 Nov 29;12:6960. doi: 10.1038/s41467-021-26951-z (PMC8630108; doi:10.1038/s41467-021-26951-z)
Supplement: Supplementary file 5 — Reporting summary. [file 41467_2021_26951_MOESM5_ESM.pdf]

nature research

Corresponding author(s): Karsten Rippe, Marc Raab

Last updated by author(s): Sep 30, 2021

Reporting Summary

Nature Research wishes to improve the reproducibility of the work that we publish. This form provides structure for consistency and transparency in reporting. For further information on Nature Research policies, see our Editorial Policies and the Editorial Policy Checklist.

Statistics

For all statistical analyses, confirm that the following items are present in the figure legend, table legend, main text, or Methods section.

n/a	Confirmed	
☐	☒	The exact sample size (*n*) for each experimental group/condition, given as a discrete number and unit of measurement
☐	☒	A statement on whether measurements were taken from distinct samples or whether the same sample was measured repeatedly
☐	☒	The statistical test(s) used AND whether they are one- or two-sided<br>*Only common tests should be described solely by name; describe more complex techniques in the Methods section.*
☐	☒	A description of all covariates tested
☐	☒	A description of any assumptions or corrections, such as tests of normality and adjustment for multiple comparisons
☐	☒	A full description of the statistical parameters including central tendency (e.g. means) or other basic estimates (e.g. regression coefficient) AND variation (e.g. standard deviation) or associated estimates of uncertainty (e.g. confidence intervals)
☐	☒	For null hypothesis testing, the test statistic (e.g. *F*, *t*, *r*) with confidence intervals, effect sizes, degrees of freedom and *P* value noted<br>*Give P values as exact values whenever suitable.*
☒	☐	For Bayesian analysis, information on the choice of priors and Markov chain Monte Carlo settings
☒	☐	For hierarchical and complex designs, identification of the appropriate level for tests and full reporting of outcomes
☐	☒	Estimates of effect sizes (e.g. Cohen's *d*, Pearson's *r*), indicating how they were calculated

Our web collection on statistics for biologists contains articles on many of the points above.

Software and code

Policy information about availability of computer code

Data collection

Single cell RNA libraries were generated with the Chromium single cell 3' v2 chemistry (10x Genomics). Sequencing was done on the Illumina HiSeq 4000 platform. Demultiplexing and alignment of sequencing reads were conducted with the CellRanger v3.0.1 software (10x Genomics)

Data analysis

Custom analysis scripts and tools are available from Github at https://github.com/RippeLab/RRMM or from Zenodo at https://doi.org/10.5281/zenodo.5532552. Other software used is listed in Supplementary Table 5 of the manuscript and comprises the following:

ACEseq https://aceseq.readthedocs.io/
Bowtie bowtie-bio.sourceforge.net/bowtie2/index.shtml
Bioconductor R www.bioconductor.org
CellPhoneDB https://www.cellphonedb.org
ComplexHeatmap https://www.bioconductor.org/packages/release/bioc/html/ ComplexHeatmap.html
Cytoscape cytoscape.org
DAVID david.ncifcrf.gov
dendextend https://github.com/talgalili/dendextend
DESeq2 doi.org/10.18129/B9.bioc.DESeq2
EnhancedVolcano github.com/kevinblighe/EnhancedVolcano
ggpointdensity github.com/LKremer/ggpointdensity
Harmony https://github.com/immunogenomics/harmony
hypeR github.com/montilab/hypeR
inferCNV github.com/broadinstitute/inferCNV
Nextflow https://github.com/nextflow-io/nextflow
nf-core (bulk RNA-seq) https://nf-co.re, https://github.com/nf-core/rnaseq
OTP WGS pipeline https://otp.dkfz.de/otp/
presto https://github.com/immunogenomics/presto

schex github.com/SaskiaFreytag/schex
Scrublet https://github.com/AllonKleinLab/scrublet
SCTransform https://github.com/ChristophH/sctransform
Seurat https://satijalab.org/seurat/
SingleR https://github.com/LTLA/SingleR
STAR github.com/alexdobin/STAR
UMAP https://github.com/lmcinnes/umap/archive/0.2.4.tar.gz

For manuscripts utilizing custom algorithms or software that are central to the research but not yet described in published literature, software must be made available to editors and reviewers. We strongly encourage code deposition in a community repository (e.g. GitHub). See the Nature Research guidelines for submitting code & software for further information.

Data

Policy information about availability of data

All manuscripts must include a data availability statement. This statement should provide the following information, where applicable:

- Accession codes, unique identifiers, or web links for publicly available datasets
- A list of figures that have associated raw data
- A description of any restrictions on data availability

The original sequencing data generated in this study have been deposited in the European Genome-phenome Archive under the accession number EGAS00001004805 (https://ega-archive.org/studies/EGAS00001004805). These data are available under restricted access to comply with German and European data protection regulations, and can be obtained upon application to the linked data access committee. The processed scRNA-seq data used in this study are publicly available at Gene Expression Omnibus under accession number GSE161801 (https://www.ncbi.nlm.nih.gov/geo/query/acc.cgi?acc=GSE161801). The scRNA-seq data of healthy bone marrow donors (census of immune cells) were from the Human Cell Atlas database (https://data.humancellatlas.org/explore/projects/cc95ff89-2e68-4a08-a234-480eca21ce79). Additional data are provided as Supplementary Data Sets (Supplementary Table 7). Source data are provided with this paper.

Field-specific reporting

Please select the one below that is the best fit for your research. If you are not sure, read the appropriate sections before making your selection.

☒ Life sciences ☐ Behavioural & social sciences ☐ Ecological, evolutionary & environmental sciences

For a reference copy of the document with all sections, see nature.com/documents/nr-reporting-summary-flat.pdf

Life sciences study design

All studies must disclose on these points even when the disclosure is negative.

Sample size	No statistical test was used to predetermine sample size. Clinical samples with sufficiently high numbers of viable cells were used. We profiled 20 patients with at least 500 cells per sample to cover a wide range of myeloma subtypes and immune cell types (Supplementary Table 2). The sample size was sufficiently high to identify statistically significant changes in cell type composition and gene expression.
Data exclusions	Removal of immunoglobulin genes (n = 410)<br>Quality filtering, removal of cells with mitochondrial transcript >10% and number of detected genes per cell < 400.<br>Doublet removal with Scrublet Python tool, sim_doublet_ratio = 2; n_neighbors = 30; expected_doublet_rate = 0.1. All cells with a doublet score > 0.4 were discarded as described in the manuscript.<br>Removal of low-quality clusters that had a high percentages of mitochondrial gene counts, low housekeeping signature scores and/or low numbers of detected genes as well as no expression of biologically relevant cell type or cell state specific genes.
Replication	Successful validation experiments were carried out using both fresh/frozen + sorted/non-sorted samples of the same patient for three different patients (Supplementary Figure 1).
Randomization	No randomization was used. This study of patient data is observational.
Blinding	Investigators were not blinded during data collection and analysis.

Reporting for specific materials, systems and methods

We require information from authors about some types of materials, experimental systems and methods used in many studies. Here, indicate whether each material, system or method listed is relevant to your study. If you are not sure if a list item applies to your research, read the appropriate section before selecting a response.

Materials & experimental systems

n/a	Involved in the study
☐	☒ Antibodies
☒	☐ Eukaryotic cell lines
☒	☐ Palaeontology and archaeology
☒	☐ Animals and other organisms
☐	☒ Human research participants
☒	☐ Clinical data
☒	☐ Dual use research of concern

Methods

n/a	Involved in the study
☒	☐ ChIP-seq
☐	☒ Flow cytometry
☒	☐ MRI-based neuroimaging

Antibodies

Antibodies used

All antibodies and dilution factors used in this study are listed in Supplementary Table 6.

Validation

All antibodies used in this study are commercially available with validation procedures described on the following sites of the manufacturers:

CD56 (BD)
https://www.bdbiosciences.com/content/dam/bdb/products/global/reagents/flow-cytometry-reagents/clinical-diagnostics/single-color-antibodies-asr-ivd-ce-ivd/341027_base/pdf/23-5109.pdf
CD56 was submitted to the Fourth International Workshop and Conference on Human Leucocyte Differentiation Antigens. Participating laboratories evaluated clone NCAM16.2 as part of a blind panel of antibodies and reported consistent results

CD16 (BD)
https://www.bdbiosciences.com/en-eu/products/reagents/flow-cytometry-reagents/research-reagents/single-color-antibodies-ruo/fitc-mouse-anti-human-cd16.560996
Flow cytometry was tested on Rhesus macaque peripheral blood lymphocytes by the manufacturer.

CD38 (BD)
https://www.bdbiosciences.com/en-eu/products/reagents/flow-cytometry-reagents/research-reagents/single-color-antibodies-ruo/pe-cf594-mouse-anti-human-cd38.562288
Flow cytometry was tested on human peripheral blood lymphocytes by the manufacturer.

CD3 (BD) clone SK7
https://www.bdbiosciences.com/en-eu/products/reagents/flow-cytometry-reagents/research-reagents/single-color-antibodies-ruo/bv650-mouse-anti-human-cd3.563999
Flow cytometry was tested on human peripheral blood lymphocytes by the manufacturer.

CD3 (BD) clone UCHT1
https://www.bdbiosciences.com/en-eu/products/reagents/flow-cytometry-reagents/research-reagents/single-color-antibodies-ruo/apc-r700-mouse-anti-human-cd3.565119
Flow cytometry was tested on human peripheral blood lymphocytes by the manufacturer.

LAG-3 (BD)
https://www.bdbiosciences.com/en-eu/products/reagents/flow-cytometry-reagents/research-reagents/single-color-antibodies-ruo/pe-mouse-anti-human-lag-3-cd223.565616
Flow cytometry was tested on activated human peripheral blood lymphocytes by the manufacturer.

CD138 (BD)
https://www.bdbiosciences.com/en-eu/products/reagents/flow-cytometry-reagents/research-reagents/single-color-antibodies-ruo/bv421-mouse-anti-human-cd138.562935
Flow cytometry was tested on U266 cells by the manufacturer.

PD-1 (BD)
https://www.bdbiosciences.com/en-eu/products/reagents/flow-cytometry-reagents/research-reagents/single-color-antibodies-ruo/bv480-mouse-anti-human-cd279-pd-1.566112
Flow cytometry was tested on human peripheral blood lymphocytes by the manufacturer.

GranzymB (BD)
https://www.bdbiosciences.com/en-eu/products/reagents/flow-cytometry-reagents/research-reagents/single-color-antibodies-ruo/r718-mouse-anti-human-granzyme-b.566964
Flow cytometry was tested on peripheral blood lymphocytes by the manufacturer.

CD45 (BD) clone HI30
https://www.bdbiosciences.com/en-eu/products/reagents/flow-cytometry-reagents/research-reagents/single-color-antibodies-ruo/buv805-mouse-anti-human-cd45.612891
Flow cytometry was tested on human peripheral blood lymphocytes by the manufacturer.

CD45 (BD) clone 2D1
https://www.bdbiosciences.com/en-eu/products/reagents/flow-cytometry-reagents/clinical-diagnostics/single-color-antibodies-asr-

ivd-ce-ivd/cd45-apc-h7.641417
The antibody was submitted to the Fourth International Workshop and Conference on Human Leucocyte Differentiation Antigens. Participating laboratories evaluated clone 2D1 as part of a blind panel of antibodies and reported consistent results

TCRγ/δ (BD)
https://www.bdbiosciences.com/en-eu/products/reagents/flow-cytometry-reagents/clinical-discovery-research/single-color-antibodies-ruo-gmp/pe-cy-7-mouse-anti-human-tcr.655410
Optimized for flow cytometry by the manufacturer. Cited in 16 studies.

CD14 (BD)
https://www.bdbiosciences.com/content/bdb/paths/generate-tds-document.eu.741360.pdf
Optimized for flow cytometry by the manufacturer

TIGIT (BD)
https://www.bdbiosciences.com/en-eu/products/reagents/flow-cytometry-reagents/research-reagents/single-color-antibodies-ruo/bv421-mouse-anti-human-tigit.747844
Flow cytometry was tested on peripheral blood lymphocytes by the manufacturer.

CD8 (BD)
https://www.bdbiosciences.com/en-eu/products/reagents/flow-cytometry-reagents/research-reagents/single-color-antibodies-ruo/apc-mouse-anti-human-cd8.555369
Flow cytometry was tested on human peripheral blood lymphocytes by the manufacturer.

CD159a (NKG2A) (BD)
https://www.bdbiosciences.com/content/bdb/paths/generate-tds-document.eu.747926.pdf
Optimized for flow cytometry by the manufacturer

KLRG-1 (Biolegend)
https://www.biolegend.com/en-us/products/brilliant-violet-605-anti-mouse-human-klrg1-mafa-antibody-9644
Flow cytometry was tested on mouse splenocytes (Reactivity both mouse and human)

CD11b (Biolegend)
https://www.biolegend.com/en-us/products/pe-cyanine5-anti-human-cd11b-antibody-769
Flow cytometry was tested on human peripheral blood lymphocytes, monocytes and granulocytes by the manufacturer.

CD218a (Biolegend)
https://www.biolegend.com/en-us/search-results/pe-cyanine7-anti-human-cd218a-il-18ralpha-antibody-17477
Flow cytometry was tested on human peripheral blood lymphocytes by the manufacturer.

CD206 (Biolegend)
https://www.biolegend.com/en-us/search-results/pe-anti-human-cd206-mmr-antibody-2994
Flow cytometry was tested on human monocytes by the manufacturer.

CD163 (Biolegend)
https://www.biolegend.com/en-us/products/brilliant-violet-711-anti-human-cd163-antibody-14298
Flow cytometry was tested on human peripheral blood monocytes by the manufacturer.

IL18 (R&D Systems)
https://www.rndsystems.com/products/human-il-18-il-1f4-propeptide-alexa-fluor-750-conjugated-antibody-74801_ic646s
Optimized for flow cytometry by the manufacturer

Additional information by the manufactures can be found here:
- Biolegend: https://www.biolegend.com/en-us/quality-control
- BD: https://www.bdbiosciences.com/us/go/reproducibility
- R&D systems: https://www.rndsystems.com/quality/antibodies-built-for-reproducibility

Human research participants

Policy information about studies involving human research participants

Population characteristics

Relapsed/refractory multipe myeloma. Male and female patients were between 43-77 years of age with an average of 61 years. All patients were refractory to their immediate prior line of treatment. A number of 18 patients were refractory to both a proteasome inhibitor and an immunomodulatory drug, while 2 patients were primarily refractory to initial therapy. Paired samples before treatment and at relapse were analyzed.

Recruitment

Patient were included into the data registry and biorepository on a rolling basis. Samples were retrieved from the repository based on disease status of the individual patient and available cell numbers. All patients provided written informed consent to the data registry and biorepository. With respect to the previous treatments and the cytogenetic characteristics of the patients we see no evidence for a self-selection bias. However, the obtained cell numbers were a criterium for sample selection, which might reflect intrinsic characteristics of the patients disease phenotype. For example, a high grade of inflammation or necrosis in the bone marrow might affect the number of cells obtained from a given patient sample.

Ethics oversight

All patients provided written informed consent before participating in the study. Approval was obtained by the ethics committee of the University of Heidelberg.

Note that full information on the approval of the study protocol must also be provided in the manuscript.

Flow Cytometry

Plots

Confirm that:

☒ The axis labels state the marker and fluorochrome used (e.g. CD4-FITC).

☒ The axis scales are clearly visible. Include numbers along axes only for bottom left plot of group (a 'group' is an analysis of identical markers).

☒ All plots are contour plots with outliers or pseudocolor plots.

☒ A numerical value for number of cells or percentage (with statistics) is provided.

Methodology

Sample preparation	T-cells: Cells were suspended in staining buffer that comprised phosphate-buffered saline (PBS) supplemented with 0.5% BSA, incubated according to the manufacturer's instructions with fluorochrome-labeled antibodies for 30 min at 4 °C, and then washed two times.<br>TAM/NK: Intracellular staining was performed using a transcription factor buffer set (BD Biosciences, Heidelberg, Germany) that comprised Fix, Perm and Wash buffer according to the manufacturer's instructions. Briefly, cells were resuspended in PBS and incubated for 15 minutes at 4 °C with fluorochrome-labeled monoclonal surface antibodies. After washing with 2 ml of PBS, cells were permeabilized with Fix/Perm buffer for 45 minutes at 4 °C, washed two times with Perm/Wash buffer, resuspended in an adequate amount of Perm/Wash buffer, and incubated with intracellular antibodies for 45 minutes at 4 ° C. Subsequently, cells were washed two times with Perm/Wash buffer and resuspended in 0.5 % paraformaldehyde in PBS.
Instrument	For T-cells: BD FACS Lyric flow cytometer<br>For TAM/NK: BD FACS Symphony flow cytometer
Software	FlowJo v10.7.1 software (BD)
Cell population abundance	Flow cytometry was performed for analytical purposes without sorting.
Gating strategy	Acquired events were gated according to their position on the FSC/SSC plot. Doublets were excluded based on their position on the FSC-A/FSC-H axes. Live cells were gated using live/dead staining (Fixable Viability Dye eFluor 506), and dead cells were excluded. Examples of gating strategies are shown in Supplementary Fig. 7 (T-cells) and Supplementary Fig. 9 (TAM/NK).

☒ Tick this box to confirm that a figure exemplifying the gating strategy is provided in the Supplementary Information.
