# Supplementary material for: Subclone-specific microenvironmental impact and drug response in refractory multiple myeloma revealed by single‐cell transcriptomics
Source: Nat Commun. 2021 Nov 29;12:6960. doi: 10.1038/s41467-021-26951-z (PMC8630108; doi:10.1038/s41467-021-26951-z)
Supplement: Supplementary file 6 — Manuscript and SI with changes marked. [file 41467_2021_26951_MOESM6_ESM.pdf]

Subclone-specific microenvironmental impact and drug response in refractory multiple myeloma revealed by single cell transcriptomics

Stephan M. Tirier[1], Jan-Philipp Mallm[1,2,3], Simon Steiger[1], Alexandra M. Poos[4,5], Mohamed H. S. Awwad[4], Nicola Giesen[4,5], Nicola Casiraghi[6,7], Hana Susak[6,7], Katharina Bauer[2,3], Anja Baumann[5], Lukas John[4,5], Anja Seckinger[4], Dirk Hose[4,8], Carsten Müller-Tidow[4], Hartmut Goldschmidt[4,9], Oliver Stegle[6,7], Michael Hundemer[4], Niels Weinhold[4,5], Marc S. Raab[4,5*], Karsten Rippe[1,*]

[1] Division of Chromatin Networks, German Cancer Research Center (DKFZ) and Bioquant, Heidelberg, Germany

[2] Single Cell Open Lab, German Cancer Research Center (DKFZ) and Bioquant, Heidelberg, Germany

[3] Molecular Precision Oncology Program, NCT Heidelberg, Heidelberg, Germany

[4] University Hospital Heidelberg, Internal Medicine V, Heidelberg, Germany

[5] CCU Molecular Hematology/Oncology, German Cancer Research Center (DKFZ), Heidelberg, Germany

[6] Division of Computational Genomics and System Genetics, German Cancer Research Center (DKFZ), Heidelberg, Germany

[7] Genome Biology Unit, European Molecular Biology Laboratory, Heidelberg, Germany

[8] Department of Hematology and Immunology, Myeloma Center Brussels, Jette, Belgium

[9] National Center for Tumor Diseases (NCT), Heidelberg, Germany

* Equally contributing authors, send correspondence to karsten.rippe@dkfz.de or marc.raab@med.uni-heidelberg.de

Keywords: single cell RNA sequencing; haematological malignancies; intratumor heterogeneity; cancer microenvironment; cellular interactions; bone marrow

Abstract

Virtually all patients with multiple myeloma become unresponsive to treatment over time. Relapsed/refractory multiple myeloma (RRMM) is accompanied by the clonal evolution of myeloma cells with heterogeneous genomic aberrations and profound changes of the bone marrow microenvironment (BME). However, the molecular mechanisms that drive drug resistance remain elusive. Here, we analyze the heterogeneous tumor cell population and its complex interaction network with the BME of 20 RRMM patients by single cell RNA-sequencing before/after treatment. Subclones with chromosome 1q-gain express a specific transcriptomic signature and frequently expand during treatment. Furthermore, RRMM cells shape an immune suppressive BME by upregulation of inflammatory cytokines and close interaction with the myeloid compartment. It is characterized by the accumulation of $PD1^{+}$ γδ T-cells and tumor-associated macrophages as well as the depletion of hematopoietic progenitors. Thus, our study resolves transcriptional features of subclones in RRMM and mechanisms of microenvironmental reprogramming with implications for clinical decision-making.

Introduction

Multiple myeloma (MM) is a hematological malignancy with clonal expansion of malignant plasma cells in the bone marrow [1]. The current treatment of MM with immunomodulatory drugs (IMiDs), proteasome inhibitors and monoclonal antibodies elicits deep remissions in patients with newly diagnosed MM [2]. However, almost all patients relapse and enter the relapsed/refractory multiple myeloma (RRMM) stage at some point [3]. This disease course is tightly linked to the remarkable genomic complexity of MM that becomes even more pronounced in RRMM and manifests itself as a widespread presence of multiple subclones [4, 5]. Furthermore, a number of studies have shown that alterations of non-malignant cells in the bone marrow microenvironment (BME) are critical for the pathogenesis of MM [6, 7, 8, 9, 10]. Thus, there is an urgent need to resolve tumor heterogeneity and changes of the BME to reveal molecular patient-specific characteristics that can guide treatment decisions, including emerging T-cell-based immunotherapies. Single cell RNA sequencing (scRNA-seq) is ideally suited to dissect tumor cell heterogeneity and its microenvironment as shown for MM [11] and cell types in the BME of MM patients [9]. However, an integrated scRNA-seq analysis of both tumor cells and BME from the same patient sample as well as the application to drug treatment response and RRMM cases are currently lacking.

In this work, we resolve cellular composition, tumor subclone structure and treatment response of RRMM and BME cells from 20 patients. We find that the gain in chromosome 1q (+1q), a high-risk MM aberration that predicts poor prognosis in both newly diagnosed and refractory MM [5, 12], emerges from small subclones over treatment lines and shed light on its molecular

features. Our study reveals large changes in BME cell type composition that include (i) an accumulation of exhausted γδ T-cells and myeloid populations with immunosuppressive features, (ii) depletion of naïve T-cells and the B-cell lineage, and (iii) an expansion of reprogrammed plasmacytoid dendritic cells (pDCs) upon treatment with IMiDs. Based on a co-expression analysis of receptor-ligand pairs we provide mechanistic insight on how RRMM cells might reprogram the BME via inflammatory cytokines and ligands of inhibitory receptors and reveal links to +1q.

Results

We employed single cell transcriptomics on a droplet-based platform to dissect subclone structure, transcriptional heterogeneity, cellular interactions and treatment response in RRMM (**Fig. 1a-c**). The sample set comprised 20 RRMM patients that were refractory to their immediate prior line of treatment with a median of three prior treatments. Of these, 18 patients were refractory to both a proteasome inhibitor and an IMiD, while 2 patients were primarily refractory to initial therapy (**Supplementary Table 1 and S2, Supplementary Data Set 1**). Paired samples before the last treatment and at relapse were analyzed after sorting each sample into a CD138$^+$ myeloma cell fraction and a CD138$^-$ BME fraction that were processed independently for scRNA-seq analysis (**Fig. 1a, b**). In total, 212,404 cells were analyzed that passed stringent quality control with a median of 1143 detected genes and 3070 detected UMIs per cell (**Supplementary Fig. 1a-f**). Two-dimensional embedding using uniform manifold approximation and projection (UMAP) showed that BME immune cells clustered by cell type without the need for further batch effect correction (**Fig. 1c**). A clear separation of plasma/myeloma cells from BME immune cells was apparent from the enrichment of CD138$^+$ cells (**Supplementary Fig. 1g, h**) and the high expression of the plasma cell marker *TNFRSF17* encoding for BCMA. It was further corroborated by automated cell type prediction (**Fig. 1c-e**), using the Human Cell Atlas (HCA) bone marrow data set from 8 healthy donors as reference. Overall, we profiled 83,201 RRMM plasma cells (PCs) (median = 2,189) and 129,203 BME cells (median = 5,383) for an integrated analysis of both tumor and immune cell heterogeneity in RRMM (**Supplementary Table 2**). Our single cell transcriptome analysis was combined with clinical data, including type of treatment and depth of response as well as interphase fluorescence in situ hybridization (iFISH).

scRNA-seq analysis dissects heterogeneity of RRMM tumor cells

To assess inter and intra-patient tumor heterogeneity in RRMM we applied clustering and UMAP embedding of RRMM PCs. Strong transcriptional differences between patients were apparent, except for one cluster harboring cells of multiple patients (**Fig. 2a, Supplementary Fig. 3a**). This cluster could be assigned to non-malignant plasma cells (nPCs) as it had a

normal genome based on a copy number alteration (CNA) analysis of the scRNA-seq data whereas tumor cells of each patient displayed a unique set of numerous chromosomal aberrations (**Supplementary Fig. 2a**). In addition, nPCs derived from RRMM patients cluster together with nPCs from healthy donors (**Supplementary Fig. 2b**).

To characterize RRMM subgroups based on their genomic alterations, we first used the iFISH data that include both CNAs and translocations recurrently detected in MM (**Fig. 2b, , Supplementary Fig. 3b**). Pseudo-bulk expression profiles of individual patients followed the iFISH classification and clustered into transcriptional subtypes consistent with their genomic alterations. The marker gene expression profiles included upregulation of *MAFB* and *NSD2* in patients with t(4;14) translocation, increased *CCND1* expression in patients with t(11;14) translocation [13] and increased expression of ribosomal genes in the subset of five hyperdiploid patients [14]. Major CNAs identified from the scRNA-seq data showed a very good agreement (11/12 and 11/13 detected) to those derived from whole genome sequencing (WGS) data of the same samples (**Fig. 2c, Supplementary Fig. 2c**). In agreement with previous studies [5], we identified a high grade of intra-tumor heterogeneity in RRMM, with a median of three CNA subclones per patient (**Supplementary Fig. 2a**).

The most frequent copy number alteration in our RRMM cohort was +1q with 17/20 cases. In 10/20 patients +1q was subclonal (**Supplementary Fig. 2a**). In addition, the fraction of +1q cells identified from scRNA-seq data was highly correlated with the iFISH data (**Supplementary Fig. 3c**). The only exception was RRMM16 that had a scattered +1q signal, complicating the subclone assignment and indicating a high degree of intra-tumor +1q heterogeneity (**Supplementary Fig. 2a**). Thus, the CNA analysis of the scRNA-seq data resolved complex patterns of (rare) subclones and their specific chromosomal aberrations and made it possible to integrate these profiles with the corresponding transcriptomes at single cell resolution.

The number of clusters per patient increased with the number of cells analyzed. This technical bias made it difficult to assess the correlation between the numbers of clones and clusters (**Supplementary Fig. 3d**). We generally detected more clusters than clones, which probably reflects non-genetic mechanisms that affect gene expression, including epigenetic alterations and microenvironmental influences. Nevertheless, the gene expression-based clustering frequently showed a high degree of overlap with a given subclone type as illustrated for a patient with 5 subclones (**Fig. 2c-g**). In some instances, we also detected rare subclones that did not separate into a distinct transcriptional cluster as depicted for a +1q clone that comprised ~2% of the cells (**Fig. 2c, e, Fig. 2f**). Differential expression analysis between this clone and the genetically most similar without +1q revealed 328 differentially upregulated genes (**Fig. 2h**), which were mostly located on 1q (**Fig. 2i**). These include genes whose increased expression is known to be associated with +1q in MM like *MCL1* [15], *ATF3* [16] or *PSMD4* [17].

Since +1q is known to be associated with poor prognosis in MM [5, 12], we further dissected commonly upregulated genes in +1q subclones to pinpoint the expression of driver genes associated with this genetic aberration.

A gene expression signature predicts +1q in single cells

Based on a differential gene expression analysis across all samples between a +1q clone and the most similar clone without 1q gain we defined a +1q signature based on recurrently upregulated genes (**Fig. 2j, k, Supplementary Data Set 2**). Furthermore, we classified samples according to their +1q state into "not-detected/rare" (+1q <10%), "subclonal" (10% > +1q > 80%) and "dominant" (+1q > 80%) (**Fig. 2j**). The +1q gene expression signature comprised 51 genes and included known drivers of MM pathogenesis that have already been linked to +1q MM such as *ILF2* [16], as well as a number of genes that so far have not been associated with +1q MM like *CTSS* (Cathepsin S), a cysteine protease involved in the recruitment of immunosuppressive myeloid cells [18, 19] (**Fig. 2l, Supplementary Table 4**). Next, we validated our +1q signature against a differential expression analysis of a large bulk RNA-seq data set of +1q detected vs. not-detected samples for newly diagnosed MM patients [20]. Two-thirds of +1q signature genes were also detected in the bulk analysis whereas 18 genes were exclusively detected by scRNA-seq, including *SLAMF7*, *RGS1* and *CTSS* (**Supplementary Fig. 3e, Supplementary Fig. 3f**). In addition, the actin-binding protein *CORO1A* not located on 1q was common in the single cell and bulk RNA-seq analysis, pointing to trans-regulated downstream effects of +1q on gene expression, as described previously for other genes in MM [11]. Notably, top differentially upregulated genes identified in the bulk RNA-seq approach did not primarily locate to chromosome 1 (**Supplementary Fig. 3g**). Finally, we validated the +1q signature against an external MM scRNA-seq data set [11] and validated the correlation of +1q signature expression and +1q (sub)clone abundance (**Supplementary Fig. 3h, i**). Thus, by resolving subclones from copy number alteration analysis and assigning their transcriptional profiles, we derived a +1q gene expression signature. It is based on the differential gene expression analysis of genetically similar subclones with and without +1q in the same sample, which is likely to reduce confounding effects as compared to the bulk RNA-seq analysis.

Subclones with +1q frequently expand during different treatments

We next analyzed the behavior of +1q clones during treatment. As an example, an expansion of +1q cells in RRMM13 upon treatment with the second-generation proteasome inhibitor carfilzomib [21] is shown in **Fig. 3a** and **3b**. Resistance of myeloma cells to the proteasome-inhibitor bortezomib has been linked to +1q and over-expression of *PSMD4* [22], a recurrently upregulated +1q signature gene. The +1q cells did not separate into a dedicated cluster pre-treatment based on their complete transcriptome. However, our +1q signature detected a

lowly-abundant subclone independent of the CNA analysis **(Fig. 3c and 3d)**. Next, we analyzed the relative abundances of subclones defined by their CNA profile in individual patients over treatment to follow clone dynamics. We detected striking differences between patients, ranging from highly stable compositions to complete rearrangements of clonal distributions **(Fig. 3e)**. Interestingly, we observed a clear association of low clone stability and deeper treatment responses, in line with previous observations in newly diagnosed multiple myeloma [23]. Importantly, we did not observe a single case where a +1q subclone became depleted. Rather, +1q clones frequently expanded or remained stable and thus showed a remarkable robustness against different types of treatment in RRMM.

RRMM is associated with large changes in BME composition

We integrated the $CD138^{-}$ compartment of RRMM patients with BME cells of healthy donors from HCA into a joint data set of 406,946 cells (**Fig. 4a**). Fine-grained clustering identified 32 cell types that included all major mononuclear bone marrow cell types and progenitor populations that give rise to myeloid/dendritic, B-cell and erythroid lineages as well as disconnected T/NK cell populations (**Fig. 4a, b, Supplementary Fig. 4a, Supplementary Table 3**). Between RRMM patients and healthy individuals, strong differences in cell type composition were observed, which were mostly in line with previously reported findings **(Fig. 4c, d, Supplementary Fig. 4b**). These include a depletion of $CD4^{+}$/$CD8^{+}$ naive and $CD4^{+}$ memory T-cells [24] and cells of the B-cell lineage [25]. In addition, $CD14^{+}$ and $CD16^{+}$ monocytes [8, 9, 26] and effector T-cell populations [25], including $CD8^{+}$ memory and cytotoxic T-cells as well as γδ T-cells were enriched. Interestingly, we found no enrichment of Treg cells and no depletion of $GZMK^{+}$ memory effector T-cells as described previously based on a scRNA-seq analysis for earlier stages of MM [9]. Whereas NK^{dim} cell frequencies were only slightly higher, immature NK^{bright} cells increased in abundance. In addition, the hematopoietic progenitor populations were strongly decreased in RRMM (**Supplementary Fig. 4b**), in line with previous studies [27]. Interestingly, we found this phenomenon to be associated with enhanced immune activation and inflammation-related signaling in the BME of a subset of patients (**Supplementary Fig. 4c).** Differential expression analysis revealed a strongly enhanced expression of inflammatory cytokines in $CD14^{+}$ monocytes in patients with enhanced inflammation (**Supplementary Fig. 4d)**, indicating that this cell type represents a major driver of inflammation in RRMM. Finally, we observed enhanced expression of the inflammation-induced transcription factor KLF6 [28] and its target genes (**Supplementary Table 4**) across multiple cell types (**Supplementary Fig. 4e**), indicating that an inflammatory BME induces a common transcriptional program.

The BME is reprogrammed by RRMM cells via upregulation of inflammatory cytokines

Reciprocal interactions of tumor and BME cells mediated by cytokines and their corresponding receptors affect several aspects of myeloma pathogenesis including disease progression and treatment resistance [29]. Accordingly, we predicted these cellular interactions based on the expression of ligand-receptor pairs. Across patients, we detected the most pronounced interactions of myeloma cells with the myeloid lineage, in particular with $CD14^+$ and $CD16^+$ monocytes (**Fig. 4e**). Monocytes represent an important component of the niche for the homing of nPCs in the bone marrow [30] and myeloma cells to a large extend maintained the same cellular interactions as nPCs in healthy samples (**Fig. 4f**). However, also a diverse set of links was observed (**Fig. 4f**) that enhanced interactions to $CD14^+$ and $CD16^+$ monocytes, cDC2 cells and pDCs (**Supplementary Fig. 5a**).

We next investigated individual interactions and focused on those that were primarily present in RRMM patients as compared to healthy donors. Several common patterns emerged (**Fig. 4g**). We observed a frequent upregulation of inflammatory cytokines expressed in myeloma cells. These genes included macrophage migration inhibitory factor (*MIF*), amphiregulin (*AREG*), granulin precursor (*GRN*) and chemokine (C-C motif) ligand 3 (*CCL3*), whose corresponding receptors were primarily expressed in myeloid and dendritic cells. Myeloma cells also frequently upregulated *FAM3C*, a ligand of the inhibitory *KIR2DL3* receptor expressed on NK and γδ T-cells [31]. In addition, *CD48* was upregulated in RRMM, which targets the immunomodulatory *CD244* receptor primarily expressed in NK and γδ T-cells [32]. We additionally observed a frequent upregulation of *CD47* in myeloma cells, an integrin-associated receptor protein that inhibits the phagocytosis of target cells [33]. In $CD14^+$ and $CD16^+$ monocytes and in cDC2 cells we observed an upregulation of *CD74* and *CLEC4A* (**Supplementary Fig. 5b**). *CD74* represents the primary surface receptor for *MIF*, and *CLEC4A* is a regulatory receptor that impairs T-cell immunity [34], underlining the importance of myeloid populations in RRMM pathogenesis. Overall, our scRNA-seq based interaction analysis revealed a number of transcriptionally over-expressed cytokines and surface markers in myeloma cells that target specific immune cells and thus might contribute to generating an immunosuppressive BME.

IMiD treatment increases pDCs abundance

We next systematically analyzed the influence of treatments on BME cell type abundances. B-cells were most severely affected in patients treated with IMiDs (4/5 cases), which likely reflects the lineage relationship of B and plasma cells and associated vulnerabilities to these type of drugs [35] (**Supplementary Fig. 6a**). In contrast, pDCs expanded upon IMiD based treatments (5/5 cases), in line with previous studies that have highlighted the importance of pDCs in the pathogenesis of MM, including survival and drug resistance [7]. We therefore

investigated transcriptional characteristics of pDCs in RRMM by performing differential expression analysis between pDCs of healthy donors and RRMM patients (**Supplementary Fig. 6b and S6c**). The pDCs population in RRMM showed an upregulation of *IRF8*, a transcription factor involved in the differentiation of dendritic cells. Importantly, *IRF8* deletion in pDCs has been shown to increase T-cell stimulatory function [36], indicating that increased *IRF8* expression has the opposite effect. In line with this conclusion, we observed an upregulation of inhibitory receptors *CD300A* [37], *CLEC4A* [34] and *LGALS9* [38] in RRMM pDCs. In addition, pDCs displayed upregulated *TNFSF13B*, encoding for the cytokine BAFF, an important niche factor for plasma cells in the bone marrow [30]. Taken together, our data suggest that a reprogrammed pro-tumorigenic pDC phenotype is important for immunosuppression and IMiD resistance in RRMM.

γδ T-cells display features of exhaustion in RRMM

T-cells play a major role in adaptive immunity and are key players in immune surveillance of MM [39]. Therefore, we aimed to identify molecular features of T-cell subsets in RRMM (**Fig. 5a-c, Supplementary Fig. 7a**) that provide information about functional heterogeneity. A recent study has provided evidence for the critical role of GZMK$^+$ CD8$^+$ memory effector T-cells and their depletion in earlier stages of MM progression [9]. In contrast, our data set revealed that CD8$^+$ memory effector T-cells became more abundant in RRMM (**Supplementary Fig. 4b**). Their number further increased upon IMiD-based treatments in 3/7 cases (**Supplementary Fig. 6a**). Interestingly, we observed a co-expansion of Tregs, indicative of a compensatory mechanism in RRMM (**Supplementary Fig. 6a**). Furthermore, CD8$^+$ memory effector T-cells exhibited a strong upregulation of activation markers (e.g., *CD69* and *LAT*) and effector molecules (e.g., *GZMB*, *PRF1*, *GNLY*) in nearly all patients (**Supplementary Fig. 7b, Supplementary Fig. 7c**), pointing to a key role of this T-cell type in RRMM immunosurveillance. At the same time, we observed an upregulation of *LAG3*, *KLRG1*, *IFNG* and *CD47* that have been previously associated with dysfunctional T-cells [40]. This finding might rationalize why activation of GZMK$^+$ CD8$^+$ memory effector T-cells does not result in effective immune responses and clearing of tumor cells in RRMM.

Next, we further examined whether T-cell populations displayed features of exhaustion by upregulation of inhibitory receptors, a phenomenon describing dysfunctional T-cells after chronic antigen stimulation [41]. The highest expression of the exhaustion signature **(Supplementary Table 4)** was detected in γδ T-cells (**Fig. 5d**), for which an impaired immune function during MM disease progression has been reported previously [42]. Furthermore, the abundance of this T-cell subset was highly increased in the BME of RRMM patients compared to healthy donors (**Supplementary Fig. 4b)**. When performing a differential expression analysis between γδ T-cells of RRMM patients and healthy donors, we observed a coordinated

downregulation of ribosomal genes (**Fig. 5e)**, another characteristic previously linked to T-cell exhaustion [43]. We also detected the significant upregulation of several inhibitory receptors, including *VSIR*, *KLRG1*, *LAG3* and *TIGIT* alongside transcription factors such as *NR4A2* and *ID2* (**Fig. 5f**) that have been previously linked to T-cell dysfunction [40]. We observed the parallel upregulation of interferon (IFN) response genes (e.g., *IFITM1*, *STAT1*, *IFI6*) indicating that IFN signaling was associated with the exhausted γδ T-cell phenotype. Expression levels of both exhaustion and IFN-response genes varied across patients (**Fig. 5f**), suggesting different levels of this γδ T-cell exhaustion phenotype. The grade of exhaustion inversely correlated with the expression of effector genes **(Supplementary Table 4)**, which further supports a dysfunctional phenotype of this T-cell subset in RRMM (**Fig. 5g)**. Interestingly, γδ T-cells in patients with translocation (11;14) were associated with weaker expression of exhaustion genes and higher expression of effector genes. In two patients, we further observed the induction of exhaustion signature genes upon treatment in γδ T-cells (**Fig. 5h**). As both patients received different types of treatment, this phenomenon might represent a regimen-independent mechanism of immune evasion in RRMM. Using flow cytometry, we validated the increased abundance of γδ T-cells in RRMM and their elevated levels of PD1 (*PDCD1*) compared to CD8+ T-cells in three patients (**Supplementary Fig. 7e, f**). In contrast, no difference in PD1 expression could be observed between γδ T-cells and $CD8^+$ T-cells in two MGUS (monoclonal gammopathy of undetermined significance) samples, which were used as proxy for healthy individuals. In addition, we confirmed that PD1 expression in γδ T-cells can be increased upon treatment (**Supplementary Fig. 7g**).

A macrophage subtype has a myeloma promoting transcription profile

Next, we characterized tumor associated macrophages (TAMs) in RRMM as several lines of evidence support their critical role for immunosuppression in MM and other tumor entities [44, 45, 46]. The macrophage marker *CD68* was highly expressed in the $CD16^+$ myeloid compartment of RRMM patients (**Supplementary Fig. 8a, b**), pointing to an infiltration of macrophages into the bone marrow of RRMM patients in line with previous observations [10]. The $CD16^+$ subset displayed a high grade of heterogeneity with 10 subtypes based on different sets of marker genes (**Fig. 6a-c**). The most abundant subtype with low expression of *CD68* and high expression of *FCGR3A* (encoding for CD16) was assigned to non-classical monocytes (NCM) expressing the glycolytic enzyme *ALDOA*. Five additional lowly-abundant NCM types were characterized by *CXCL8* (encoding for IL-8), *MEG3, VMO1* and *LYPD2* [47] as well as *FGD2* expression, a marker of migrating monocytes/macrophages [48]. Most subtypes displayed high expression of *FCGR3A* but two subtypes exhibited a $FCGR3A^{dim}$ phenotype and expressed *CD14*. One of them had lower levels of *CD68* and was therefore annotated as intermediate monocytes (IM). Residual populations with high expression of *CD68* were assigned as

TAM1/2/3. A differential expression analysis between NCM and TAM1 subtypes revealed that TAM1 had a high enrichment of IFN-response genes (**Supplementary Fig. 8d, e**) indicating that these cells represent classically activated M1-like macrophages [49]. Complement genes like *C1QA* were highly expressed in TAM2 and TAM3 whereas TAM3 cells additionally expressed *MRC1* (encoding for CD206), a typical marker for immunosuppressive M2-like TAMs [49]. While NCM were largely depleted, IM and all three TAM populations were strongly enriched in RRMM (**Fig. 6d, e**). The distinct expression profiles of IM and all three TAM populations suggest different functional roles in the RRMM BME (**Fig. 6f**). In addition to carrying monocyte markers (e.g., *FCN1*, *VCAN* and *S100A8*), IM cells expressed genes involved in angiogenesis, including *VEGFA*, *SELL* and *HBEGF.* TAM3 cells showed a distinct profile with specific expression of transcription factors (e.g., *HES1*, *PRDM1*), surface proteins (e.g., *CD163*, *CLEC10A*, *FOLR2, ITGAM*) and genes involved in lipid metabolism (e.g., *APOE, APOC1*) (**Fig. 6f**) that have been recently linked to immunosuppressive TAM populations in colorectal and liver cancer [45, 46]. In addition, TAM3 cells displayed immunosuppressive features as they preferentially expressed *CD84*, a gene recently linked to myeloid derived suppressor cells in breast cancer [50], and the negative regulator of T-cell activation *VSIG4* [51]. Interestingly, TAM3 cells showed enhanced expression of *CD38*, a primary drug target in MM [52]. To further characterize the impact of TAMs on the RRMM BME, we constructed an immune cell interaction network that yielded TAMs as nodes of high connectivity (**Fig. 6g**). They displayed overall higher interaction strengths and connectivities compared to the corresponding populations in healthy individuals (**Supplementary Fig. 8g**), which is likely to reflect the activated and inflamed BME in RRMM. We next focused on interactions primarily detected in IM/TAM subtypes and identified a specific interaction between TAM3 and NK^{bright} cells via *IL18* and its receptor (*IL18R1/IL18RAP*) (**Fig. 6h**). IL18 is a key driver of immunosuppression in MM [53] and can suppress NK cell activity in cancer [54]. We validated elevated protein expression levels of CD218a (*IL18R1/IL18RAP*) in NK^{bright} cells as well as IL-18 and CD38 in TAM3 ($CD16^{+}/CD14^{+}/CD11b^{hi}/CD163^{+}$) cells by FACS (**Supplementary Fig. 9a-c**). In contrast, we detected mostly activating interactions of TAM1 and TAM2 with T and NK cells, for example via the expression of *TNFRSF14 – CD160* [55] and *CLEC2B – KLRF1* [56] (**Supplementary Fig. 8h**). Thus, our results indicate that the TAM1-3 subtypes might exert distinct roles in RRMM.

TAM3, NK cells and inflammatory cDC2 abundances are linked to +1q

Next, we asked whether +1q is linked to specific features of a compromised immune microenvironment. Interestingly, we found significantly higher numbers of TAM3 and lowered number of NK^{dim} cells in +1q subclonal/dominant patients when compared to the +1q ND/rare group (**Fig. 7A**). We next subclustered the NK/NKT populations of RRMM patients (**Fig. 7b-d**). In addition to $CD3^{+}$ NKT cells, we identified three NK-cell subtypes that included the immature

NK^{bright} cells characterized by the enhanced expression of *NCAM1*, *KLRC1* (encoding for CD159a/NKG2A) **(Supplementary Fig. 9b**) and *SELL* (encoding for CD62L), activated NK^{dim} cells marked by *CD69* and a population that we termed NK^{dim} effector cells, which expressed high levels of *FCGR3A*, *GZMB* and *PRF1.* Importantly, NK^{dim} effector cells were specifically depleted in +1q patients (**Fig. 7d, e**). We validated abundance differences of TAM3 and NK^{dim} effector subtypes depending on +1q status using flow cytometry analysis in eight samples (**Supplementary Fig. 9d**).

In addition, changes of a cDC2 subtype were correlated with +1q in myeloma cells after subclustering of cDC2 (CD1c^{+}) into three subtypes ("A", "B" and "C") and cycling cDC2 cells (**Supplementary Fig. 10a**). The cDC2_A type cells were characterized by enhanced expression of MHC class II genes. In contrast, cDC2_B cells primarily expressed genes involved in the inflammatory response such as S100A8, S100A9, and CD14 (**Supplementary Fig. 10b-d**) that were also detected in dendritic subtypes in human peripheral blood [57]. Numbers of inflammatory cDC2_B cells were significantly higher in +1q-dominant patients when compared to the ND/rare group (**Supplementary Fig. 10e, f**). Furthermore, the cDC2_B cells expressed enhanced levels of C-type lectin-like receptors including *CLEC7A*, which encodes for DECTIN1 and is involved in immunosuppression in pancreatic cancer [58], as well as *VSIR* encoding for the inhibitory receptor PD-1H [59] (**Supplementary Fig. 10c**). Taken together, our scRNA-seq analysis revealed a compromised BME that is enriched in pro-tumorigenic and depleted in anti-tumorigenic cell types in +1q RRMM patients.

Discussion

Our integrative single cell transcriptome analysis before/after treatment for RRMM dissects intratumor heterogeneity and the interplay between myeloma and BME cells (**Fig. 7f**). By calling CNAs from the scRNA-seq data, subclones with distinct chromosomal aberrations were identified. This approach allowed us to conduct a differential subclone gene expression analysis within the same sample and to define a +1q signature. It comprised genes involved in a variety of biological processes, including apoptosis, proteasome and immune/BME signaling, which might explain resistance mechanisms in diverse treatment scenarios. In addition, our signature faithfully detected lowly-abundant +1q subclones before treatment, which could be exploited for its application to other MM scRNA-seq data sets. The +1q subclones were remarkably robust against different treatments, in line with previous findings on the association of +1q with inferior outcomes in both newly diagnosed MM and RRMM [5, 12]. The RRMM genome and transcriptome profiles obtained in this manner were extended to the analysis of changes of immune cell populations in the BME and the effect of different treatments. The most pronounced BME changes were observed for treatment with IMiDs,

which are known to both directly impact myeloma cells and to modulate immune cells towards enhanced anti-MM immunity [60]. In RRMM, we observed an increase of pDCs upon IMiD treatment that displayed a reprogrammed gene expression profile, suggesting a pro-tumorigenic activity. In line with our observations, previous studies have highlighted the functional role of pDCs in promoting myeloma progression, survival and drug resistance [7]. Thus, reprogrammed pDCs could represent a key cell type for mediating drug-resistance against IMiDs in RRMM.

By dissecting the T-cell population, we found that γδ T-cells were strongly enriched in RRMM compared to healthy individuals and displayed a pronounced exhaustion gene expression signature, which is accompanied by decreased expression of genes involved in effector function. In addition, IFN response genes were upregulated in this cell type. Secretion of type I IFN by myeloma cells has been previously shown to induce Treg expansion and immunosuppression [61], indicating that IFN affects multiple T-cell populations in RRMM. Importantly, the parallel profiling of tumor and immune cells enabled us to predict other direct effects of myeloma cells on γδ T-cells. These include the upregulation of ligands of the inhibitory receptors KIR2DL3 and CD244 by myeloma cells, namely FAM3C [62], which is also a prominent part of the pancreatic cancer secretome [63] and CD48 [32]. CD48 has been previously shown to be overexpressed in MM [64] and is involved in mediating inhibitory signaling in the context of T-cell exhaustion [65]. Given the important role of γδ T-cells in recognizing and killing tumor cells in hematopoietic malignancies, we propose to specifically consider γδ T-cells for the generation of cell-based therapies in RRMM [42].

Inflammatory signaling plays a key role in the pathogenesis of MM [66]. We observed enhanced inflammatory signaling in the BME of approximately half of the BME patients, which is at least partially driven by inflammatory CD14$^+$ monocytes and which induced a common transcriptional program across cell types. This phenomenon is associated with the depletion of hematopoietic progenitor populations, in line with previous observations for IL-1 signaling [67]. In our cellular interaction analysis, myeloma cells displayed an upregulation of inflammatory cytokines (e.g., *CCL3*, *GRN*, *AREG*, *MIF*) that target receptors primarily expressed in the myeloid and dendritic cell compartment. This phenotype could result from the recently reported extensive upregulation of regulatory elements in NF-κB, NOTCH and MTOR signaling in myeloma cells compared to normal B-cells [68]. One of the most recurrently upregulated cytokines was MIF whose expression is associated with inferior outcome in MM [69]. Importantly, monocytes are recruited to inflammatory sites where they differentiate into macrophage or dendritic cells [70]. In line with these findings, we report a crucial role of a tumor-associated macrophage subtype referred to here as TAM3 in RRMM. The gene expression profile of this M2-like population was strikingly similar to immunosuppressive TAMs described recently for several other tumor entities [45, 46, 71]. The TAM3 cells expressed inhibitory immune regulators

and appeared as a central immune cell interaction hub in our network. In addition, they were the primary source of IL18 in RRMM, a key driver of immunosuppression in MM [53]. Our interaction analysis points to a specific IL18 mediated interaction of TAM3 cells with immature NK^{bright} cells that exclusively express the IL18 receptor complex. IL18 acts in a context-dependent manner and can mediate both an activation of NK cells [72] as well as a suppression of NK immunosurveillance [54]. Accordingly, we propose that IL18 secreted by TAM3 cells inhibits the activity of NK^{dim} effector cells in RRMM, probably by influencing differentiation of NK^{bright} cells towards NK^{dim} effector cells. This conclusion is supported by BME characteristics specific for +1q patients who display an increased frequency of TAM3 cells and a depletion of NK^{dim} effector cells. The higher TAM3 cell numbers in +1q patients can be linked to our +1q signature, which, for example, shows significantly higher expression levels of cathepsin S (*CTSS*). Cathepsin S is a cysteine protease involved in the recruitment of immunosuppressive myeloid cells in cancer, including M2-like macrophages [18, 19]. Thus, it represents a promising candidate gene whose over-expression induces a cascade of BME alterations in RRMM as depicted in the model in **Fig. 7f** to rationalize how +1q shapes the tumor microenvironment in RRMM.

Our comprehensive data set of both RRMM cells and their microenvironment in provides a rich resource that complements previous scRNA-seq studies of patient samples at different stages from smoldering MM to fully developed disease [11] and $CD45^+/CD138^-$ cells from the BME [9]. By integrating genetic subclone features and transcriptomes at the single cell level we introduce an approach to cope with the heterogeneity of RRMM. It dissects different clinically relevant disease subtypes and associated molecular phenotypes and their evolution during treatment in a given patient sample. Thus, the analysis can guide personalized clinical decision making by identifying particularly dangerous subclones and their transcriptome like +1q cells at a very early stage when they comprise only 1-2% of the myeloma cells. Furthermore, the high inter-patient tumor heterogeneity, microenvironmental reprogramming and drug response can be evaluated in detail. We anticipate that the insight gained by these approaches introduced in our study for the analysis of RRMM will support the development of novel treatment approaches for the large fraction of MM patients that currently become refractory.

Methods

Patient samples

We studied 20 multiple myeloma patients that were refractory to their immediate prior line of treatment (**Supplementary Table 1**). All patients provided written informed consent before participating in the study. Approval was obtained by the ethics committee of the Medical Faculty at the University of Heidelberg. Bone marrow aspirates were 1:1 diluted in preparation

buffer (PBS with 0.1% BSA and 2 mM EDTA), and mononuclear cell separation was performed by density centrifugation (Bicoll separating solution, Biochrom GmbH) with diluted bone marrow cells (centrifugation 20 min, 1300 g). Cells were carefully aspirated, washed with preparation buffer (centrifugation 5 min at 470 g). Red blood cells were lysed using RCL-buffer (155 mM NH_4Cl, 10 mM $KHCO_3$, 0.1 mM EDTA) for 10 min at room temperature and bone marrow cells were washed (centrifugation 5 min, 470 g) and resuspended in preparation buffer. After cell counting, 1 x 10^7 cells were separated by magnetic-activated cell sorting with anti-CD138 microbeads (Miltenyi Biotec) according to the manufacturer's protocol. Relative plasma cell abundance in whole bone marrow (WBM) as well as plasma cell purity of the $CD138^+$ fractions were measured by flow cytometric analysis. Subsequently, up to 2.5 x 10^6 cells were frozen in 90 % FCS (Sigma-Aldrich) supplemented with 10 % DMSO (Serva Eletrophoresis GmbH) and stored in liquid nitrogen until further use. For samples with low cell numbers, cells were frozen without sorting for CD138 (WBM). For three patients (RRMM05, RRMM08, RRMM12) additional samples were also directly processed for scRNA-seq without freezing and/or sorting in order to evaluate the effect of sorting and freezing on data quality and cell type composition.

Interphase FISH analyses

Interphase FISH analysis (iFISH) was performed in a clinical laboratory on 29/36 $CD138^+$ cell fractions as described previously [73] with probes for chromosomal regions 1q21, 5p15, 5q13, 5q35, 6p21, 8p21, 8q24, 9q34, 11q13, 11q22, 11q22.3, 13q14, 14q32, 15q22, 16q23, 17p13, 19q13 and for translocations t(11;14), t(4;14), t(14;16) or any other IgH- and MYC-rearrangement with unknown region of translocation. Hyperdiploidy was defined as requiring gains of at least two of the three chromosomes 5, 9, and 15. Binary cytogenetic information (detected vs. not-detected) was clustered and visualized with the ComplexHeatmap package using the *dist_letters* function as distance metric (**Supplementary Table 5**).

Bulk whole genome and RNA sequencing

Bulk whole genome sequencing (WGS) data of subject RRMM05 and RRMM17 were analyzed with the software listed in **Supplementary Table 5**. Copy number alterations were computed with the OTP DKFZ WGS pipeline and ACEseq. Bulk RNA-seq data of newly diagnosed MM [20] were processed with the nf-core RNA-seq pipeline and using the STAR alignment software. We separated two groups based on 1q21 cytogenetics (1q21-gain detected vs. not-detected) and performed differential expression analysis with DESeq2 using $p_{adj} < 0.05$, log2 fold change (logFC) > 0.1, similar to scRNA-seq analysis described below.

Single cell RNA sequencing and data preprocessing

Our protocol used viably frozen cells that were thawed at 37 ºC, resuspended in ice-cold PBS and washed twice with cells being collected by centrifugation at 500 g for 4 min. The freezing step had little effect on data quality, major cell type composition and transcriptome as evaluated for three patient samples (**Supplementary Fig. 1a-d**). Cells were counted with a LUNA automated cell counter (Logos Biosystems). Single cell capture, reverse transcription and library preparation were carried out on the Chromium platform (10x Genomics) with the single cell 3' reagent v2 kit (10x Genomics) according to the manufacturer's protocol using 14,000 cells as input per channel. Each of the final libraries were paired-end sequenced (26 bp and 74 bp) on one Illumina HiSeq 4000 lane. Raw sequencing data were processed and aligned to the human genome (GRCh38) using the CellRanger pipeline (10x Genomics, version 3.01). Bone marrow scRNA-seq raw-count data derived from eight healthy individuals (census of immune cells) were downloaded from the HCA data portal (https://data.humancellatlas.org/explore/projects/cc95ff89-2e68-4a08-a234-480eca21ce79). The latter data set was also generated on the Chromium platform and the single cell 3' reagent v2 protocol as used for our samples. Gene symbols of the HCA data set were converted from GENCODE v27 to v28, which was used throughout our study.

Quality control of scRNA-seq data

Cells were excluded from the analysis according to the following criteria: (i) Low-quality single cell libraries with <400 detected genes and >10% mitochondrial counts were removed. (ii) Cell doublets identified with the Scrublet Python package (**Supplementary Table 5**) using the following parameters: *sim_doublet_ratio* = 2; *n_neighbors* = 30; *expected_doublet_rate* = 0.1. All cells with a doublet score > 0.4 were discarded. (iii) We manually excluded doublet clusters or cells that expressed high levels of marker genes of multiple major bone marrow cell types (e.g., CD3D and HBB) that were not detected by Scrublet. (iv) Lowly-abundant platelets ($PPBP^+$) and stromal cells ($CXCL12^+$) were excluded. (v) We defined and removed low-quality clusters as defined by high percentages of mitochondrial gene counts, low housekeeping signature scores and/or low numbers of detected genes as well as no expression of biologically relevant cell type or cell state specific genes. In addition, immunoglobulin genes were removed from the analysis due to the extreme abundance in myeloma cells and associated batch effects caused by ambient immunoglobulin mRNAs. Raw counts for immunoglobulin genes and patient/sample associated information (e.g., sorting fraction, treatment timepoint, FISH data, etc.) were stored as metadata.

General scRNA-seq data analysis

Quality control, normalization, data integration and unbiased clustering of single cell transcriptomes was conducted with the Seurat v3 package and the other software listed in

Supplementary Table 5. Single cell RNA-seq data were normalized and highly variable genes were identified using the SCTransform method. Technical or biological confounding effects like mitochondrial counts or cell cycle stages were regressed out using the *vars.to.regress* argument in SCTransform. Principal component analysis (PCA) was used for linear dimensionality reduction using the top 3,000 variable genes. The number of principal components used for downstream clustering was determined using the *ElbowPlot* function in Seurat. For integration of multiple data sets, the reciprocal PCA method implemented in Seurat or Harmony was used with default parameters if not stated otherwise. Reciprocal PCA yielded better results for very large data sets and was used for the HCA BME data set of eight donors using one male and one female donor as reference. Harmony performed better and was more flexible for (multiple) smaller data sets. Shared nearest neighbor graphs were computed using the *FindNeighbors* function in Seurat and cells were clustered using the Louvain algorithm and UMAP embedding in two-dimensional space.

Pseudo-bulk profiles of myeloma scRNA-seq data of individual patients were generated using the *AverageExpression* function in Seurat using scaled normalized counts of the top 3000 variable genes. Pearson correlation coefficients between average expression values were computed using the *cor* function in Seurat and clustered with the ComplexHeatmap package. For visualization, diagonal values (=1) were set to 0. Differentially expressed genes between groups of cells were identified using Wilcoxon rank-sum test ($p_{adj} < 0.05$, logFC > 0.1) using either the *FindMarkers* function in Seurat or the Presto tool. We merged transcriptional clusters if their average gene expression profiles were highly correlated and if they were characterized by similar cell type-specific marker genes. Cell cycle (G1, S, G2M phase) and signature scores (e.g., housekeeping) were assigned to each cell using the *CellCycleScoring* and *AddModuleScore* function in Seurat. Signature gene lists were derived from the Molecular Signature Database (https://www.gsea-msigdb.org/gsea/index.jsp) or defined manually based on literature (**Supplementary Table 4**). Gene set enrichment analysis was performed using the hypeR R package. Data were visualized in R using Seurat and other software tools (**Supplementary Table 5**). For visualization of gene expression differences, we removed a set of 12 non-informative blacklist genes that are highly upregulated across all cell types in multiple myeloma (*SLC25A6*, *CD99*, *IL3RA*, *CSF2RA*, *MTRNR2L8*, *MTRNR2L12*, *ARL6IP4*, *POLR2F*, *GTPBP6*, *EEF1A1*, *H3F3A*, *TMSB4X*). Changes in relative abundances of cell types were calculated using custom code in R. For the analysis of changes in cell type composition upon treatment, we removed lowly-abundant cell types that mainly include progenitor populations and set a threshold of ≥ 5 cells.

Cell type annotation and analysis

Data of all samples were merged and clustered using Seurat. Non-malignant plasma cells and myeloma cells were identified based on expressing high levels of *TNFRSF17*, *SDC1*, *SLAMF7* and *CD38*. BME cell types were first annotated individually for the HCA and the RRMM data sets and then combined for detailed annotation. The integrated BME data set was subdivided into three major groups, namely T/NK cells, myeloid/DC, B/erythrocyte lineages plus progenitors. These groups were annotated individually using classical immune cell type marker expression and literature sources according to the description given in **Supplementary Table 3**. The RRMM BME cell type annotation was corroborated by automated cell type annotation with SingleR (**Supplementary Table 5**), using the HCA data set and associated cell type labels as reference after downsampling to a maximum of 1000 cells per cell type.

Analysis of copy number alterations and +1q expression signature

Copy number changes in individual plasma/myeloma cells were identified using the InferCNV tool (**Supplementary Table 5**) that averages the expression of adjacent genes over large genomic regions. As reference, we used nPCs derived from the HCA data set and profiled CNAs in myeloma cells of every patient individually as well as all nPCs from the RRMM data set combined. The required gene order file that harbors genomic locations of individual genes was derived from the CellRanger Software (10x Genomics v3.01). For running InferCNV, SCTransform corrected counts were used as input along with the following settings: *cutoff* = 0.1, *cluster_by_groups* = F, *analysis_mode* = “subclusters”. The performance of InferCNV to call copy number changes was evaluated by comparing single cell CNA profiles with bulk WGS and cytogenetic data (iFISH) of the same $CD138^+$ sample. Subclones per patient were identified by cutting the dendogram of clustered single cell CNA profiles using the *cutree* function of the dendextend package with a minimum of 40 cells per subclone. Tumor subclone annotations were then mapped to transcriptional profiles using Seurat. In order to identify common genes across patients upregulated in +1q, we performed differential expression analysis (Wilcoxon rank-sum test, *p*-value < 0.05, logFC > 0.1) in individual patients between one +1q clone and its closest relative without 1q gain as determined by InferCNV. Genes (excluding mitochondria encoded genes) that were upregulated in +1q clones in at least 5 out of 10 patients and that were located in the chromosomal 1q region were used to define a +1q signature that comprised 51 genes (**Supplementary Table 4**). We excluded genes that are not located on 1q (which could indicate for trans-regulation) due to a limited correlation between their average gene expression and the number of +1q cells across patients. The +1q signature score in single cells was computed with the *AddModuleScore* function in Seurat. Raw count matrices derived from Ledergor et al. [11] were downloaded from the GEO data base at accession number GSE117156 and patients were grouped into +1q groups according to the

CNA analysis in the manuscript. InferCNV analysis of the AL04 donor with subclonal +1q was performed as described above with nPCs derived from the same study. To quantify subclone composition changes upon treatment, we defined a *CNA clone stability score.* It uses relative subclone abundances pre- and post-treatment to calculate CNA clone stability scores per patient and treatment according to $\sum(\text{fraction}_{minor}/\text{fraction}_{major})$/N clones.

Cellular interaction analysis

Cellular interactions between cell types were computed based on ligand-receptor co-expression using the CellPhoneDB (v2.0) tool with default settings and log-transformed SCTransform normalized counts as input (**Supplementary Table 5**). Analysis of interactions between myeloma and immune cells as well as its comparison with the interactions of nPCs with immune cells in healthy individuals CellPhoneDB was conducted for each patient individually. Only cell types with >20 cells per patient were included. To calculate the average number of interactions, we summed all significant (p-value < 0.05) interactions between two cell types per patients/donor and averaged this number over patients/donors. To calculate the interactions strength between two cell types across patients, we summed up the mean expression of all ligand and receptor pairs as calculated by CellPhoneDB across patients. For the CellPhoneDB analysis of interactions of myeloid $CD16^+$ subsets, all cells of a given cell type from all patients/donors were pooled and a network was generated for healthy donors and RRMM patients individually. In this network, cell types are the nodes with their top four interaction partners shown as connecting edges and weighted according to the average strengths and the number of edges.

Flow cytometry analyses

For analysis of T-cells, cells were suspended in staining buffer (PBS with 0.5% BSA) and incubated according to the manufacturer's instructions with fluorochrome-labeled antibodies for 30 min at 4°C, then washed for two times. Flow cytometry analyses were performed on a BD FACSLYRIC flow cytometer; data were analyzed using FlowJo V10.7.1 software (BD). To characterize TAM and NK cells, intracellular staining was performed using a transcription factor buffer set (BD Biosciences, Heidelberg, Germany) according to the manufacturer´s instructions. Briefly, cells were resuspended in PBS and incubated for 15 minutes at 4°C with fluorochrome-labeled monoclonal surface antibodies. After washing with 2 ml of PBS, cells were permeabilized with Fix/Perm buffer for 45 minutes at 4°C, washed two times with perm/wash buffer, resuspended in an adequate amount of perm/wash buffer, and incubated with intracellular antibodies for 45 minutes at 4°C. Subsequently, cells were washed two times with Perm/Wash buffer and resuspended in 0.5% paraformaldehyde in PBS. Flow cytometry analyses were performed on a BD FACSymphony™ flow cytometer. Data were analyzed using

FlowJo V10.7.1 software (BD). All antibodies used in this study are listed in **Supplementary Table 6**. In addition, the dyes 7-AAD (Becton Dickinson) and Fixable Viability Dye eFluor 506 (ThermoFisher Scientific) were used for cell staining during FACS analysis.

Data availability

The original scRNA-seq sequencing data sets generated in this study (ID: EGAD00001006903) as well as the four samples additionally analyzed by WGS for validation of the CNA analysis (ID: EGAD00001006903) have been deposited in the European Genome-phenome Archive under the study accession number EGAS00001004805 [https://ega-archive.org/studies/EGAS00001004805]. These data are available under restricted access to comply with German and European data protection regulations, and can be obtained upon application to the linked data access committee. The processed scRNA-seq data used in this study are publicly available at Gene Expression Omnibus under accession number GSE161801 [https://www.ncbi.nlm.nih.gov/geo/query/acc.cgi?acc=GSE161801]. The scRNA-seq data of healthy bone marrow donors were from the Human Cell Atlas database [census of immune cells, https://data.humancellatlas.org/explore/projects/cc95ff89-2e68-4a08-a234-480eca21ce79]. Additional supplementary data sets are listed in **Supplementary Table 7**. Source data are provided with this paper.

Code availability

The software used for the data analysis for the different sequencing readouts is listed in **Supplementary Table 5**. Custom code is provided via a Github repository at https://github.com/RippeLab/RRMM [74].

References

1. Kumar SK*, et al.* Multiple myeloma. *Nat Rev Dis Primers* **3**, 17046 (2017).

2. Kumar SK*, et al.* Improved survival in multiple myeloma and the impact of novel therapies. *Blood* **111**, 2516-2520 (2008).

3. Rajkumar SV. Multiple myeloma: 2016 update on diagnosis, risk-stratification, and management. *Am J Hematol* **91**, 719-734 (2016).

4. Bolli N*, et al.* Heterogeneity of genomic evolution and mutational profiles in multiple myeloma. *Nat Commun* **5**, 2997 (2014).

5. Ziccheddu B*, et al.* Integrative analysis of the genomic and transcriptomic landscape of double-refractory multiple myeloma. *Blood Adv* **4**, 830-844 (2020).

6. Bailur JK*, et al.* Early alterations in stem-like/resident T cells, innate and myeloid cells in the bone marrow in preneoplastic gammopathy. *JCI Insight* **5**, (2019).

7. Chauhan D*, et al.* Functional interaction of plasmacytoid dendritic cells with multiple myeloma cells: a therapeutic target. *Cancer Cell* **16**, 309-323 (2009).

8. Sponaas AM*, et al.* The proportion of CD16(+)CD14(dim) monocytes increases with tumor cell load in bone marrow of patients with multiple myeloma. *Immun Inflamm Dis* **3**, 94-102 (2015).

9. Zavidij O*, et al.* Single-cell RNA sequencing reveals compromised immune microenvironment in precursor stages of multiple myeloma. *Nature Cancer* **1**, 493-506 (2020).

10. Zheng Y*, et al.* Macrophages are an abundant component of myeloma microenvironment and protect myeloma cells from chemotherapy drug-induced apoptosis. *Blood* **114**, 3625-3628 (2009).

11. Ledergor G*, et al.* Single cell dissection of plasma cell heterogeneity in symptomatic and asymptomatic myeloma. *Nat Med* **24**, 1867-1876 (2018).

12. Abdallah N*, et al.* Clinical characteristics and treatment outcomes of newly diagnosed multiple myeloma with chromosome 1q abnormalities. *Blood Adv* **4**, 3509-3519 (2020).

13. Broyl A*, et al.* Gene expression profiling for molecular classification of multiple myeloma in newly diagnosed patients. *Blood* **116**, 2543-2553 (2010).

14. Weinhold N*, et al.* NPM1 is overexpressed in hyperdiploid multiple myeloma due to a gain of chromosome 5 but is not delocalized to the cytoplasm. *Genes Chromosomes Cancer* **49**, 333-341 (2010).

15. Slomp A*, et al.* Multiple myeloma with 1q21 amplification is highly sensitive to MCL-1 targeting. *Blood Adv* **3**, 4202-4214 (2019).

16. Samo AA*, et al.* MCL1 gene co-expression module stratifies multiple myeloma and predicts response to proteasome inhibitor-based therapy. *Genes Chromosomes Cancer* **57**, 420-429 (2018).

17. Fabris S*, et al.* Transcriptional features of multiple myeloma patients with chromosome 1q gain. *Leukemia* **21**, 1113-1116 (2007).

18. Yang M*, et al.* Cathepsin S-mediated autophagic flux in tumor-associated macrophages accelerate tumor development by promoting M2 polarization. *Mol Cancer* **13**, 43 (2014).

19. Wilkinson RD*, et al.* CCL2 is transcriptionally controlled by the lysosomal protease cathepsin S in a CD74-dependent manner. *Oncotarget* **6**, 29725-29739 (2015).

20. Seckinger A, *et al.* Target Expression, Generation, Preclinical Activity, and Pharmacokinetics of the BCMA-T Cell Bispecific Antibody EM801 for Multiple Myeloma Treatment. *Cancer Cell* **31**, 396-410 (2017).

21. Jakubowiak AJ, *et al.* Treatment outcomes in patients with relapsed and refractory multiple myeloma and high-risk cytogenetics receiving single-agent carfilzomib in the PX-171-003-A1 study. *Leukemia* **27**, 2351-2356 (2013).

22. Shaughnessy JD, Jr., *et al.* Pharmacogenomics of bortezomib test-dosing identifies hyperexpression of proteasome genes, especially PSMD4, as novel high-risk feature in myeloma treated with Total Therapy 3. *Blood* **118**, 3512-3524 (2011).

23. Jones JR, *et al.* Clonal evolution in myeloma: the impact of maintenance lenalidomide and depth of response on the genetics and sub-clonal structure of relapsed disease in uniformly treated newly diagnosed patients. *Haematologica* **104**, 1440-1450 (2019).

24. Gu Y, *et al.* Low absolute CD4(+) T cell counts in peripheral blood predict poor prognosis in patients with newly diagnosed multiple myeloma. *Leuk Lymphoma* **61**, 1869-1876 (2020).

25. Pessoa de Magalhaes RJ, *et al.* Analysis of the immune system of multiple myeloma patients achieving long-term disease control by multidimensional flow cytometry. *Haematologica* **98**, 79-86 (2013).

26. Bolzoni M, *et al.* IL21R expressing CD14(+)CD16(+) monocytes expand in multiple myeloma patients leading to increased osteoclasts. *Haematologica* **102**, 773-784 (2017).

27. Bruns I, *et al.* Multiple myeloma-related deregulation of bone marrow-derived CD34(+) hematopoietic stem and progenitor cells. *Blood* **120**, 2620-2630 (2012).

28. Zhang Y, *et al.* Kruppel-like factor 6 is a co-activator of NF-kappaB that mediates p65-dependent transcription of selected downstream genes. *J Biol Chem* **289**, 12876-12885 (2014).

29. Manier S, Kawano Y, Bianchi G, Roccaro AM, Ghobrial IM. Cell autonomous and microenvironmental regulation of tumor progression in precursor states of multiple myeloma. *Curr Opin Hematol* **23**, 426-433 (2016).

30. Belnoue E, Tougne C, Rochat AF, Lambert PH, Pinschewer DD, Siegrist CA. Homing and adhesion patterns determine the cellular composition of the bone marrow plasma cell niche. *J Immunol* **188**, 1283-1291 (2012).

31. Pende D, *et al.* Killer Ig-Like Receptors (KIRs): Their Role in NK Cell Modulation and Developments Leading to Their Clinical Exploitation. *Front Immunol* **10**, 1179 (2019).

32. Agresta L, Hoebe KHN, Janssen EM. The Emerging Role of CD244 Signaling in Immune Cells of the Tumor Microenvironment. *Front Immunol* **9**, 2809 (2018).

33. Jaiswal S*, et al.* CD47 is upregulated on circulating hematopoietic stem cells and leukemia cells to avoid phagocytosis. *Cell* **138**, 271-285 (2009).

34. Uto T*, et al.* Clec4A4 is a regulatory receptor for dendritic cells that impairs inflammation and T-cell immunity. *Nat Commun* **7**, 11273 (2016).

35. Kronke J*, et al.* Lenalidomide causes selective degradation of IKZF1 and IKZF3 in multiple myeloma cells. *Science* **343**, 301-305 (2014).

36. Sichien D*, et al.* IRF8 Transcription Factor Controls Survival and Function of Terminally Differentiated Conventional and Plasmacytoid Dendritic Cells, Respectively. *Immunity* **45**, 626-640 (2016).

37. Borrego F. The CD300 molecules: an emerging family of regulators of the immune system. *Blood* **121**, 1951-1960 (2013).

38. Zhu C*, et al.* The Tim-3 ligand galectin-9 negatively regulates T helper type 1 immunity. *Nat Immunol* **6**, 1245-1252 (2005).

39. Leblay N, Maity R, Hasan F, Neri P. Deregulation of Adaptive T Cell Immunity in Multiple Myeloma: Insights Into Mechanisms and Therapeutic Opportunities. *Front Oncol* **10**, 636 (2020).

40. Li H*, et al.* Dysfunctional CD8 T Cells Form a Proliferative, Dynamically Regulated Compartment within Human Melanoma. *Cell* **176**, 775-789 e718 (2019).

41. Wherry EJ, Kurachi M. Molecular and cellular insights into T cell exhaustion. *Nat Rev Immunol* **15**, 486-499 (2015).

42. Castella B, Foglietta M, Riganti C, Massaia M. Gamma delta T cells in the bone marrow of myeloma patients: a paradigm of microenvironment-induced immune suppression. *Front Immunol* **9**, 1492 (2018).

43. Wherry EJ*, et al.* Molecular signature of CD8+ T cell exhaustion during chronic viral infection. *Immunity* **27**, 670-684 (2007).

44. Scavelli C*, et al.* Vasculogenic mimicry by bone marrow macrophages in patients with multiple myeloma. *Oncogene* **27**, 663-674 (2008).

45. Sharma A*, et al.* Onco-fetal Reprogramming of Endothelial Cells Drives Immunosuppressive Macrophages in Hepatocellular Carcinoma. *Cell* **183**, 377-394 e321 (2020).

46. Zhang L*, et al.* Single-Cell Analyses Inform Mechanisms of Myeloid-Targeted Therapies in Colon Cancer. *Cell* **181**, 442-459 e429 (2020).

47. Hofer TP*, et al.* slan-defined subsets of CD16-positive monocytes: impact of granulomatous inflammation and M-CSF receptor mutation. *Blood* **126**, 2601-2610 (2015).

48. Huber C, Martensson A, Bokoch GM, Nemazee D, Gavin AL. FGD2, a CDC42-specific exchange factor expressed by antigen-presenting cells, localizes to early endosomes and active membrane ruffles. *J Biol Chem* **283**, 34002-34012 (2008).

49. Mantovani A, Marchesi F, Malesci A, Laghi L, Allavena P. Tumour-associated macrophages as treatment targets in oncology. *Nat Rev Clin Oncol* **14**, 399-416 (2017).

50. Alshetaiwi H*, et al.* Defining the emergence of myeloid-derived suppressor cells in breast cancer using single-cell transcriptomics. *Sci Immunol* **5**, eaay6017 (2020).

51. Vogt L*, et al.* VSIG4, a B7 family-related protein, is a negative regulator of T cell activation. *J Clin Invest* **116**, 2817-2826 (2006).

52. van de Donk N, Usmani SZ. CD38 Antibodies in Multiple Myeloma: Mechanisms of Action and Modes of Resistance. *Front Immunol* **9**, 2134 (2018).

53. Nakamura K*, et al.* Dysregulated IL-18 Is a Key Driver of Immunosuppression and a Possible Therapeutic Target in the Multiple Myeloma Microenvironment. *Cancer Cell* **33**, 634-648 e635 (2018).

54. Terme M*, et al.* IL-18 induces PD-1-dependent immunosuppression in cancer. *Cancer Res* **71**, 5393-5399 (2011).

55. Muscate F, Stetter N, Schramm C, Schulze Zur Wiesch J, Bosurgi L, Jacobs T. HVEM and CD160: Regulators of Immunopathology During Malaria Blood-Stage. *Front Immunol* **9**, 2611 (2018).

56. Welte S, Kuttruff S, Waldhauer I, Steinle A. Mutual activation of natural killer cells and monocytes mediated by NKp80-AICL interaction. *Nat Immunol* **7**, 1334-1342 (2006).

57. Villani AC*, et al.* Single-cell RNA-seq reveals new types of human blood dendritic cells, monocytes, and progenitors. *Science* **356**, eaah4573 (2017).

58. Daley D*, et al.* Dectin 1 activation on macrophages by galectin 9 promotes pancreatic carcinoma and peritumoral immune tolerance. *Nat Med* **23**, 556-567 (2017).

59. Johnston RJ*, et al.* VISTA is an acidic pH-selective ligand for PSGL-1. *Nature* **574**, 565-570 (2019).

60. Quach H, *et al.* Mechanism of action of immunomodulatory drugs (IMiDS) in multiple myeloma. *Leukemia* **24**, 22-32 (2010).

61. Kawano Y, *et al.* Blocking IFNAR1 inhibits multiple myeloma-driven Treg expansion and immunosuppression. *J Clin Invest* **128**, 2487-2499 (2018).

62. Efremova M, Vento-Tormo M, Teichmann SA, Vento-Tormo R. CellPhoneDB: inferring cell-cell communication from combined expression of multi-subunit ligand-receptor complexes. *Nat Protoc* **15**, 1484-1506 (2020).

63. Gronborg M, *et al.* Biomarker discovery from pancreatic cancer secretome using a differential proteomic approach. *Mol Cell Proteomics* **5**, 157-171 (2006).

64. Hosen N, *et al.* CD48 as a novel molecular target for antibody therapy in multiple myeloma. *Br J Haematol* **156**, 213-224 (2012).

65. Blackburn SD, *et al.* Coregulation of CD8+ T cell exhaustion by multiple inhibitory receptors during chronic viral infection. *Nat Immunol* **10**, 29-37 (2009).

66. Musolino C, Allegra A, Innao V, Allegra AG, Pioggia G, Gangemi S. Inflammatory and Anti-Inflammatory Equilibrium, Proliferative and Antiproliferative Balance: The Role of Cytokines in Multiple Myeloma. *Mediators Inflamm* **2017**, 1852517 (2017).

67. Pietras EM, *et al.* Chronic interleukin-1 exposure drives haematopoietic stem cells towards precocious myeloid differentiation at the expense of self-renewal. *Nat Cell Biol* **18**, 607-618 (2016).

68. Ordonez R, *et al.* Chromatin activation as a unifying principle underlying pathogenic mechanisms in multiple myeloma. *Genome Res* **30**, 1217-1227 (2020).

69. Wang Q, *et al.* MIF as a biomarker and therapeutic target for overcoming resistance to proteasome inhibitors in human myeloma. *Blood* **136**, 2557-2573 (2020).

70. Richards DM, Hettinger J, Feuerer M. Monocytes and macrophages in cancer: development and functions. *Cancer Microenviron* **6**, 179-191 (2013).

71. Molgora M, *et al.* TREM2 Modulation Remodels the Tumor Myeloid Landscape Enhancing Anti-PD-1 Immunotherapy. *Cell* **182**, 886-900 e817 (2020).

72. Ni J, *et al.* Single-Cell RNA Sequencing of Tumor-Infiltrating NK Cells Reveals that Inhibition of Transcription Factor HIF-1alpha Unleashes NK Cell Activity. *Immunity* **52**, 1075-1087 e1078 (2020).

73. Neben K, *et al.* Combining information regarding chromosomal aberrations t(4;14) and del(17p13) with the International Staging System classification allows stratification of myeloma

patients undergoing autologous stem cell transplantation. *Haematologica* **95**, 1150-1157 (2010).

74. Steiger S, Tirier SM, Rippe K. Nextflow data processing pipelines and R code for single cell RNA sequencing analysis of relapsed and refractory multiple myeloma. *Zenodo*, https://doi.org/10.5281/zenodo.5532552 (2021).

Author contributions

Study design and coordination: MR, KR. Acquisition of patient samples: MR, NG, AB, LJ. Acquisition of data: SMT, JPM, KB, MA, AS, DH. Analysis of data: SMT, SS, AP, NC, HS, OS, KR. Drafting of manuscript: SMT, KR. Review and editing of manuscript: all authors. Supervision: KR, MR, HG, NW, CMT, MH, OS.

Competing Interests

HG – Grants/provisions: Amgen, BMS, Celgene, Chugai, Janssen, Johns Hopkins University, Sanofi; Research support: Amgen, BMS, Celgene, Chugai, Janssen, Incyte, Molecular Partners, Merck Sharp and Dohme (MSD), Sanofi, Mundipharma GmbH, Takeda, Novartis; Advisory Boards: Adaptive Biotechnology, Amgen, BMS, Celgene, Janssen, Sanofi, Takeda; Honoraria: Amgen, BMS, Celgene, Chugai, GlaxoSmithKline (GSK), Janssen, Novartis, Sanofi. *CMT* – Grants/provisions: Pfizer, Daiichi Sankyo, BiolineRx; Research support: Abbvie, Amgen, AstraZeneca, Bayer, Boehringer Ingelheim, Bristol Myers Squibb, Celgene, Eisai, Fresenius, Gilead, Hexal, Janssen, Jazz Pharmaceuticals, MSD, Novartis, Pharmamar, Pfizer, Roche, Shire, Takeda, Affimed; Advisory boards: Pfizer, Janssen. The other authors declare no competing interests.

Acknowledgements

We thank the NCT Heidelberg Molecular Precision Oncology Program for technical support and funding through project number HIPO K43R. Further funding was by the Dietmar-Hopp Foundation. We are grateful to Adelheid Cerwenka for discussions, and thank the DKFZ Genomics and Proteomics as well as the Omics IT and Data Management core facilities for their help and services.

Figure legends

Figure 1. RRMM samples and scRNA-seq data set

(**a**) Typical disease course of RRMM patients and sampling time-points of the study. (**b**) Workflow for scRNA-seq including CD138 sorting of myeloma cells. (**c**) Analysis and integration overview of scRNA-seq data. Left: Clustering and cell type identification was based on scRNA-seq of 212,404 cells from primary mononuclear bone marrow samples of RRMM patients (n = 20) as shown by an UMAP embedding colored by sample without batch effect correction. Middle: Myeloma cells subclones according to genetic aberrations were identified while changes BME cells were evaluated against the HCA data set of healthy donors. Right: By integrating these data, subclone specific interactions of myeloma cells with BME cells were revealed. (**d**) UMAP embedding as shown in panel *c* but colored according to expression of the plasma cell marker *TNFRSF17*. (**e**) Same as panel *d* but colored for plasma cells.

Figure 2. scRNA-seq analysis of RRMM tumor cells

(**a**) UMAP embedding of RRMM PCs colored by subject. Dashed circle marks non-malignant plasma cells (nPCs). The pie chart inset shows the nPCs fraction colored according to patient. (**b**) Pearson correlation matrix of averaged gene expression levels per patient. Top, cytogenetic information; bottom, averaged gene expression levels of five MM-subtype specific genes. (**c**) CNAs of exemplary patient sample. Top, heatmap of RRMM05 CNA signal normalized against nPCs derived from the HCA bone marrow reference data set. Horizontal lines divide subclones; bottom, coverage plot showing total copy number derived from whole genome sequencing data of the same sample; * indicates agreement between both modalities in detecting major CNAs. (**d**) UMAP embedding of RRMM05 tumor cells colored by transcriptional cluster. (**e**) Same as panel *d* but colored according to CNA clone. (**f**) Same as panel *d* but with subclone 2 cells in black. (**g**) Bar plot of relative subclone abundances in RRMM05. (**h**) Heatmap of 348 differentially expressed genes between subclone 2 and 3. Example genes are listed and shown in bold letters if located on 1q. Subclone 3 has been downsampled to 1000 cells for visualization. Thresholds for differential expression using two-sided Wilcoxon rank-sum test were p-value < 0.05 (Bonferroni-adjusted) and logFC > 0.1. (**i**) Bar plot showing fraction of differentially upregulated genes in subclone 2 compared to subclone 3 by chromosomal location. (**j**) Scatter plot of +1q transcriptome signature score against fraction of +1q cells as determined by InferCNV. The Pearson's correlation coefficient was $R = 0.86$ ($p = 2.1 \cdot 10^{-11}$). Grouping of samples/patients into +1q "not-detected/rare", and "dominant" is indicated by vertical lines. (**k**) Violin plot of +1q signature scores for cells of the three different +1q groups "ND/rare" (n = 22,206 cells), "subclonal" (n = 13,345) and "dominant" (n = 45,946). The p-values from a Kruskal-Vallis-test were $< 2 \cdot 10^{-16}$. Box plot: center line,

median; box limits, first and third quartile; whiskers, minimum/maximum values. (**l**) Genes of the +1q signature. The bar plot shows the fraction of subjects in which individual genes were upregulated in +1q clones when compared to the most similar clone without +1q.

Figure 3. Treatment response of +1q subclones

(**a**) Heatmap of RRMM13 CNA signal (pre- and posttreatment combined). Horizontal lines divide subclones. (**b**) Bar plot of fraction of +1q cells pre- and posttreatment for RRMM13. (**c**) UMAP embedding of RRMM13 tumor cells before treatment. Coloring depicts transcriptional cluster (left), +1q cells (middle) and +1q signature score (right). (**d**) Violin plot and associated histogram of +1q signature score of RRMM13 tumor cells pre-treatment. The positive predictive value is PPV = 0.076 for the score of 0.2 indicated by the dashed line. (**e**) Line plot of subclone fraction derived from the CNA analysis per patient pre/post treatment. Red lines mark +1q subclones. Top, CNA stability score per patient; bottom, best treatment response.

Figure 4. Analysis of cellular interaction of myeloma and BME cells

(**a**) UMAP embedding of $CD138^{-}$ BME cells colored by cell type (**Supplementary Table 3**). The inset provides a schematic overview for the location of major cell types. (**b**) Gene expression dot plot of major marker genes for individual cell types. (**c**) UMAP embedding as shown in panel *a* as point-density plot split in RRMM vs. healthy donors. (**d**) Bar plot of cell type fractions for RRMM (right) and healthy (left) donors individually. (**e**) Cellular interactions of myeloma tumor cells and BME cell types. Ligand-receptor expression was ordered according to the number of detected interactions. (**f**) Bar plot of the cumulative number of interactions detected in a given BME cell type in RRMM samples in comparison to healthy donors. Grey, all interactions; red, interactions gained in RRMM; blue, interactions lost in RRMM. Only selected interactions detected in ≥3 patients were included. (**g**) Gene expression dot plot of ligand-receptor expression. Left, expression in nPCs/myeloma tumor cells; right, expression in BME cell types. Only interactions that increased between myeloma tumor cells and immune cell types as compared to nPCs are shown. Interactions involving inflammatory cytokines are highlighted.

Figure 5. T-cell heterogeneity in RRMM patient vs. healthy donor samples

(**a**) UMAP embedding of subclustered T-cell populations colored by cell type of the combined RRMM/healthy data set. (**b**) UMAP embedding split and colored by RRMM/healthy status. (**c**) Heatmap showing averaged gene expression levels of T-cell population marker genes in the combined data set. (**d**) Gene expression dot plot showing exhaustion signature score levels in T-cell subpopulations in RRMM. (**e**) Volcano plot of differentially expressed genes using

two-sided Wilcoxon rank-sum test in γδ T-cells (RRMM vs. healthy). Thresholds for differential expression were p-value < 0.05 (Bonferroni-adjusted) and logFC > 0.1. (**f**) Heatmap of clustered average gene expression and exhaustion signature score of γδ T-cells (RRMM vs. healthy). Selected genes are indicated to the right and signature expression levels of IFN response and ribosomal genes are shown at the bottom. Only samples with >60 profiled γδ T-cells were included. (**g**) Scatterplot of average effector score and average exhaustion score in γδ T-cells across patients. Regression line and 95% confidence interval are shown with data points colored according to iFISH status of t(11;14). The Pearson's correlation coefficient was R = -0.63 (p = 0.0087). (**h**) Changes of gene expression in γδ T-cells and $CD8^+$ cytotoxic T-cells upon treatment with IMiD. Top, violin plot of exhaustion signature score of donor BM1 (n = 369 cells) and patients RRMM01 (n = 65/425 cells) or RRMM07 (n =130/95 cells) pre- and posttreatment. Pairwise Bonferroni-adjusted p-values from a two-sided Wilcoxon rank-sum test are shown. Box plot: center line, median; box limits, first and third quartile; whiskers, minimum/maximum values. Bottom, heatmap of average gene expression levels of genes of the exhaustion signature.

Figure 6. $CD16^+$ monocyte heterogeneity in RRMM

(**a**) UMAP embedding of subclustered $CD16^+$ monocytes colored by subtype. (**b**) UMAP embedding showing expression levels of major marker genes of subtypes indicated in panel *a*. (**c**) Violin plot of selected marker genes of subtypes in panel *a* split into RRMM vs. healthy. (**d**) UMAP embedding as shown in panel *a* split and colored by donor status (RRMM/healthy). (**e**) Beeswarm plot for the comparison of $CD16^+$ monocyte subtype fractions between RRMM (n = 19) and healthy (n = 8) individuals with Bonferroni-adjusted p-values from a two-sided Wilcoxon rank-sum test. Red center line indicates mean. (**f**) Heatmap showing selected differentially expressed genes between RRMM-enriched $CD16^+$ monocyte subtypes. (**g**) Network plot of predicted cellular interactions between immune cell subsets in RRMM. Every cell type is connected to its 4 top interacting cell types based on the sum of interaction strengths. The node size corresponds to the number of connected cell types and the coloring corresponds to the interaction strength. (**h**) Gene expression dot plot of ligand/receptors of RRMM-enriched $CD16^+$ monocyte subtypes (left) and associated interaction partners in the RRMM BME (right). Shown are selected interactions that are stronger in the IM/TAM3 subtypes as compared to TAM2 and TAM3.

Figure 7. BME changes in +1q RRMM

(**a**) Changes in cell type composition in dependence of +1q. The bar plot in the center shows $\log_{10}$ Bonferroni-adjusted p-values from a two-sided Wilcoxon rank-sum test of differences in

cell type fractions between +1q ND/rare vs. subclonal/dominant groups. The box plots display the comparison of NK^{dim} cells (left) and TAM3 (right) subtype fractions between patients with +1q ND/rare (*n* = 7) vs. subclonal/dominant (*n* = 12). Box plot: center line, median; box limits, first and third quartile; whiskers, minimum/maximum values. (**b**) UMAP embedding of subclustered NK cells colored by subtype. (**c**) Gene expression dot plot for main marker genes of NK subtypes. (**d**) UMAP embedding showing GZMB and PRF1 expression levels (left) and point-density plot split in +1q ND/rare and +1q-subclonal/dominant groups (right). (**e**) Beeswarm plot for the comparison of the NK^{dim} effector cells fraction between patients with +1q ND/rare (*n* = 7), subclonal (*n* = 5) and dominant (*n* = 7). Pairwise Bonferroni-adjusted *p*-values from a two-sided Wilcoxon rank-sum test are indicated. Red center line: mean. (**f**) Scheme of transcriptional changes and altered cellular interactions in RRMM with +1q specific changes colored in red.

Supplementary Information

Subclone-specific microenvironmental impact and drug response in refractory multiple myeloma revealed by single cell transcriptomics

Stephan M. Tirier, Jan-Philipp Mallm, Simon Steiger, Alexandra M. Poos, Mohamed H. S. Awwad, Nicola Giesen, Nicola Casiraghi, Hana Susak, Katharina Bauer, Anja Baumann, Lukas John, Anja Seckinger, Dirk Hose, Carsten Müller-Tidow, Hartmut Goldschmidt, Oliver Stegle, Michael Hundemer, Niels Weinhold, Marc S. Raab, Karsten Rippe

Inventory of Supplementary Information

Supplementary Figures

Supplementary Fig. 1. Quality control of single cell transcriptome data
Supplementary Fig. 2. Copy number alteration (CNA) analysis from scRNA-seq data
Supplementary Fig. 3. Non-malignant plasma cells, cytogenetics and +1q signature
Supplementary Fig. 4. Cell types in the bone marrow environment
Supplementary Fig. 5. Predicted cellular interactions
Supplementary Fig. 6. Cell type abundance changes upon treatment and pDC reprogramming
Supplementary Fig. 7. T-cell subtype characterization
Supplementary Fig. 8. Analysis of CD16$^+$ monocytes
Supplementary Fig. 9. FACS validation of scRNA-seq data for NK and TAM3 cells
Supplementary Fig. 10. Characterization of inflammatory cDC2 population

Supplementary Tables

Supplementary Table 1. Overview of RRMM patients
Supplementary Table 2. Cell numbers of samples studied
Supplementary Table 3. Cell types and marker genes
Supplementary Table 4. Gene expression signatures
Supplementary Table 5. Data analysis software
Supplementary Table 6. Antibodies for fluorescence activated cell sorting
Supplementary Table 7. Inventory of supplementary data sets

Supplementary Data Sets

Additional data sets on samples and the analysis results derived from the different sequencing readouts are provided as separate files in Microsoft Excel format. An inventory for these data sets is given in Supplementary Table 7.

Supplementary Figures

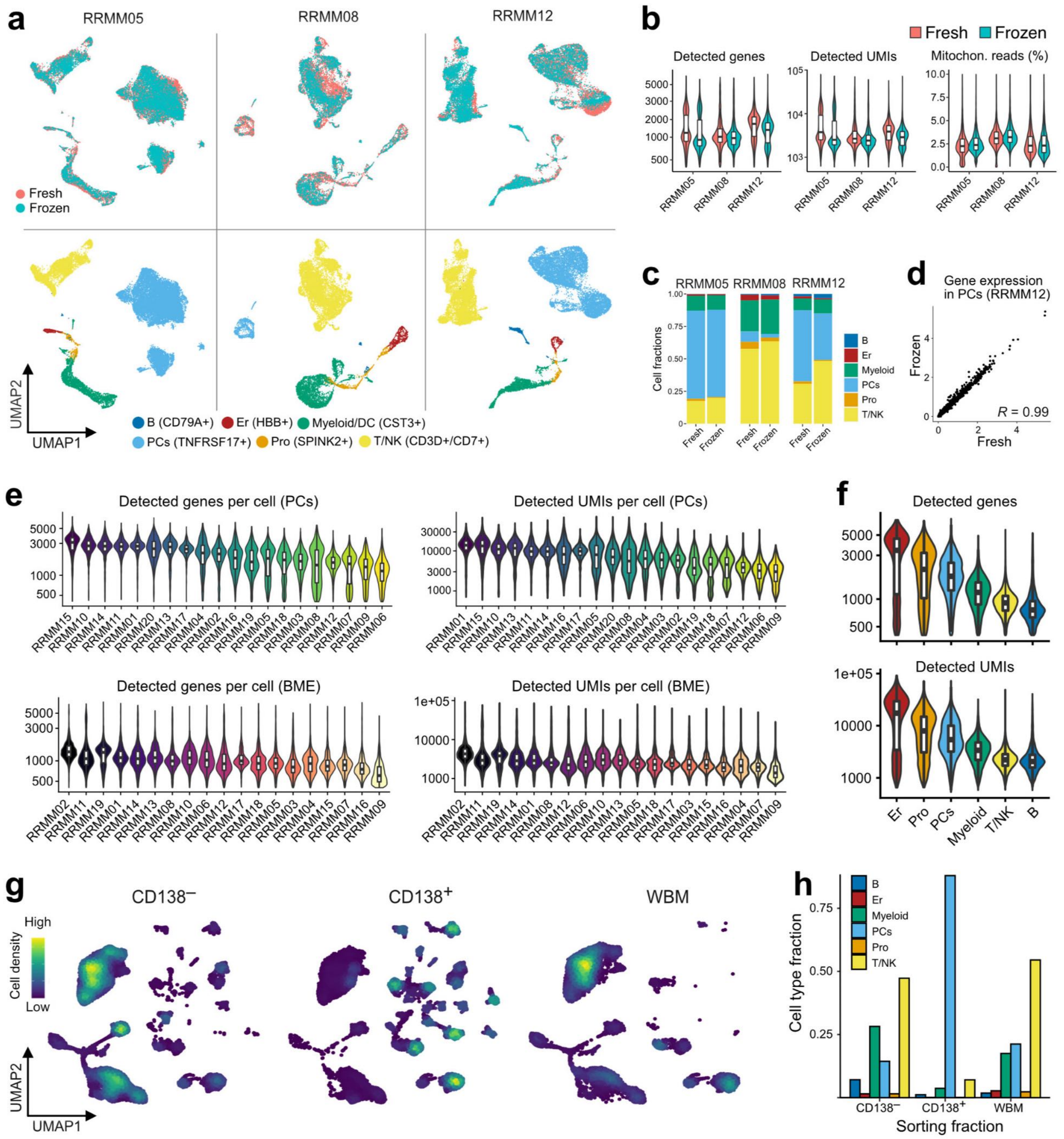

Supplementary Fig. 1. Quality control of single cell transcriptome data

For cell numbers see Supplementary Table 2 and Supplementary Data Set 1. (**a**) UMAP embedding of three patients without batch effect correction. Top, colored by fresh vs. frozen processing; bottom, colored by major cell type. (**b**) Violin plots of scRNA-seq quality metrics for processing of fresh vs. frozen cells. (**c**) Stacked bar plot of relative abundances of major cell types (fresh vs. frozen). (**d**) Scatter plot of averaged genes expression of plasma cells (PCs) in RRMM12 (fresh vs. frozen). A Pearson's correlation coefficient of $R = 0.99$ was obtained. (**e**) Violin plot of quality metrics per patient for PCs (top) and for BME cells (bottom). (**f**) Violin plot of detected genes and UMIs per major cell type. (**g**) UMAP embedding as shown in Fig. 1C as point-density plot split into the three different sorting fractions. (**h**) Bar plot of major cell type fractions per sorting fraction. Box plot: center line, mean; box limits, first and third quartile; whiskers: minimum/maximum values.

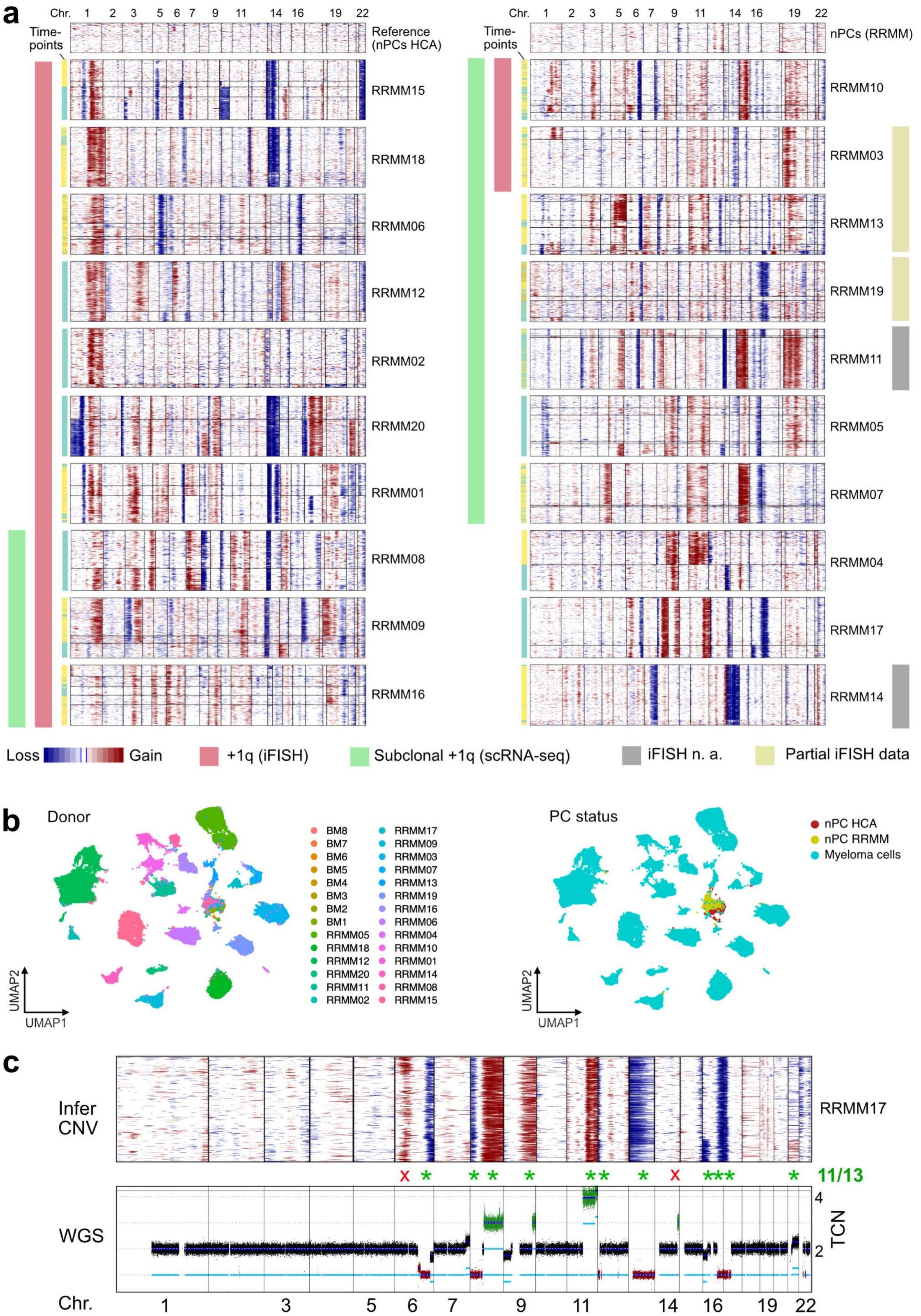
a
Chr.
1
2
3
5
6
7
9
11
14
16
19
22
Time-points
Reference (nPCs HCA)
nPCs (RRMM)
RRMM15
RRMM18
RRMM06
RRMM12
RRMM02
RRMM20
RRMM01
RRMM08
RRMM09
RRMM16
RRMM10
RRMM03
RRMM13
RRMM19
RRMM11
RRMM05
RRMM07
RRMM04
RRMM17
RRMM14
Loss
Gain
+1q (iFISH)
Subclonal +1q (scRNA-seq)
iFISH n. a.
Partial iFISH data
b
Donor
BM8
BM7
BM6
BM5
BM4
BM3
BM2
BM1
RRMM05
RRMM18
RRMM12
RRMM20
RRMM11
RRMM02
RRMM17
RRMM09
RRMM03
RRMM07
RRMM13
RRMM19
RRMM16
RRMM06
RRMM04
RRMM10
RRMM01
RRMM14
RRMM08
RRMM15
UMAP2
UMAP1
PC status
nPC HCA
nPC RRMM
Myeloma cells
c
Infer CNV
RRMM17
11/13
WGS
TCN
4
2
Chr.
1
3
5
6
7
9
11
14
16
19
22

Supplementary Fig. 2. Copy number alteration (CNA) analysis from scRNA-seq data

(**a**) Heatmap of CNA signal per patient normalized against non-malignant plasma cells (nPCs) derived from the HCA bone marrow reference data set using the InferCNV package. Horizontal lines divide subclones. Top left, nPC reference from HCA data set; top right, nPCs from RRMM samples. (**b**) UMAP of PCs derived from RRMM patients and healthy donors (HCA) combined. Left, colored by donor; right, colored by plasma cell status. (**c**) CNAs of exemplary patient sample RRMM17. Top, heatmap of RRMM17 CNA signal normalized against nPCs derived from the HCA bone marrow reference data set with the horizontal lines divide subclones; bottom, coverage plot showing total copy number derived from whole genome sequencing data of the same sample; * indicates agreement between both modalities in detecting major CNAs; TCN, total copy number.

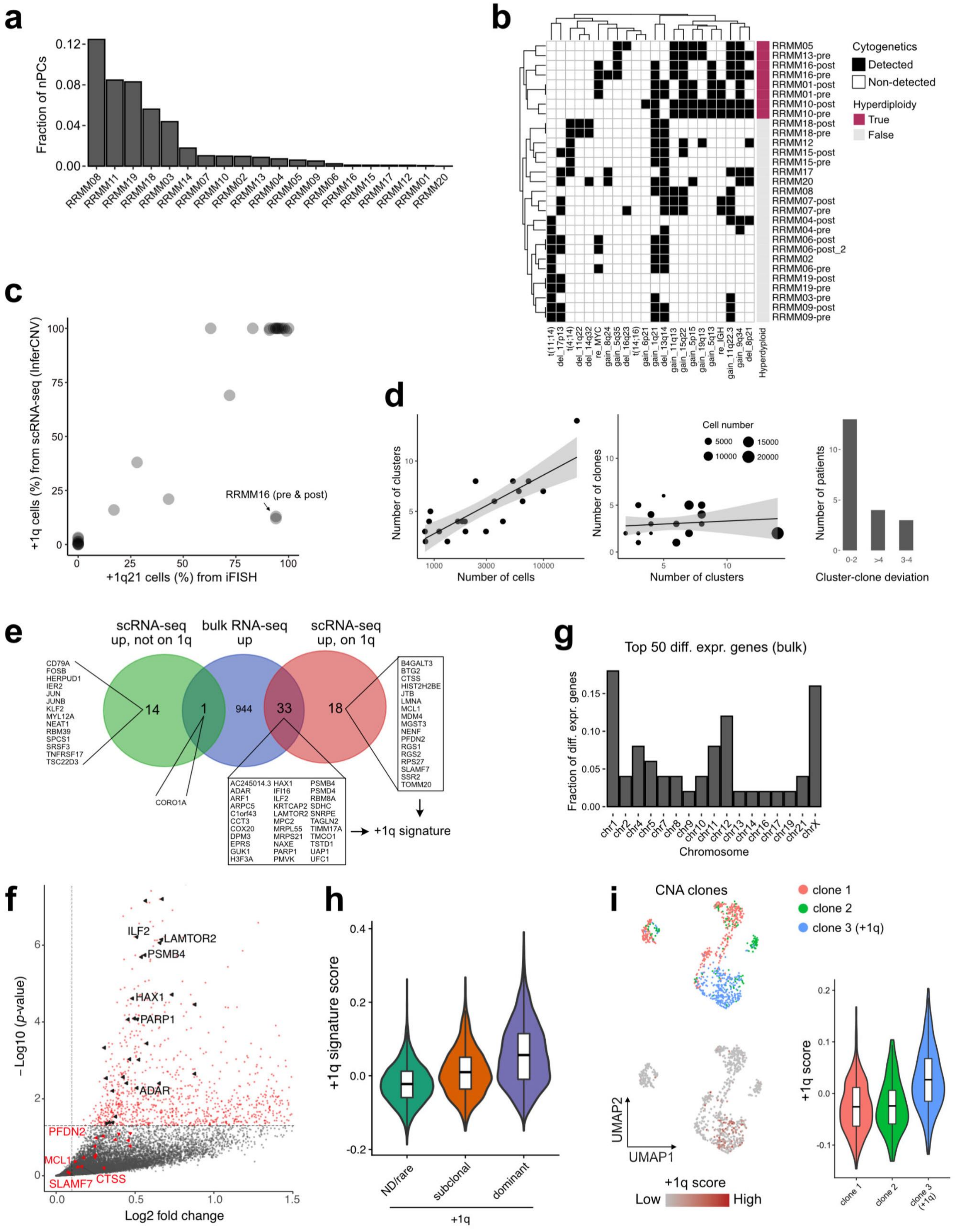

Supplementary Fig. 3. Non-malignant plasma cells, cytogenetics and +1q signature

(**a**) Bar plot of non-malignant plasma cell (nPC) abundances per patient. (**b**) Hierarchical clustering of binary cytogenetic information per sample. (**c**) Scatterplot showing fraction of +1q cells as detected by scRNA-seq vs iFISH. The Pearson's correlation coefficient was R = 0.86 (p = $1.6 \cdot 10^{-9}$). (**d**) Left and middle: Scatterplots showing relationships between number of cells, clusters and CNA clones

per patient. Regression line and 95% confidence interval are indicated. The Pearson's correlation coefficient was $R = 0.81$ ($p = 1.6\cdot10^{-5}$, clusters vs. cells) and $R = 0.13$ ($p = 0.58$, clones vs. clusters). Right: Bar plot showing deviation of cluster and clone numbers per patient subdivided in three groups (n = 0-2, 3-4, >4). (**e**) Venn-diagram depicting the overlap between genes upregulated in +1q clones using scRNA-seq and genes identified in +1q patients with an independent bulk RNA-seq data set. The latter was from newly diagnosed multiple myeloma patients classified into 1q21 detected vs. not-detected based on cytogenetics. (**f**) Volcano plot of differentially upregulated genes computed with DESeq2 [1] in +1q patients from bulk RNA-seq data [2]. The red dots indicate genes above the thresholds indicated by the dashed lines of $p < 0.05$ (Bonferroni-adjusted) and a log2 fold change (logFC) > 0.1. (**g**) Bar plot showing fraction of the top 50 differentially upregulated genes by chromosomal location in +1q patients compared to patients without gain of 1q. (**h**) Violin plot of +1q signature scores for the three different +1q groups and cell numbers of 12,635 (ND/rare), 1,869 (subclonal) and 2,505 (dominant). Data were taken from Ledergor et al.[3] with Bonferroni-adjusted p-values $< 2\cdot10^{-16}$ from a Kruskal-Vallis-test. (**i**) Analysis of donor AL04 [3]. Clone 1 (red, n = 318), clone 2 (green, n = 126) and clone 3 (blue, n = 292, +1q) were defined based on the CNA analysis and plotted in the UMAP on the top. Clone 3 with +1q can also identified based on its +1q transcription signature score. This is shown in the UMAP plot (bottom) and the Violin plot (right) that shows the +1q signature scores for the different CNA clones with Bonferroni-adjusted p-values $< 2\cdot10^{-16}$ from a Kruskal-Vallis-test. Box plot: center line, median; box limits, first and third quartile; whiskers, minimum/maximum values.

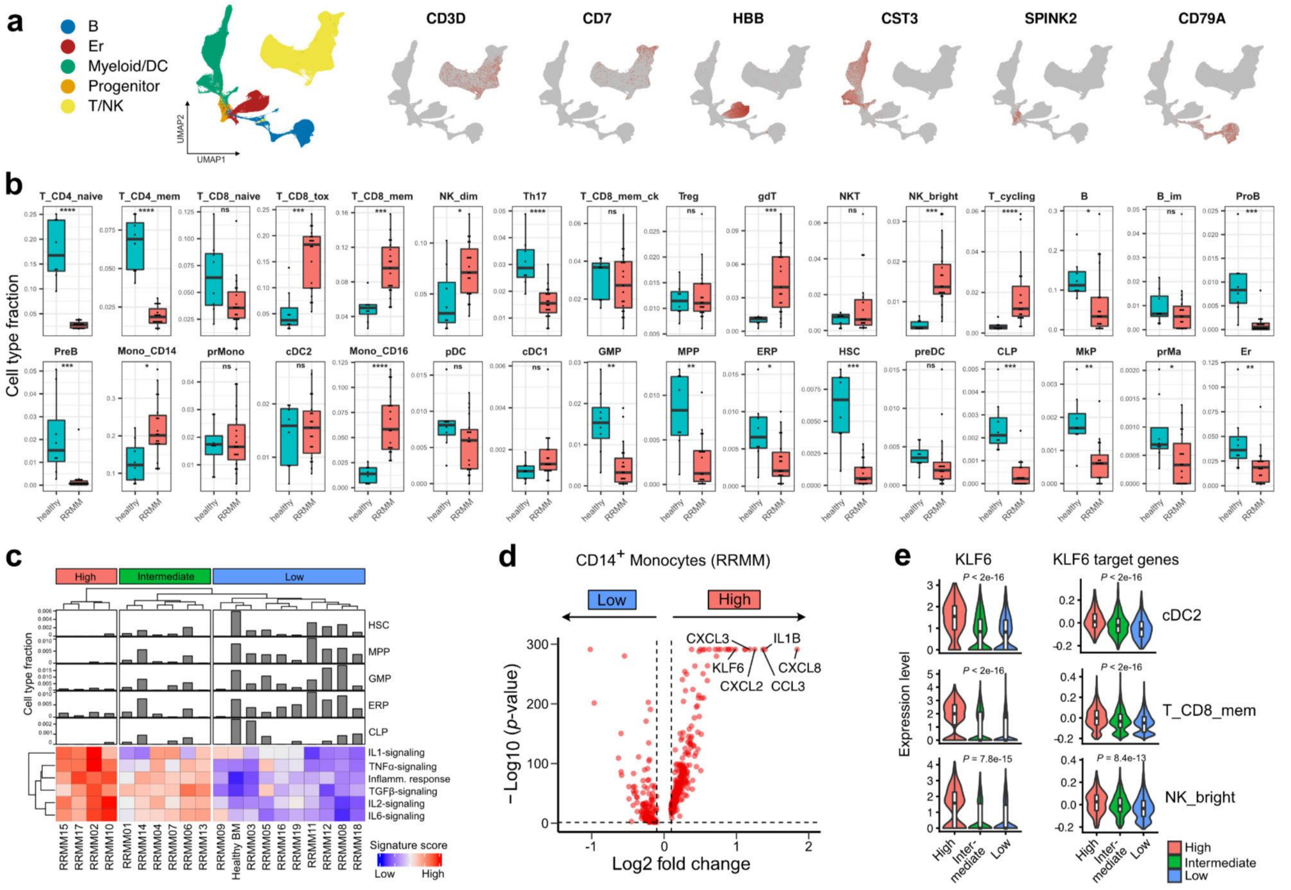

Supplementary Fig. 4. Cell types in the bone marrow environment

(**a**) UMAP embedding of CD138⁻ cells of the bone marrow environment (BME) colored by major cell type and by expression of indicated marker genes. (**b**) Box plot for the comparison of cell type fractions between RRMM patients (n = 19) and healthy individuals (n = 8). The Bonferroni adjusted p-values from a two-sided Wilcoxon rank-sum test are indicated with ns, $p > 0.05$; *, $p < 0.05$; **, $p < 0.01$; ***, $p < 0.001$; ****, $p < 0.0001$. Box plot: center line, median; box limits, first and third quartile; whiskers, minimum/maximum values. (**c**) Heatmap of average immune signaling pathway signature scores of BME cell types from individual patients and healthy donor reference. The heatmap is split according to the dendrogram (k = 3) into three groups with high, intermediate and low scores for the indicated signaling pathways. The bar plots display the relative abundance of the different progenitor cell types in the BME. (**d**) Volcano plot of differential expression analysis between $CD14^+$ monocytes derived from "low" and "high" patient groups shown in panel *c*. Thresholds for differential expression were p-value < 0.05 from a Bonferroni-adjusted Wilcoxon rank-sum test and logFC > 0.1 or < -0.1, respectively. (**e**) KLF6 expression levels (left) and KLF6 target gene module scores (right) are shown as Violin plots of the immune signaling groups defined in panel *c* in cDC2 (high, n = 241; intermediate, n = 1,010; low, n = 590), $CD8^+$ memory effector T-cell (high, n = 1,267; intermediate, n = 7,133; low, n = 4,684) and NK^{bright} cell (high, n = 237; intermediate, n = 1,274; low, n = 504) populations. Bonferroni-adjusted p-values are from a Kruskal-Vallis-test. Box plot: center line, median; box limits, first and third quartile; whiskers, minimum/maximum values.

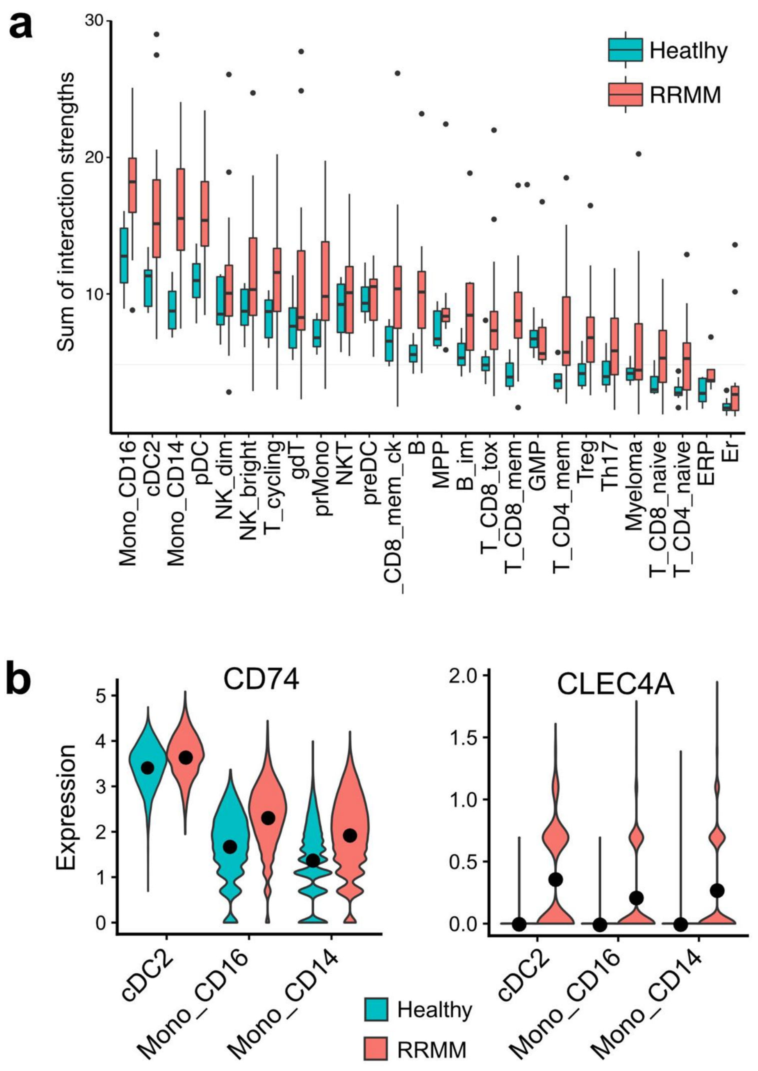

Supplementary Fig. 5. Predicted cellular interactions

(**a**) Box plot of interaction strength in RRMM (*n* = 19) and healthy (*n* = 19) individuals. The sum of interaction strengths as mean expression of ligand and receptor of nPCs (HCA) or myeloma tumor cells (RRMM) and the indicated immune cell types per sample was plotted. Box plot: center line, median; box limits, first and third quartile; whiskers, minimum/maximum values. (**b**) Violin plots of CD74 and CLEC4A gene expression levels in myeloid cell types in RRMM vs healthy individuals. Center-points indicate mean values.

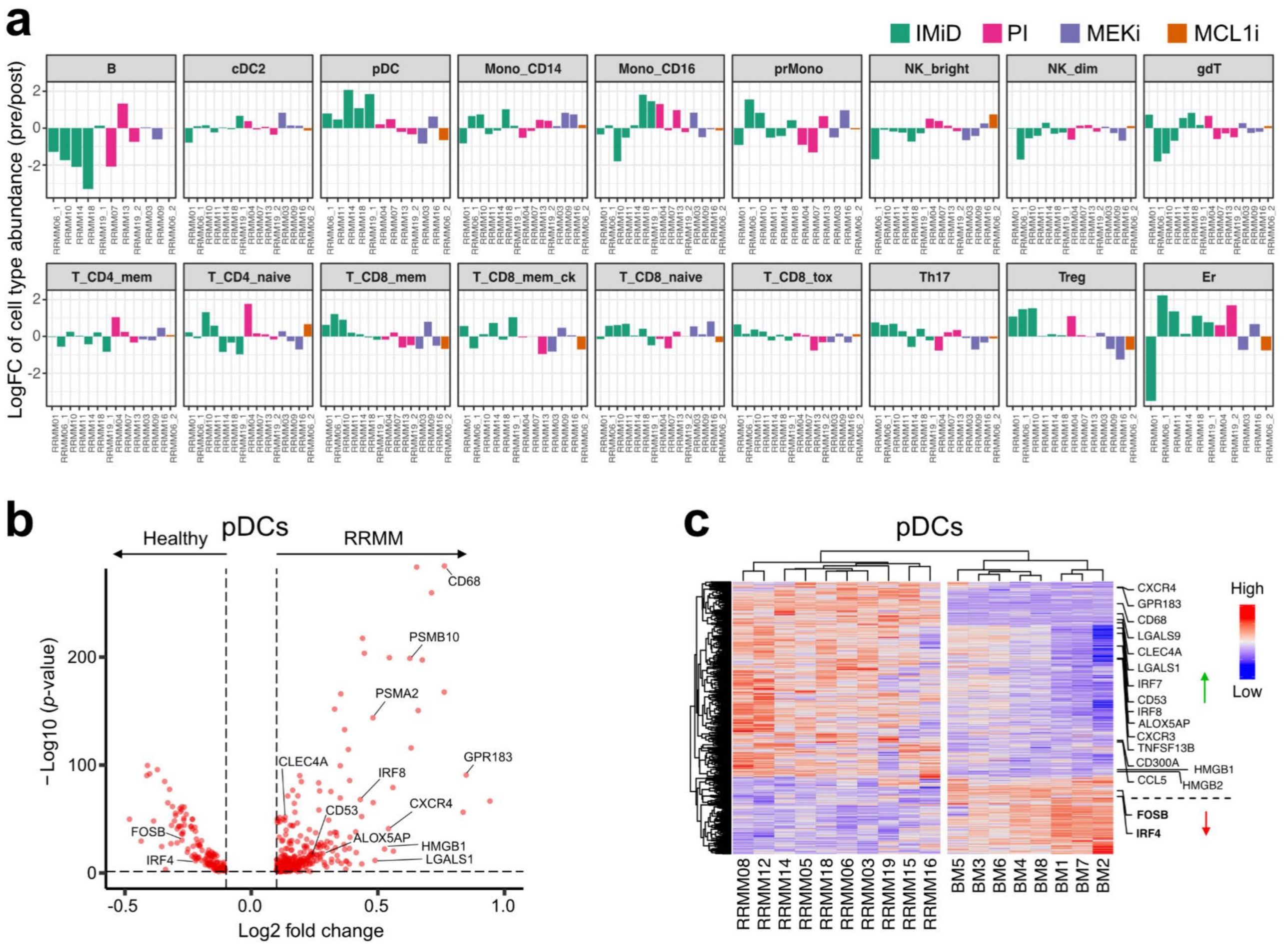

Supplementary Fig. 6. Cell type abundance changes upon treatment and pDC reprogramming

(**a**) Bar plots of relative log fold changes in cell type abundances during treatment for the indicated patient samples. Only the most abundant cell types are shown. (**b**) Volcano plot of differentially expressed genes (RRMM vs. healthy) in pDCs. Thresholds for differential expression were *p*-value < 0.05 from a Bonferroni-adjusted Wilcoxon rank-sum test and logFC > 0.1 or < -0.1, respectively. (**c**) Heatmap showing clustered average genes expression levels of differentially expressed genes between pDCs in RRMM vs. healthy per patient/donor. Selected genes are listed on the left side of the heatmap. Only patient samples with >25 profiled cells are shown.

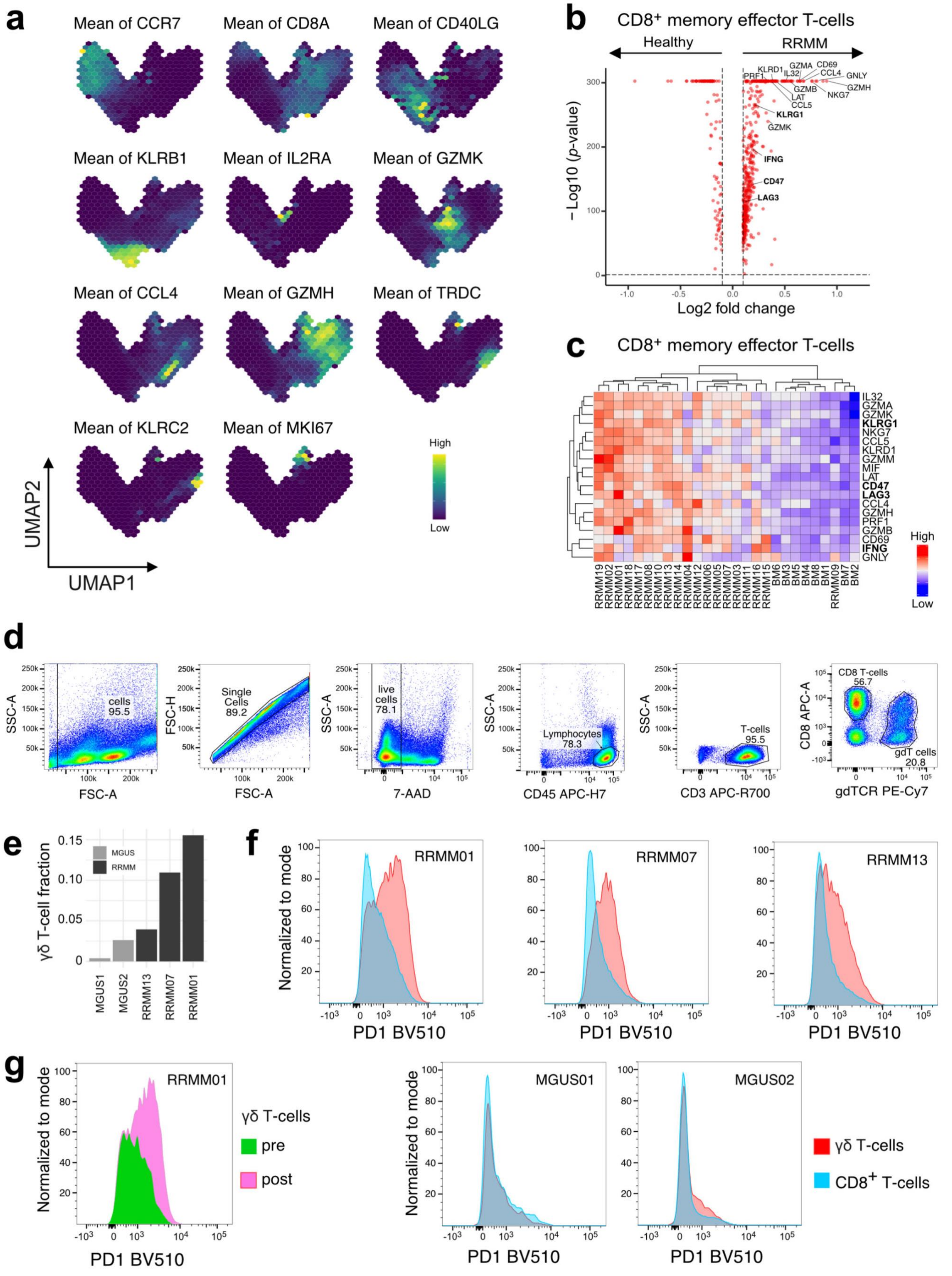

Supplementary Fig. 7. T-cell subtype characterization

(**a**) UMAP plot of expression levels of T-cell subtype marker genes. Gene expression of cells in similar locations are binned in hexagons and averaged. (**b**) Volcano plot of differentially expressed

genes (RRMM vs. healthy) in CD8$^+$ memory effector T-cells. Thresholds for differential expression were *p*-value < 0.05 from a Bonferroni-adjusted Wilcoxon rank-sum test and logFC > 0.1 or < -0.1, respectively. (**c**) Clustered heatmap of selected differentially expressed genes in CD8$^+$ memory effector T-cells in RRMM patient vs. healthy donor samples. (**d**) FACS gating strategy for the analysis of γδ T-cells and CD8$^+$ T-cells. (**e**) Bar plot showing fractions of γδ T-cells in RRMM samples. For comparison samples from MGUS (monoclonal gammopathy of undetermined significance) patients were also included in the analysis. (**f**) FACS histograms for PD1 protein expression levels in γδ T-cells vs. CD8$^+$ T-cells in MGUS and RRMM samples. (**g**) FACS histograms for protein expression levels of PD1 in γδ T-cells pre/post treatment of patient RRMM01.

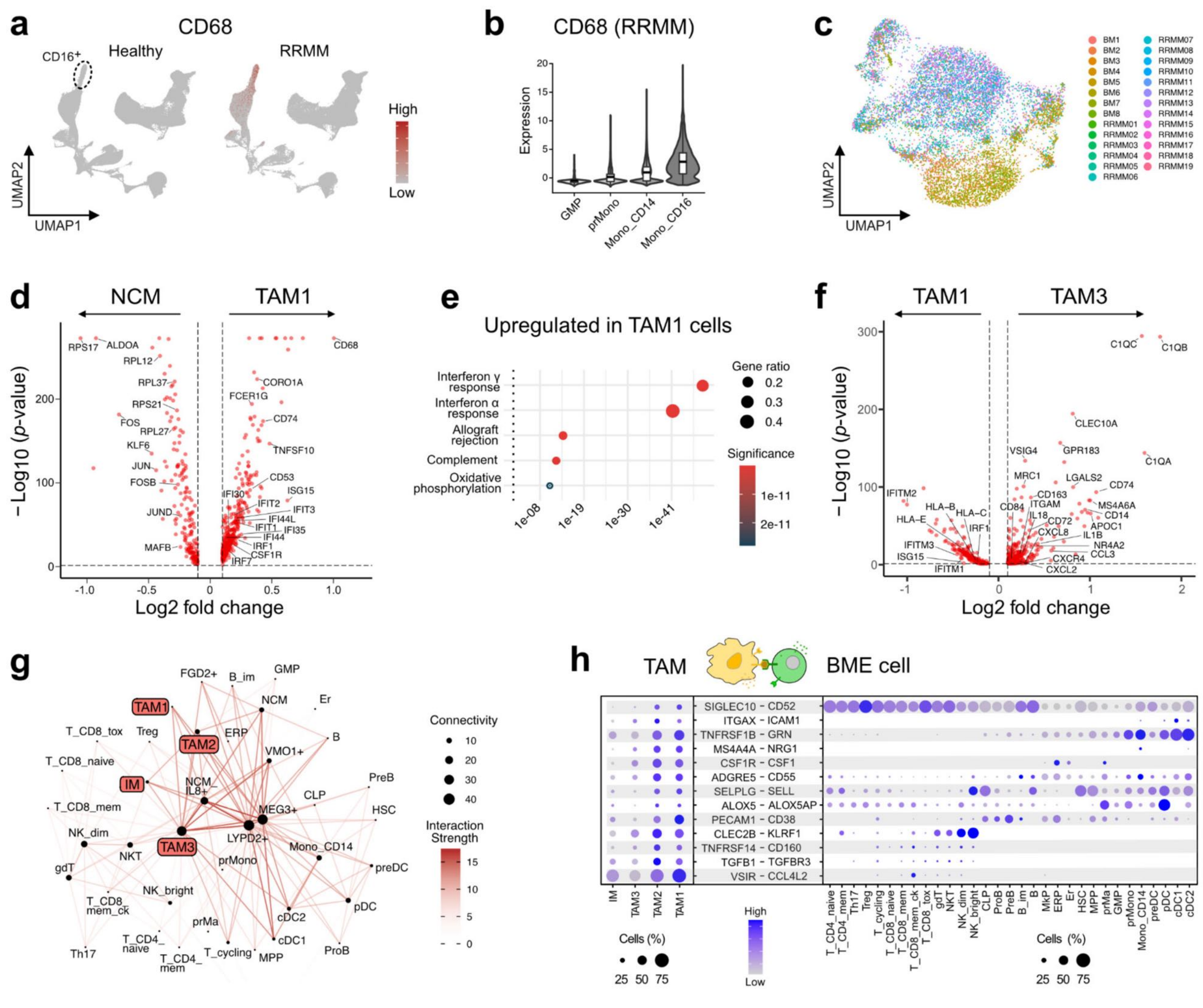

Supplementary Fig. 8. Analysis of CD16^{+} monocytes

(**a**) UMAP embedding of CD68 gene expression levels split in healthy donors and RRMM. (**b**) Violin plot of CD68 expression levels in the myelomonocytic lineage. Box plot: center line, median; box limits, first and third quartile; whiskers, minimum/maximum values. (**c**) UMAP embedding of subclustered CD16^{+} monocytes colored by patient/donor. (**d**) Volcano plot of differentially expressed between non-classical monocytes (NCM) and TAM1 subtypes. Thresholds for differential expression were *p*-value < 0.05 from a Bonferroni-adjusted Wilcoxon rank-sum test and logFC > 0.1 or < -0.1, respectively. (**e**) Gene set enrichment dot plot using hallmark gene sets of differentially upregulated genes in TAM1 cells vs. NCM. (**f**) Volcano plot of differentially expressed genes between TAM1 and TAM3 subtypes. Thresholds for differential expression were *p*-value < 0.05 from a Bonferroni-adjusted Wilcoxon rank-sum test and logFC > 0.1 or < -0.1, respectively. (**g**) Network plot of predicted cellular interactions between immune cell subsets in healthy donors. TAM/IM populations were assigned to the TAM1-3 populations in RRMM (Fig. 6) based on their transcriptome profile. These cell types were present at a much lower abundance in healthy donors as compared to the RRMM patients. In the network, every cell type is connected to its 4 top interacting cell types based on the sum of interaction strengths. The node size corresponds to the number of connected cell types and the coloring corresponds to the interaction strength. (**h**) Gene expression dot plot of ligand/receptors. RRMM-enriched CD16^{+} monocyte/macrophage subtypes (left) and associated interaction partners in the RRMM BME (right) are depicted. Only interactions are shown that were increased between TAM1/2 and immune cell types when compared to IM/TAM3 subtypes.

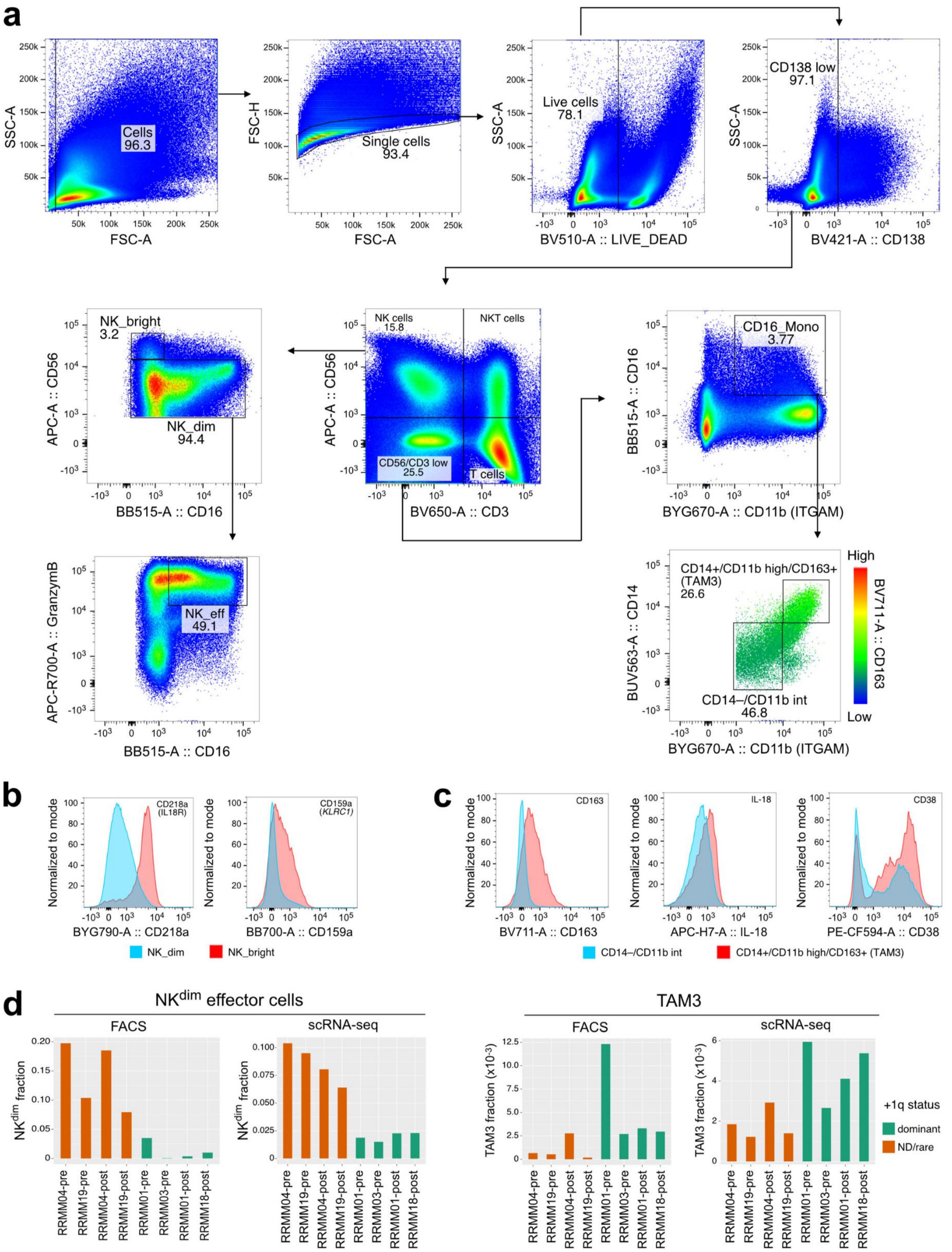

Supplementary Fig. 9. FACS validation of scRNA-seq data for NK and TAM3 cells

(**a**) FACS gating strategy for NK and TAM3 cells (eight samples combined). (**b**) FACS histograms for protein expression levels of CD218a (*IL18R1/IL18RAP*) and CD159 (*KLRC1*) for NK cells. (**c**) FACS histograms for protein expression levels of CD163, IL-18 and CD38 for CD16$^+$ monocyte/TAM populations. (**d**) Bar plot showing TAM3 (CD14$^+$/CD11b^{Hi}/CD163$^+$) and NK^{dim} effector cell fractions in individual samples derived from FACS analysis as depicted in panel *a*. CD138$^-$ cells represent the parental gate for calculating cell fractions, similar to the scRNA-seq data analysis.

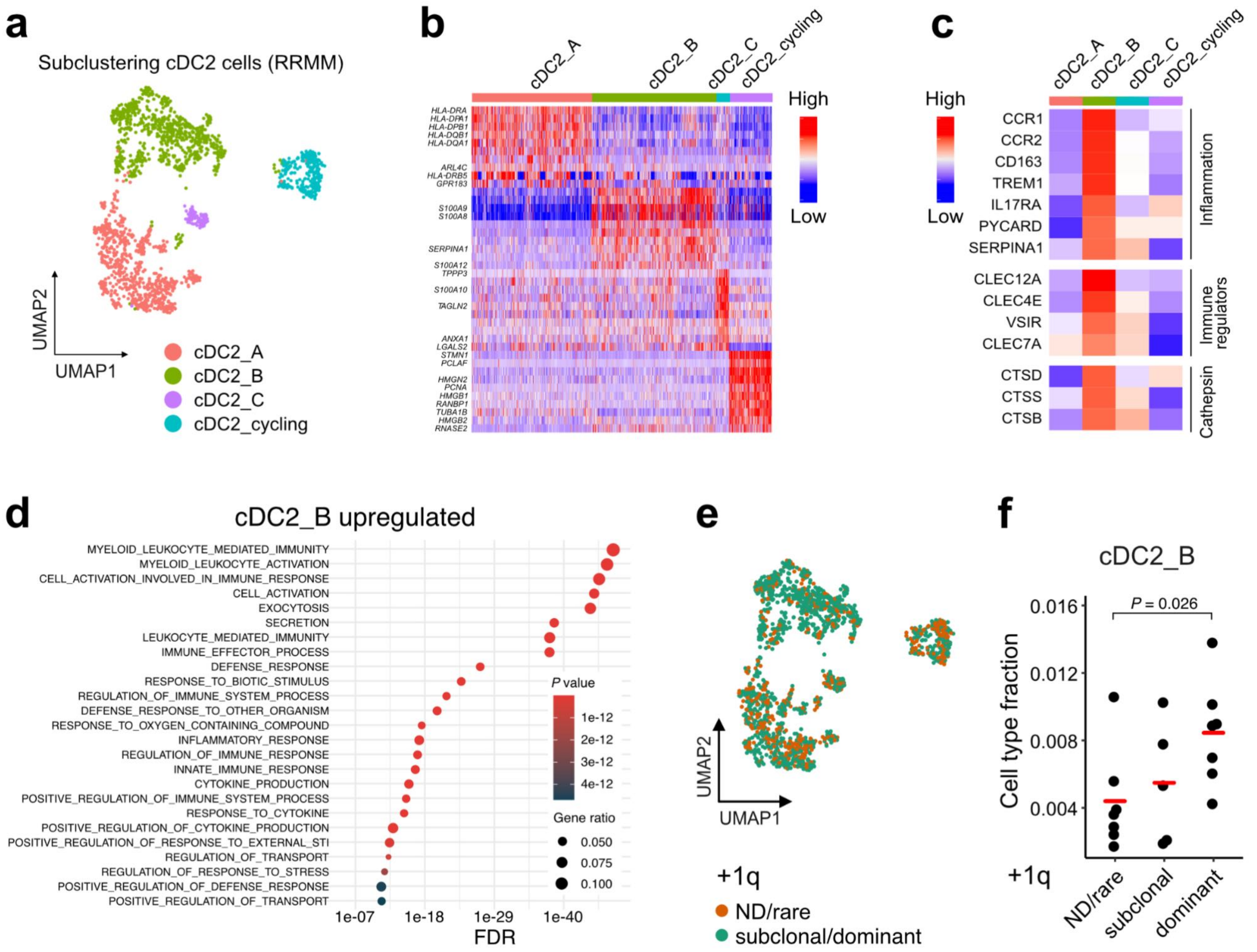

Supplementary Fig. 10. Characterization of inflammatory cDC2 population

(**a**) UMAP embedding of subclustered cDC2 cells colored by subtype. (**b**) Heatmap of top 10 genes that were differentially expressed in a given cDC2 subtype (*p*-value < 0.05 from a Bonferroni-adjusted two-sided Wilcoxon rank-sum test and logFC > 0.1 or < -0.1, respectively). (**c**) Heatmap showing average gene expression levels of selected genes that were upregulated in the inflammatory cDC2_B subtype (*p*-value < 0.05 from a Bonferroni-adjusted two-sided Wilcoxon rank-sum test and logFC > 0.1). (**d**) Gene set enrichment dot plot using GO gene sets of differentially upregulated genes in inflammatory cDC2_B subtype. (**e**) UMAP embedding of subclustered cDC2 cells colored by +1q status classified as ND/rare, subclonal or dominant. (**f**) Beeswarm plot for the comparison of inflammatory cDC2_B subtype fractions between the +1q groups ND/rare (*n* = 7 individuals), subclonal (*n* = 5 individuals) and dominant (*n* = 7 individuals). The Bonferroni-adjusted *p*-values from a two-sided Wilcoxon test are shown, and the red center line indicates the mean value.

Supplementary Tables

Supplementary Table 1. Overview of RRMM patients

Sample ID	Previous therapies	Group[a]	Drug (t1)[b]	Res-ponse[c]	Drug (t2)[b]	Res-ponse[c]	+1q State[d]
RRMM01	2	IMiD	Pom/Dex, anti-CD38	SD	–	–	high
RRMM02	3	NA	–	NA	–	–	high
RRMM03	2	MEKi	MEKi, BRAFi	PR	–	–	int
RRMM04	6	PI	Carfil/Dex	MR	–	–	low
RRMM05	1	NA	–	NA	–	–	low
RRMM06	3	IMiD	Len/Dex, anti-CD38	SD	MCL1i	PR	high
RRMM07	1	PI	Carfil/Dex, Cyclo-phosphamid	SD	–	–	low
RRMM08	6	NA	–	NA	–	–	int
RRMM09	3	MEKi	MEKi, BRAFi	CR	–	–	int
RRMM10	2	IMiD	Pom/Dex, HDACi	MR	–	–	high
RRMM11	4	IMiD	Pom/Dex, HDACi	SD	–	–	low
RRMM12	6	NA	–	NA	–	–	high
RRMM13	4	PI	Carfil/Dex	PR	–	–	int
RRMM14	3	IMiD	Pom/Dex	SD	–	–	low
RRMM15	3	IMiD	Pom/Dex, anti-SLAMF7	VGPR	–	–	high
RRMM16	3	MEKi	MEKi, BCL2i	CR	–	–	int
RRMM17	2	IMiD	Pom/Dex	PR	–	–	low
RRMM18	2	IMiD	Pom/Dex, Cyclo-phosphamid	SD	–	–	high
RRMM19	2	IMiD	Pom/Dex	PR	Carfil/Dex	VGPR	low
RRMM20	4	NA	–	NA	–	–	high

The RRMM patients studied here comprised 9 female and 11 male individuals with an average age of 61 years ranging from 43-68 years and a median of three prior lines treatment for 18/20 cases. Two patients were primary refractory to initial therapy (RRMM05 and RRMM07). For 15/20 patients paired samples before treatment and at relapse were acquired during either IMiD- (9), Carfilzomib- (3) or MEK inhibitor- (3) based therapies. A third time-point was analyzed after MCL1i- (RRMM06) and Carfilzomib- (RRMM19) based therapy, respectively. Treatment response was assessed by measuring monoclonal immunoglobulins and/or light chains in blood and urine in a central clinical lab.

[a] Treatment group. IMiD, immunmodulatory drugs; PI, proteasome inhibitor; MEKi, MEK inhibitor; MCL1i, MCL1 inhibitor; NA, not assessed.

[b] Pom, pomalidomide; Dex, dexamethasone, anti-CD38; Carfil, carfilzomib; BRAFi

[c] Best response. NA, not assessed; CR, complete response; VGPR, very good partial response; PR, partial response; MR, minimal response; SD, stable disease.

[d] Classification based on the InferCNV analysis into +1q low (<10%), intermediate (>10% and <80%) and high (>80%) according to the grouping shown in **Fig. 2J**.

Supplementary Table 2. Cell numbers of samples studied

Sample ID	Population	Cell number
RRMM01	PCs	1810
RRMM01	BME	4829
RRMM02	PCs	833
RRMM02	BME	2000
RRMM03	PCs	6227
RRMM03	BME	6927
RRMM04	PCs	839
RRMM04	BME	9395
RRMM05	PCs	10007
RRMM05	BME	11554
RRMM06	PCs	5277
RRMM06	BME	10925
RRMM07	PCs	1664
RRMM07	BME	6181
RRMM08	PCs	1027
RRMM08	BME	15116
RRMM09	PCs	2407
RRMM09	BME	3853
RRMM10	PCs	1932
RRMM10	BME	5575
RRMM11	PCs	3885
RRMM11	BME	3100
RRMM12	PCs	20193
RRMM12	BME	9644
RRMM13	PCs	932
RRMM13	BME	8663
RRMM14	PCs	1971
RRMM14	BME	4455
RRMM15	PCs	7309
RRMM15	BME	3049
RRMM16	PCs	2982
RRMM16	BME	5191
RRMM17	PCs	1112
RRMM17	BME	3462
RRMM18	PCs	6830
RRMM18	BME	11715
RRMM19	PCs	4644
RRMM19	BME	3569
RRMM20	PCs	1320
RRMM20	BME	not analyzed

Sample ID	Population	Cell number
BM1	PCs	118
BM1	BME	36129
BM2	PCs	425
BM2	BME	34224
BM3	PCs	275
BM3	BME	34101
BM4	PCs	312
BM4	BME	32780
BM5	PCs	185
BM5	BME	30917
BM6	PCs	288
BM6	BME	40684
BM7	PCs	547
BM7	BME	33176
BM8	PCs	181
BM8	BME	36819

Supplementary Table 3. Cell types and marker genes

Cell type	Abbreviation	Main marker gene(s) and features	Ref.
CD4$^+$ naive T-cell	T_CD4_naive	CD3D$^+$, CD40LG$^+$, CCR7$^+$, LEF1$^+$	4-6
CD4$^+$ memory T-cell	T_CD4_mem	CD3D$^+$, CD40LG$^+$, IL7R$^+$	4-6
Th17 T helper cell	Th17	CD3D$^+$, CD40LG$^+$, KLRB1$^+$	4-6
Regulatory T-cell	Treg	CD3D$^+$, CD40LG$^+$, IL2RA$^+$, FOXP3$^+$	4-6
Cycling T-cell	T_cycling	CD3D$^+$, MKI67$^+$, mostly S or G2/M phase	-
CD8$^+$ naïve T-cell	T_CD8_naive	CD3D$^+$, CD40LG$^+$, CCR7$^+$, LEF1$^+$	4-6
CD8$^+$ memory effector T-cell	T_CD8_mem	CD3D$^+$, CD8A$^+$, GZMK$^+$	4-6
CD8$^+$ high cytokine memory effector T-cell	T_CD8_ck	CD3D$^+$, CD8A$^+$, CCL3$^+$/CCL4$^+$, expressing high levels of cytokines IFNG, CCL3, or CCL4	4-6
CD8$^+$ cytotoxic T-cell	T_CD8_tox	CD3D$^+$, CD8A$^+$, GZMH$^+$	4-6
γδ T-cell	gdT	CD3D$^+$, CD3E$^+$, TRDC$^+$, TRGC1$^+$	7
NK T-cell	NKT	CD3D$^+$, CD56/NCAM1^{dim}, FCGR3A^{dim}, KLRC2$^+$	8
NK^{bright} cell	NK_bright	FCER1G$^+$, CD56/NCAM1$^+$, FCGR3A^{dim},	9
NK^{dim} cell	NK_dim	FCER1G$^+$, CD56/NCAM1^{dim}, CD16$^+$/FCGR3A$^+$	9
NK^{dim} activated cell	NK_act	CD69$^+$ subgroup of NK^{dim}	This study
NK^{dim} effector cell	NK_eff	NK^{dim}; GZMB^{high}, PRF1^{high}, CD16$^+$/FCGR3A^{high}	This study
Common lymphoid progenitor	CLP	IGLL1$^+$, ADA$^+$	10
Pro B-cell	ProB	IGLL1$^+$, VPREB1$^+$	10,11
Pre B-cell	PreB	IGLL1$^+$, CD24$^+$,	10,11
Immature B-cell	B_im	NEIL1$^+$, MS4A1$^+$	10,11
Mature B-cell	B	MS4A1$^+$	10,11
Plasma cell	PC	TNFRSF17$^+$	10,11
Megakaryocyte progenitor	MkP	PF4$^+$	10
Erythroid progenitor cell	ERP	SLC40A1$^+$	10
Erythroblast	Er	AHSP$^+$	10
Hematopoietic stem cell	HSC	AVP$^+$	10
Multi-potent progenitor	MPP	SPINK2$^+$	10
Mast-cell precursor	prMa	TPSB2$^+$	10
Granulocyte-monocyte progenitor	GMP	ELANE$^+$	10,12
Monocyte precursor	prMono	RETN$^+$	10,12
CD14$^+$ monocyte	Mono_CD14	CD14$^+$	10,12
CD16$^+$ monocyte	Mono_CD16	CD16$^+$/FCGR3A$^+$	10,12
Intermediate monocyte	IM	CD14$^+$, FCN1$^+$, VCAN$^+$, S100A8$^+$, VEGFA$^+$	This study
Tumor-associated macrophage 1	TAM1	CD68$^+$, IFN-response$^+$, MHC I^{Hi}	This study
Tumor-associated macrophage 2	TAM2	CD68$^+$, C1Q$^+$, IFN-response$^+$, MHC I^{Hi}	This study
Tumor-associated macrophage 3	TAM3	CD14$^+$, CD68$^+$, C1Q$^+$, MRC1$^+$, FOLR2$^+$, APOE$^+$, MHC II^{HI}	This study
Dendritic cell precursor	preDC	IGLL1$^+$	13,14
Plasmacytoid dendritic cell	pDC	CLEC4C$^+$	13,14
Conventional dendritic cell 1	cDC1	CLEC9A$^+$	13,14
Conventional dendritic cell 2	cDC2	CLEC10A$^+$	13,14

Supplementary Table 4. Gene expression signatures

Signature	Genes	Ref.
T-cell dysfunction/ exhaustion	PDCD1, CTLA4, TIGIT, LAG3, HAVCR2, CD244, VSIR	[5,8,15]
Effectorness	PRF1, GZMA, GNLY, GZMH, GZMK, NKG7	[4-6]
IFNG-response	HALLMARK IFNG-response	[16]
Ribosome	KEGG-Ribosome	[17]
TNFα-signaling	HALLMARK_TNFA_SIGNALING_VIA_NFKB	[16]
IL1-signaling	PID_IL1_PATHWAY	[17]
IL2-signaling	HALLMARK_IL2_STAT5_SIGNALING	[16]
IL6-signaling	HALLMARK_IL6_JAK_STAT3_SIGNALING	[16]
Inflammatory response	HALLMARK_INFLAMMATORY_RESPONSE	[16]
TGFβ-signaling	HALLMARK_TGF_BETA-SIGNALING	[16]
KLF6 target genes	ASAH1, ATF3, CDH1, CDKN1A, CERS2, CGB5, DAPK2, IGF1R, KRT12, LAMA1, LTC4S, NOS2, PMAIP1, PSG3, PSG5, TFPI2, TXNIP)	[18]
1q gain (+1q) [a]	PMVK, ILF2, CCT3, TIMM17A, LAMTOR2, SNRPE, TMCO1, PSMB4, PSMD4, COX20, HAX1, KRTCAP2, EPRS, PARP1, SDHC, AC245014.3, NAXE, UFC1, ARPC5, RBM8A, H3F3A, IFI16, TAGLN2, MRPL55, ARF1, MRPS21, DPM3, ADAR, C1orf43, RGS1, UAP1, MPC2, GUK1, TSTD1, NENF, MGST3, SSR2, PFDN2, JTB, HIST2H2BE, HNRNPU, BTG2, MDM4, B4GALT3, TOMM20, CTSS, LMNA, RPS27, RGS2, SLAMF7, MCL1	This study

[a] The +1q gene expression signature comprised 51 genes and included known drivers of multiple myeloma pathogenesis that have already been linked to +1q such as *ILF2* [19], *ADAR* [20] and again *MCL1* [21]. Furthermore, we detected several genes that so far have not been associated with +1q multiple myeloma: *SLAMF7,* a primary drug target in multiple myeloma [22]; *PARP1* (encoding for poly[ADP-ribose] polymerase), an enzyme involved in DNA repair and promising drug target in BRCA-defective tumors [23]; the regulator of G-protein signaling *RGS1* whose expression is associated with poor prognosis in multiple myeloma [24]; and *CTSS* (Cathepsin S), a cysteine protease involved in the recruitment of immunosuppressive myeloid cells [25,26]

Supplementary Table 5. Data analysis software

Software	Ref.	Link
ACEseq	27	https://aceseq.readthedocs.io/
Bowtie	28	bowtie-bio.sourceforge.net/bowtie2/index.shtml
Bioconductor R[a]	29	www.bioconductor.org
CellPhoneDB	30	https://www.cellphonedb.org
ComplexHeatmap	31	https://www.bioconductor.org/packages/release/bioc/html/ComplexHeatmap.html
Custom scripts and pipelines for this study	32	https://github.com/RippeLab/RRMM
Cytoscape	33	cytoscape.org
DAVID	34	david.ncifcrf.gov
dendextend	35	https://github.com/talgalili/dendextend
DESeq2	1	doi.org/10.18129/B9.bioc.DESeq2
EnhancedVolcano	36	github.com/kevinblighe/EnhancedVolcano
ggpointdensity		github.com/LKremer/ggpointdensity
Harmony	37	https://github.com/immunogenomics/harmony
hypeR	38	github.com/montilab/hypeR
inferCNV	39	github.com/broadinstitute/inferCNV
Nextflow[b]	40	https://github.com/nextflow-io/nextflow
nf-core (bulk RNA-seq)	41	https://nf-co.re, https://github.com/nf-core/rnaseq
OTP WGS pipeline	42	https://otp.dkfz.de/otp/
presto		https://github.com/immunogenomics/presto
schex		github.com/SaskiaFreytag/schex
Scrublet	43	https://github.com/AllonKleinLab/scrublet
SCTransform	44	https://github.com/ChristophH/sctransform
Seurat	45	https://satijalab.org/seurat/
SingleR	46	https://github.com/LTLA/SingleR
STAR	47	github.com/alexdobin/STAR
UMAP	48	https://github.com/lmcinnes/umap/archive/0.2.4.tar.gz

[a] Visualization of data was conducted with the R packages ggplot2 and igraph.

[b] Initial quality control including removal of low-quality libraries, doublet detection and celltype prediction with SingleR, single cell copy number calling (InferCNV) and prediction of cellular interactions (CellphoneDB) were implemented in Nextflow for automated processing of data sets.

Supplementary Table 6. Antibodies for fluorescence activated cell sorting

Antibody target	Fluoro-chrome	Clone	Dilution Factor	Order number	Company
CD56	APC	NCAM16.2	1:40	341025	Becton Dickinson
CD16	FITC	3G8	1:10	560996	Becton Dickinson
CD38	PE-CF594	HIT2	1:40	562288	Becton Dickinson
CD3	BV650	SK7	1:40	563999	Becton Dickinson
CD3	APC-R700	UCHT1	1:75	565119	Becton Dickinson
LAG-3	PE	T47-530	1:30	565616	Becton Dickinson
CD138	BV421	MI15	1:40	565943	Becton Dickinson
PD-1	BV480	EH12.1	1:30	566112	Becton Dickinson)
GranzymeB	R718	GB11	1:40	566964	Becton Dickinson
CD45	BUV805	HI30	1:40	612891	Becton Dickinson
CD45	APC-H7	2D1	1:50	641417	Becton Dickinson
TCRγ/δ	PE-Cy7	11F2	1:30	655410	Becton Dickinson
CD14	BUV563	M5E2	1:40	741360	Becton Dickinson
TIGIT	BV421	741182	1:30	747844	Becton Dickinson
CD8	APC	RPA-T8	1:30	555369	Becton Dickinson
CD159a	BB700	131411	1:40	747926	Becton Dickinson
KLRG-1	BV605	2F1/KLRG1	1:30	138419	Biolegend
CD11b	PE-Cy5	ICRF44	1:40	301308	Biolegend
CD218a	PE-Cy7	H44	1:40	313812	Biolegend
CD206	PE	15-2	1:40	321105	Biolegend
CD163	BV711	GHI/61	1:40	333630	Biolegend
IL18	Alexa Fluor 750	74801	1:20	IC646S-100µg	R&D Systems

Supplementary Table 7. Inventory of supplementary data sets

Data set	File name	Figure/ Table ref.	Description
Supplementary Data Set 1	Sup_Data_01.xlsx	Fig. 1, Supplementary Fig. 1, Supplementary Table 1	Sample meta data with sample IDs and additional sample information.
Supplementary Data Set 2	Sup_Data_02.xlsx	Fig. 2, Supplementary Fig. 2, Supplementary Table 4	Annotation of upregulated genes of the +1q signature

These data sets are provided as separate files in Microsoft Excel format.

References

1. Love, M.I., Huber, W. & Anders, S. Moderated estimation of fold change and dispersion for RNA-seq data with DESeq2. Genome Biol 15, 550 (2014).
2. Seckinger, A. et al. Target Expression, Generation, Preclinical Activity, and Pharmacokinetics of the BCMA-T Cell Bispecific Antibody EM801 for Multiple Myeloma Treatment. Cancer Cell 31, 396-410 (2017).
3. Ledergor, G. et al. Single cell dissection of plasma cell heterogeneity in symptomatic and asymptomatic myeloma. Nat Med 24, 1867-1876 (2018).
4. Park, J.E. et al. A cell atlas of human thymic development defines T cell repertoire formation. Science 367(2020).
5. Szabo, P.A. et al. Single-cell transcriptomics of human T cells reveals tissue and activation signatures in health and disease. Nat Commun 10, 4706 (2019).
6. van der Leun, A.M., Thommen, D.S. & Schumacher, T.N. CD8(+) T cell states in human cancer: insights from single-cell analysis. Nat Rev Cancer 20, 218-232 (2020).
7. Pizzolato, G. et al. Single-cell RNA sequencing unveils the shared and the distinct cytotoxic hallmarks of human TCRVdelta1 and TCRVdelta2 gammadelta T lymphocytes. Proc Natl Acad Sci U S A 116, 11906-11915 (2019).
8. Johnston, R.J. et al. VISTA is an acidic pH-selective ligand for PSGL-1. Nature 574, 565-570 (2019).
9. Stabile, H., Fionda, C., Gismondi, A. & Santoni, A. Role of Distinct Natural Killer Cell Subsets in Anticancer Response. Front Immunol 8, 293 (2017).
10. Hay, S.B., Ferchen, K., Chetal, K., Grimes, H.L. & Salomonis, N. The Human Cell Atlas bone marrow single-cell interactive web portal. Exp Hematol 68, 51-61 (2018).
11. Bendall, S.C. et al. Single-cell trajectory detection uncovers progression and regulatory coordination in human B cell development. Cell 157, 714-25 (2014).
12. van Galen, P. et al. Single-Cell RNA-Seq Reveals AML Hierarchies Relevant to Disease Progression and Immunity. Cell 176, 1265-1281 e24 (2019).
13. Collin, M. & Bigley, V. Human dendritic cell subsets: an update. Immunology 154, 3-20 (2018).
14. Merad, M., Sathe, P., Helft, J., Miller, J. & Mortha, A. The dendritic cell lineage: ontogeny and function of dendritic cells and their subsets in the steady state and the inflamed setting. Annu Rev Immunol 31, 563-604 (2013).
15. Li, H. et al. Dysfunctional CD8 T Cells Form a Proliferative, Dynamically Regulated Compartment within Human Melanoma. Cell 176, 775-789 e18 (2019).
16. Liberzon, A. et al. The Molecular Signatures Database (MSigDB) hallmark gene set collection. Cell Syst 1, 417-425 (2015).
17. Subramanian, A. et al. Gene set enrichment analysis: a knowledge-based approach for interpreting genome-wide expression profiles. Proc Natl Acad Sci U S A 102, 15545-50 (2005).
18. Han, H. et al. TRRUST v2: an expanded reference database of human and mouse transcriptional regulatory interactions. Nucleic Acids Res 46, D380-D386 (2018).
19. Samo, A.A. et al. MCL1 gene co-expression module stratifies multiple myeloma and predicts response to proteasome inhibitor-based therapy. Genes Chromosomes Cancer 57, 420-429 (2018).
20. Lazzari, E. et al. Alu-dependent RNA editing of GLI1 promotes malignant regeneration in multiple myeloma. Nat Commun 8, 1922 (2017).
21. Slomp, A. et al. Multiple myeloma with 1q21 amplification is highly sensitive to MCL-1 targeting. Blood Adv 3, 4202-4214 (2019).
22. van de Donk, N.W. et al. Clinical efficacy and management of monoclonal antibodies targeting CD38 and SLAMF7 in multiple myeloma. Blood 127, 681-95 (2016).
23. Rouleau, M., Patel, A., Hendzel, M.J., Kaufmann, S.H. & Poirier, G.G. PARP inhibition: PARP1 and beyond. Nat Rev Cancer 10, 293-301 (2010).

24. Roh, J. et al. RGS1 expression is associated with poor prognosis in multiple myeloma. J Clin Pathol 70, 202-207 (2017).
25. Yang, M. et al. Cathepsin S-mediated autophagic flux in tumor-associated macrophages accelerate tumor development by promoting M2 polarization. Mol Cancer 13, 43 (2014).
26. Wilkinson, R.D. et al. CCL2 is transcriptionally controlled by the lysosomal protease cathepsin S in a CD74-dependent manner. Oncotarget 6, 29725-39 (2015).
27. Giessler, K.M. et al. Genetic subclone architecture of tumor clone-initiating cells in colorectal cancer. J Exp Med 214, 2073-2088 (2017).
28. Langmead, B., Trapnell, C., Pop, M. & Salzberg, S.L. Ultrafast and memory-efficient alignment of short DNA sequences to the human genome. Genome Biol 10, R25 (2009).
29. Gentleman, R.C. et al. Bioconductor: open software development for computational biology and bioinformatics. Genome Biol 5, R80 (2004).
30. Efremova, M., Vento-Tormo, M., Teichmann, S.A. & Vento-Tormo, R. CellPhoneDB: inferring cell-cell communication from combined expression of multi-subunit ligand-receptor complexes. Nat Protoc 15, 1484-1506 (2020).
31. Gu, Z., Eils, R. & Schlesner, M. Complex heatmaps reveal patterns and correlations in multidimensional genomic data. Bioinformatics 32, 2847-9 (2016).
32. Steiger, S., Tirier, S.M. & Rippe, K. Nextflow data processing pipelines and R code for single cell RNA sequencing analysis of relapsed and refractory multiple myeloma. Zenodo, https://doi.org/10.5281/zenodo.5532552 (2021).
33. Shannon, P. et al. Cytoscape: a software environment for integrated models of biomolecular interaction networks. Genome Res 13, 2498-504 (2003).
34. Huang da, W., Sherman, B.T. & Lempicki, R.A. Systematic and integrative analysis of large gene lists using DAVID bioinformatics resources. Nat Protoc 4, 44-57 (2009).
35. Galili, T. dendextend: an R package for visualizing, adjusting and comparing trees of hierarchical clustering. Bioinformatics 31, 3718-20 (2015).
36. Blighe, K., Rana, S. & Lewis, M. EnhancedVolcano: Publication-ready volcano plots with enhanced colouring and labeling. Github, https://github.com/kevinblighe/EnhancedVolcano (2018).
37. Korsunsky, I. et al. Fast, sensitive and accurate integration of single-cell data with Harmony. Nat Methods 16, 1289-1296 (2019).
38. Federico, A. & Monti, S. hypeR: an R package for geneset enrichment workflows. Bioinformatics 36, 1307-1308 (2020).
39. Tickle, T., Tirosh, I., Georgescu, C., Brown, M. & Haas, B. inferCNV of the Trinity CTAT Project. Github, https://github.com/broadinstitute/inferCNV. (2019).
40. Di Tommaso, P. et al. Nextflow enables reproducible computational workflows. Nat Biotechnol 35, 316-319 (2017).
41. Ewels, P.A. et al. The nf-core framework for community-curated bioinformatics pipelines. Nat Biotechnol 38, 276-278 (2020).
42. Reisinger, E. et al. OTP: An automatized system for managing and processing NGS data. J Biotechnol 261, 53-62 (2017).
43. Wolock, S.L., Lopez, R. & Klein, A.M. Scrublet: Computational Identification of Cell Doublets in Single-Cell Transcriptomic Data. Cell Syst 8, 281-291 e9 (2019).
44. Hafemeister, C. & Satija, R. Normalization and variance stabilization of single-cell RNA-seq data using regularized negative binomial regression. Genome Biol 20, 296 (2019).
45. Stuart, T. et al. Comprehensive Integration of Single-Cell Data. Cell 177, 1888-1902 e21 (2019).
46. Aran, D. et al. Reference-based analysis of lung single-cell sequencing reveals a transitional profibrotic macrophage. Nat Immunol 20, 163-172 (2019).
47. Dobin, A. et al. STAR: ultrafast universal RNA-seq aligner. Bioinformatics 29, 15-21 (2013).
48. Becht, E. et al. Dimensionality reduction for visualizing single-cell data using UMAP. Nat Biotechnol 37, 38-44 (2019).
