# Supplementary material for: Subclone-specific microenvironmental impact and drug response in refractory multiple myeloma revealed by single‐cell transcriptomics
Source: Nat Commun. 2021 Nov 29;12:6960. doi: 10.1038/s41467-021-26951-z (PMC8630108; doi:10.1038/s41467-021-26951-z)
Supplement: Supplementary file 7 — Reply to requested editorial changes. [file 41467_2021_26951_MOESM7_ESM.docx]

Your manuscript has been checked for clarity and against journal policies and formatting style. The issues listed below must be addressed; failure to do so will cause delays in acceptance.

For further information, please see our [formatting instructions](https://www.nature.com/documents/ncomms-formatting-instructions.pdf).

Please highlight all changes in the manuscript text file, either using the track changes feature in Microsoft Word or coloured highlighting in LaTeX.

Please include your response to these requests in the space provided and return this checklist with your final submission.

| **EDITORIAL REQUESTS:** | | | **AUTHOR RESPONSE:** |
| --- | --- | --- | --- |
| **POLICIES AND CHECKLISTS** | | | **POLICIES AND CHECKLISTS** |
| An updated editorial policy checklist must be completed and uploaded as a related manuscript file with the revised manuscript. All points on the policy checklist must be addressed; if needed, please revise your manuscript in response to these points. Please note that this form is a dynamic 'smart pdf' and must therefore be downloaded and completed in Adobe Reader, instead of opening it in a web browser. https://www.nature.com/authors/policies/Policy.pdf | | | **✓** |
| Please find attached a reporting summary that includes comments on how to revise it in line with our policies and requests the addition of further information in the text. An updated reporting summary must be completed and uploaded as a supplementary information file with the revised manuscript. This checklist is published alongside your manuscript online. Please note that this form is a dynamic 'smart pdf' and must therefore be downloaded and completed in Adobe Reader, instead of opening it in a web browser. https://www.nature.com/authors/policies/ReportingSummary.pdf | | | An updated version of the reporting summary was uploaded together with the revised manuscript. |
| Please also find below a list of comments requesting additional information in the figure legends, text, and methods section to comply with our reporting policies. | | |  |
| **TITLE PAGE (page 2 of our formatting instructions)** | | | **TITLE PAGE (page 2 of our formatting instructions)** |
| When discussing the current work in the abstract, please use the present tense. | | | **✓** |
| **MAIN TEXT (pages 1 to 3 of our formatting instructions)** | | | **MAIN TEXT (pages 1 to 3 of our formatting instructions)** |
| The final paragraph of the Introduction must begin with a phrase like “In this work” or “Here, we show”, and contain a brief summary of the major results and conclusions of the current work, written in the present tense. | | | **✓** |
| **LANGUAGE AND STYLE (page 6 of our formatting instructions)** | | | **LANGUAGE AND STYLE (page 6 of our formatting instructions)** |
| Please remove phrases such as 'new', 'novel', 'for the first time', 'unprecedented', etc., as novelty is clear from the context. Please also remove exaggerated language such as 'extremely', 'outstanding', etc. | | | **✓** |
| Please do not use italics, bold font, underlining or speech marks unless required for technical terms (in both the main text and the display items). | | | **✓** |
| Please use italics for gene names, and roman font for protein names. This applies to both the main text and display items. | | | **✓** |
| Please make sure that mathematical terms throughout your manuscript and Supplementary Information (including in figures, figure axes, and legends) conform strictly to the following guidelines. Equations must be supplied in editable format, and not as images. Scalar variables (e.g. x, V, χ) must be typeset in italic, whereas multi-letter variables and functions (e.g. log) must be formatted in roman. Vectors (such as the wavevector k or the magnetic field vector B) must be typeset in bold without italics. | | | **✓** |
| **METHODS AND DATA (page 3 of our formatting instructions)** | | | **METHODS AND DATA (page 3 of our formatting instructions)** |
| Sufficient details of the experiments must be provided in the Methods section such that they could be reproduced without reference to published papers. Use of the term 'as described previously' is not encouraged. | | | **✓** |
| Please confirm that you have complied with all relevant ethical regulations for work with human participants, and that informed consent was obtained. Please state this in the Methods section, including the name of the board and institution that approved the study protocol. | | | **✓**  It is stated in the Methods section that all patients provided written informed consent before participating in the study, and that approval was obtained by the ethics committee of the Medical Faculty at the University of Heidelberg. |
| Please include a statement explaining whether you have obtained consent to publish information that identifies individuals, including indirect identifiers such as Patient IDs and gender. If not, please amend the information so that individuals can no longer be identified (for example, by providing data in aggregate, or averages where suitable, for each of the categories). If this is not possible, please remove this data from the manuscript. | | | **✓**  No information that could identify individuals directly or indirectly is given in the revised manuscript. |
| Please provide a Supplementary Figure to graphically account for all FACS sequential gating/sorting strategies, or provide gating/sorting strategies in-figure. If the former, please be sure to indicate, in the Supplementary Figure legend, which gating panel(s) correspond to which FACS data panel(s) in the manuscript figures. Please refer to this published <i>Nature Communications</i> article as an example: Supplementary Fig. 2 of Nat Commun. 2017 Apr 21;8:15067. doi: 10.1038/ncomms15067. | | | **✓**  FACS gating strategies are described in Supplementary Fig. 7d and Supplementary Fig. 9a. |
| All published manuscripts reporting original research in Nature Research journals must include a data availability statement, as a separate section before the References and under the heading 'Data Availability'. The data availability statement must make the conditions of access to the “minimum dataset” that are necessary to interpret, verify and extend the research in the article, transparent to readers. This minimum dataset may be provided through deposition in public community/discipline-specific repositories, custom proprietary repositories or general repositories like Figshare, Zenodo and Dryad. Providing large datasets in supplementary information is strongly discouraged and the preferred approach is to make data available in repositories. Scientific Data, a Nature Research journal, maintains a list of approved and recommended data repositories to support researchers seeking suitable repositories for their data (https://www.nature.com/sdata/policies/repositories). Please refer to our authorship policy for information about authors’ responsibilities for preserving and making available data, code and materials upon publication. Authors are responsible for obtaining all necessary permissions and ensuring compliance with local regulatory requirements for data sharing. The Data Availability Statement should also reference any source data published alongside the paper. If DOIs are provided, we also strongly encourage including these in the Reference list (authors, title, publisher (repository name), identifier, year). For clinical datasets or third party data, please ensure that the statement adheres to our policy (https://www.nature.com/nature-research/editorial-policies/reporting-standards#availability-of-data) | | | **✓**  See Data Availability section of the manuscript. |
| Please use the following template to provide all the information stated above: The XX data generated in this study have been deposited in the YY database under accession code ZZ [add hyperlink here]. The XX data are available under restricted access for {insert reason}, access can be obtained by {explain how}. The raw XX data are protected and are not available due to data privacy laws. The processed XX data are available at YY. The XX data generated in this study are provided in the Supplementary Information/Source Data file. The XX data used in this study are available in the YY database under accession code ZZ [Add hyperlink here]. | | | **✓**  The Data Availability section now follows this template. |
| In particular, please ensure that your processed data under accession number GSE161801 is publicly released by the time you submit your revised manuscript. In your Data Availability statement, please provide direct hyperlinks to your accession numbers, and wrap them in square brackets. Please also indicate whether your data are available under restricted access (especially the raw data on EGA), as well as the conditions, procedure and further details to request access to this data. Please also provide the accession numbers and hyperlinks to any published data that you used in your manuscript together with information about restrictions and access in the Data Availability statement. We were not able to retrieve the dataset under accession code EGAS00001004363; please ensure that a valid accession number and hyperlink are provided. | | | **✓**  The processed data under accession number GSE161801 have been publicly released. The WGS data that were previously referenced to EGAS00001004363 have now been included together with the primary scRNA-seq reads at EGAS00001004805. Thus, the reference to the yet not public data set at EGAS00001004363 is no longer needed and has been deleted. |
| Nature Research policies (https://go.nature.com/data-availability-AIP) strongly encourage deposition of research data in public repositories. In some cases this is mandatory, and you may have been previously advised if that was the case. If you need help depositing and curating your research data you should consider: - Contacting Springer Nature’s Research Data Helpdesk (https://go.nature.com/helpdesk-AIP) for advice - Finding a suitable data repository (https://go.nature.com/RD-policies-AIP) for your data Please provide a unique identifier for the data (for example a DOI or a permanent URL) in the data availability statement, if possible. If the repository does not provide identifiers, we encourage authors to supply the search terms that will return the data. For data that have been obtained from publicly available sources, please provide a URL and the specific data product name in the data availability statement. Data with a DOI should be included in the reference list and cited where relevant. Alternatively, include the data in the Supplementary Information. For datasets for which mandatory deposition is not required and the data can only be shared on request, please explain why in your Data Availability Statement and in your response here.  Please refer to our data policies here: http://www.nature.com/authors/policies/availability.html | | | **✓**  See Data Availability section. |
| We notice that you have deposited your code in a Github repository, which we fully support. We strongly encourage you in addition to make your code citable by obtaining a DOI for the Github repository in order to provide a permanent reference to the version of the code used in this study and improve reproducibility. This can be done by linking the repository to Zenodo, following the instructions here: https://guides.github.com/activities/citable-code/ Please cite the Github repository in your manuscript text or Code Availability statement and in your reference list: authors, title (this paper), repository name, DOI identifier, year. Alternatively, you can deposit the code in Gigantum or Code Ocean for the same purpose. | | | **✓**  The Github repository has now been linked to a repository at Zenodo at <https://doi.org/10.5281/zenodo.5532552>. The latter reference is now cited with this doi in the manuscript. |
| All accession codes must be accompanied with their hyperlinks throughout (for example, "5XRN [http://doi.org/10.2210/pdb5XRN/pdb]", "1483958 [https://doi.org/10.5517/ccdc.csd.cc1lt5m6]", "SRP109982 [https://www.ncbi.nlm.nih.gov/sra/?term=SRP109982]", "GSE101099 [https://www.ncbi.nlm.nih.gov/geo/query/acc.cgi?acc=GSE101099]" or "NQLW00000000 [https://www.ncbi.nlm.nih.gov/assembly/GCA_002312845.1/]"). | | | **✓**  See Data Availability section. |
| A reference to the source data file should be added in the 'Data Availability' section, using the text “Source data are provided with this paper.” | | | **✓**  Source data are now provided as an Excel file. |
| **END NOTES (pages 3 and 4 of our formatting instructions)** | | | **END NOTES (pages 3 and 4 of our formatting instructions)** |
| Please provide a 'Competing Interests' section after the 'Author Contributions' section that refers to all authors and declares both financial and non-financial interests. If there are no competing interests, please add the statement "The authors declare no competing interests." For more information, please see https://www.nature.com/nature-research/editorial-policies/competing-interests | | | **✓** |
| **DISPLAY ITEMS (pages 4 and 5 of our formatting instructions)** | | | **DISPLAY ITEMS (pages 4 and 5 of our formatting instructions)** |
| The use or adaptation of previously published images is strongly discouraged. If this is unavoidable, please request the necessary rights documentation to re-use such material from the relevant copyright holders and return this to us when you submit your revised manuscript. Please check whether your manuscript or Supplementary Information contain third-party images, such as figures from the literature, stock photos, clip art or commercial satellite and map data. | | | **✓** |
| In particular, please indicate whether you or a co-author created figures 1B, 7F. | | | **✓**  We replaced the image in Fig. 1b. All figures (including the new Fig. 1b and Fig. 7f) in the revised manuscript were created by the authors (SMT, SS or KR). |
| Any abbreviations, symbols or colours present in your figures must be defined in the associated legends. | | | **✓** |
| All colour scales must be defined and intensity levels must be provided in either the figure or its associated legend. | | | **✓** |
| **SUPPLEMENTARY INFORMATION (page 5 of our formatting instructions)** | | | **SUPPLEMENTARY INFORMATION (page 5 of our formatting instructions)** |
| We do not edit Supplementary Information files; they will be uploaded with the published article as they are submitted with the final version of your manuscript. Any tracked changes should be removed from the file and the file should be provided as a PDF file. Supplementary Figures do not need to be provided separately. | | | **✓** |
| All Supplementary Information items (e.g. Supplementary Figures, Supplementary Tables, Supplementary Methods, Supplementary Notes, Supplementary Discussion, Supplementary References) must be included in one PDF document. Only Supplementary Movie, Audio, Data and Software files should be submitted separately from the Supplementary Information. In addition, please refer to Supplementary Figures, Tables, etc. as Supplementary Figure 1 instead of Figure S1. | | | **✓** |
| Supplementary References should appear at the end of the Supplementary Information file, and must be self-contained and numbered from 1. References mentioned in both the main text and the Supplementary Information should be part of both reference lists so that the Supplementary Information does not refer to the reference list in the main paper and vice versa. | | | **✓** |
| Please ensure that a Source Data file is included with your resubmission. Within the Source Data file, the relevant raw data from each figure or table (in the main manuscript and in the Supplementary Information) should be represented by a single sheet in an Excel document, or a single .txt file or other file type in a zipped folder. Uncropped blots and gel images should be pasted in and labelled with the relevant panel and identifying information such as the antibody used. An example of the Source Data file is available demonstrating the correct format: https://www.nature.com/documents/ncomms-example-source-data.xlsx The file should be labelled 'Source Data', with the title and a brief description included in your response here, and should be mentioned in all relevant figure legends using the template text below: "Source data are provided as a Source Data file." | | | **✓**  A Source Data Excel file is provided. For some plots this is not possible to include the source data in the xls file due to size or data format (e.g., UMAP plots that have up to 500,000 data points). For these cases we provide R-scripts and a description on how to regenerate the plots at the Github/Zenodo repository with the publicly available processed data at GSE161801. This is described with the corresponding links at the beginning of the xls Source Data file. |
| **PUBLICATION** | | | **PUBLICATION** |
| Your paper will be accompanied by a two-sentence Editor's summary, of between 250-300 characters including spaces, when it is published online. I have drafted the summary below. If you would like to make changes to this, please provide me with a suitably edited version.  "Relapsed/refractory multiple myeloma (RRMM) is a heterogeneous disease with high drug resistance. Here the authors analyse RRMM samples with single-cell RNA-sequencing, revealing a prognostic copy-number gain in chromosome 1q and the impact of therapy and tumour progression on the microenvironment." | | | Thank you, we suggest some minor changes: "Relapsed/refractory multiple myeloma (RRMM) is characterized by a remarkable heterogeneity and high drug resistance. Here, the authors analyse RRMM samples by single-cell RNA-sequencing, revealing molecular features associated with high-risk chromosomal 1q-gain and changes in the tumor microenvironment." |
| As part of our efforts to communicate our content to a wider audience, we endeavour to highlight papers published in Nature Communications on the journal’s Twitter account (https://twitter.com/NatureComms). If you would like us to mention authors, institutions or lab groups in these tweets, please provide the relevant twitter handles. | | | @SMTirier, @KarstenRippe |
|  | **EDITORIAL REQUESTS:** | **AUTHOR RESPONSE:** | |
| **1.** | **Data presentation:** Please ensure that data presented in a plot, chart or other visual representation format shows data distribution clearly (e.g. dot plots, box-and-whisker plots). When using bar charts, please overlay the corresponding data points (as dot plots) whenever possible and always for n ≤ 10. (Please see the following editorial for the rationale behind this request and an example <https://www.nature.com/articles/s41551-017-0079>). | | |
| **2.** | **Statistics**:Wherever statistics have been derived (e.g. error bars, box plots, statistical significance) the legend needs to provide and define the n number (i.e. the sample size used to derive statistics) as a precise value (not a range), using the wording “n=X biologically independent samples/animals/cells/independent experiments/n= X cells examined over Y independent experiments” etc. as applicable. | | |
|  | **Legends requiring revision:**   1. Please note that this information is missing in the legends of figures: 2k, 5h, 6e, 7a, 7e; Supplementary figures: 1b, 1e-g, 3h-i, 4b, 4e, 5a, 10f. | We added the required information to the respective figure legends. In Supplementary Fig. 1, we refer to Supplementary Table 2 and Supplementary Data Set 1, which provide cell numbers for all samples. | |
| **3.** | Please note that statistics such as error bars significance and p values cannot be derived from n<3 and must be removed from all such cases. | | |
|  | We strongly discourage deriving statistics from technical replicates, unless there is a clear scientific justification for why providing this information is important. Conflating technical and biological variability, e.g., by pooling technically replicates samples across independent experiments is strongly discouraged. (For examples of expected description of statistics in figure legends, please see the following <https://www.nature.com/articles/s41467-019-11636-5> or <https://www.nature.com/articles/s41467-019-11510-4>). | | |
|  | All error bars need to be defined in the legends (e.g. SD, SEM) together with a measure of centre (e.g. mean, median). For example, the legends should state something along the lines of “Data are presented as mean values +/- SEM” as appropriate. All box plots need to be defined in the legends in terms of minima, maxima, centre, bounds of box and whiskers and percentile. | **✓** | |
| **4.** | The figure legends must indicate the statistical test used. Where appropriate, please indicate in the figure legends whether the statistical tests were one-sided or two-sided and whether adjustments were made for multiple comparisons. For null hypothesis testing, please indicate the test statistic (e.g. F, t, r) with confidence intervals, effect sizes, degrees of freedom and P values noted. Please provide the test results (e.g. P values) as exact values whenever possible and with confidence intervals noted. | | |
|  | **Legends requiring revision:**   1. Please indicate the statistical test used for data analysis and where appropriate, please specify whether it was one-sided or two-sided and whether adjustments were made for multiple comparisons, in the legends of figures: 5e; Supplementary figures: 3c-d, 3f, 4d, 6b, 7b, 8d. 8f, 10c. 2. Please note that the information on whether the statistical test used was one-sided or two-sided, where appropriate, is missing in the legends of figures: 5h, 6e, 7a, 7e; Supplementary figures: 4b, 10f. 3. Please note that the exact p value should be provided, when possible, in the legends of figures: 2k; Supplementary figures: 3h-i, 4b, 4e. | Ad 1.) and 2.) We added the requested information to the respective figure legends.  Ad 3.) In Fig. 2k and Supplementary Fig. 3h-i and 4e we report only an upper limit of *p* *<* 2e-16 as this is the lowest *p*-value computed by the software used. As such this limit can be regarded as very significant without specifying the exact value. In Supplementary Fig. 4b we would like to keep the simplified representation with *p*-value thresholds indicated by stars as the panel with 32 small plots is already quite crowded. | |
| **5.** | **Reproducibility:** Please state in the legends how many times each experiment was repeated independently with similar results. This is needed for all experiments, but is particularly important wherever results from representative experiments (such as micrographs) are shown. If space in the legends is limiting, this information can be included in a section titled “Statistics and Reproducibility” in the methods section. | | |
| **6.** | **Data availability:**This journal strongly supports public availability of data and custom code associated with the paper in a persistent repository where they can be freely and enduringly accessed or as a supplementary data file when no appropriate repository is available. If data and code can only be shared on request, please explain why in your data Availability Statement, and also in the correspondence with your editor. For more information, please refer to <https://www.nature.com/nature-research/editorial-policies/reporting-standards#availability-of-data> | | |
|  | Please ensure that datasets deposited in public repositories are now publicly accessible, and that accession codes or DOI are provided in the "Data Availability" section. As long as these datasets are not public, we cannot proceed with the acceptance of your paper. For data that have been obtained from publicly available sources, please provide a URL and the specific data product name in the data availability statement. Data with a DOI should be further cited in the methods reference section. | See Data Availability section. | |
| **7.** | **Flow cytometry data:** Please provide a Supplementary Figure to graphically account for all FACS sequential gating/sorting strategies, or provide gating/sorting strategies in-figure. If the former, please be sure to indicate, in the Supplementary Figure legend, which gating panel(s) correspond to which FACS data panel(s) in the manuscript figures. (For an example, please see <https://www.nature.com/articles/ncomms15067#supplementary-information>). | See above, FACS gating strategies are described in Supplementary Fig. 7d and Supplementary Fig. 9a. | |
